# A network model of basal ganglia for understanding the roles of dopamine and serotonin in reward-punishment-risk based decision making

**DOI:** 10.3389/fncom.2015.00076

**Published:** 2015-06-17

**Authors:** Pragathi P. Balasubramani, V. Srinivasa Chakravarthy, Balaraman Ravindran, Ahmed A. Moustafa

**Affiliations:** ^1^Department of Biotechnology, Indian Institute of Technology MadrasChennai, India; ^2^Department of Computer Science and Engineering, Indian Institute of Technology MadrasChennai, India; ^3^School of Social Sciences and Technology, Marcs Institute for Brain and Behavior, University of Western SydneyPenrith, NSW, Australia; ^4^Department of Veterans Affairs, New Jersey Health Care SystemEast Orange, NJ, USA

**Keywords:** serotonin, dopamine, basal ganglia network, risk, reward, punishment, medium spiny neurons, D1 and D2 receptor co-expression

## Abstract

There is significant evidence that in addition to reward-punishment based decision making, the Basal Ganglia (BG) contributes to risk-based decision making (Balasubramani et al., 2014). Despite this evidence, little is known about the computational principles and neural correlates of risk computation in this subcortical system. We have previously proposed a reinforcement learning (RL)-based model of the BG that simulates the interactions between dopamine (DA) and serotonin (5HT) in a diverse set of experimental studies including reward, punishment and risk based decision making (Balasubramani et al., 2014). Starting with the classical idea that the activity of mesencephalic DA represents reward prediction error, the model posits that serotoninergic activity in the striatum controls risk-prediction error. Our prior model of the BG was an abstract model that did not incorporate anatomical and cellular-level data. In this work, we expand the earlier model into a detailed network model of the BG and demonstrate the joint contributions of DA-5HT in risk and reward-punishment sensitivity. At the core of the proposed network model is the following insight regarding cellular correlates of value and risk computation. Just as DA D1 receptor (D1R) expressing medium spiny neurons (MSNs) of the striatum were thought to be the neural substrates for value computation, we propose that DA D1R and D2R co-expressing MSNs are capable of computing risk. Though the existence of MSNs that co-express D1R and D2R are reported by various experimental studies, prior existing computational models did not include them. Ours is the first model that accounts for the computational possibilities of these co-expressing D1R-D2R MSNs, and describes how DA and 5HT mediate activity in these classes of neurons (D1R-, D2R-, D1R-D2R- MSNs). Starting from the assumption that 5HT modulates all MSNs, our study predicts significant modulatory effects of 5HT on D2R and co-expressing D1R-D2R MSNs which in turn explains the multifarious functions of 5HT in the BG. The experiments simulated in the present study relates 5HT to risk sensitivity and reward-punishment learning. Furthermore, our model is shown to capture reward-punishment and risk based decision making impairment in Parkinson's Disease (PD). The model predicts that optimizing 5HT levels along with DA medications might be essential for improving the patients' reward-punishment learning deficits.

## Introduction

Decision making is related to choosing an action from a set of potential alternatives. The resulting rewarding or punitive outcomes can shape future decisions. In psychological terms, rewards and punishments represent opposite ends on the affective scale. Despite efforts to find dissociable brain systems that code for processing reward and punishment outcomes (Liu et al., 2011), a stringent division of brain systems in reward vs. punishment terms does not seem to be possible, since same neural regions respond to both reward and punishment (Rogers, 2011). The science of learning about the environment through outcomes (rewards and punishments) is called reinforcement learning (RL) (Sutton and Barto, 1998). We focus on a key area of the brain thought to implement reinforcement learning—the basal ganglia (Chakravarthy et al., 2010).

The Basal Ganglia (BG) are a set of nuclei situated in the forebrain known to be involved in a variety of functions, including action selection, action timing, working memory, and motor sequencing (Chakravarthy et al., 2010). A prominent approach that has been gaining consensus over the past decade seeks to model functions of the BG using the theory of RL (Joel et al., 2002). RL theory describes how an artificial agent or an animal learns stimulus-response relationships that maximize rewards obtained from the environment. According to this theory, stimulus-response associations with rewarding outcomes are reinforced, while those that result in punishments are attenuated. Experimental studies show that the activity of dopamine (DA) releasing mesencephalic nucleus-substantia nigra pars compacta (SNc) resembles an RL-related quantity called Temporal Difference (TD) error. TD error represents the difference between the total reward that an animal actually obtains and its expectation of the same, and is a key variable that controls learning in RL framework. This insight has inspired extensive modeling work to apply concepts from RL for describing functions of the BG (Joel et al., 2002). RL theory has been able to account for many crucial functions of DA in BG- mediated learning and behavior (Houk et al., 2007; Schultz, 2010a). Classical models of the BG cast their dynamics in a value function based decision making framework, where value function is the expectation of observed rewards (Joel et al., 2002; Frank et al., 2004; Krishnan et al., 2011). We showed in a recent study (Balasubramani et al., 2014) that BG dynamics can be better modeled using utility based decision making framework mediated by the neuromodulators DA and serotonin (5HT). In that abstract model (Balasubramani et al., 2014), the activity of 5HT controlled the combination of value and risk function for the computation of utility, where risk is the variance observed in the outcomes. The model was shown to reconcile three diverse and representative theories that seek to associate 5HT to (1) punishment sensitivity; (2) time scale of reward prediction; and (3) risk-sensitivity. According to the first theory, central 5HT modulates punishment prediction differentially from reward prediction (Cools et al., 2008). Artificial reduction of 5HT by reducing the levels of tryptophan in the body decreased the tendency to avoid punishment (Cools et al., 2011). A second theory of 5HT function associates its activity to the time scale of reward prediction. This theory is based on experiments which showed that under conditions of low 5HT, subjects exhibited impulsivity—a tendency to choose short-term rewards over the long-term ones (Tanaka et al., 2007). The third theory relates 5HT to risk-sensitivity. Low levels of 5HT promote risk seeking behavior when provided with choices of equal mean and different variances (risk) associated with the outcomes (Long et al., 2009; Murphy et al., 2009).

The current study presents a neural network model of the BG including nuclei such as striatum, subthalamic nucleus (STN) and globus pallidum (externa and interna -GPe/GPi), and is controlled by neuromodulators such as DA and 5HT. The model builds on a novel proposal that the medium spiny neurons (MSNs) of the striatum can compute either value or risk depending on the types of DA receptors they express. While the MSNs that express DA D1-receptor (D1R) compute value as earlier suggested in modeling studies (O'Doherty et al., 2004), those that co-express D1R and D2R are now shown to be capable of computing risk. No earlier computational models of the BG (Frank et al., 2004; Ashby et al., 2010; Humphries and Prescott, 2010; Krishnan et al., 2011) have taken these D1R-D2R co-expressing neurons into consideration, though their existence in the BG was shown by many experiments (Nadjar et al., 2006; Bertran-Gonzalez et al., 2010; Hasbi et al., 2010, 2011; Perreault et al., 2010, 2011; Calabresi et al., 2014). The neuromodulator DA is represented as the TD error mediating either the update of the cortico-striatal weights or the action selection dynamics occurring downstream of the striatum. This is in agreement to various contemporary models of DA in the BG (Frank et al., 2004; Magdoom et al., 2011; Kalva et al., 2012; Chakravarthy and Balasubramani, 2014). The specific modulation site of 5HT in the striatum is elusive (Ward and Dorsa, 1996; Eberle-Wang et al., 1997; Barnes and Sharp, 1999; Nicholson and Brotchie, 2002; Parent et al., 2011). This study makes a prediction on the types of striatal MSNs that significantly receive 5HT modulation. It describes the computational roles of the three pools of striatal MSNs viz., D1R-expressing, D2R-expressing and D1R-D2R co-expressing MSNs. It also expands the earlier BG architectures significantly by ascribing a crucial role to the D1R-D2R MSNs that project to the direct and indirect pathways of the BG. The presented DA-5HT mediated network model is then shown to explain their seminal behavioral effects by simulating experiments analyzing reward, punishment, and risk learning (Daw et al., 2002; Cools et al., 2008; Long et al., 2009). The study also extends toward describing a principal model of the BG dysfunction i.e., Parkinson's Disease (PD) for explaining the associated impairment in action selection (Bodi et al., 2009).

The paper is organized as follows: Section A Model of Utility-based Decision Making outlines the lumped model of value and risk computation in the striatum as described in our earlier study (Balasubramani et al., 2014). Section Cellular Correlates for the Value and the Risk Computation describes the neural correlates for both the value and risk computation in the striatum. Specifically, this section shows that D1R expressing MSNs are involved in value computation, while the MSNs that co-express D1R and D2R support risk computation. The network model is introduced in Section Modeling the BG Network in Healthy Control Subjects that uses the neural correlate model of Section Cellular Correlates for the Value and the Risk Computation for the BG action selection dynamics. The D1R MSNs project to GPi via the Direct Pathway (DP) while the D1R-D2R and the D2R MSNs project to GPi via the Indirect Pathway (IP) consisting of the GPe and STN. The SNc model component receives input from both D1R MSNs and D1R-D2R MSNs, and releases DA. The experimental sections deal with testing the model on risk sensitivity (Section Modeling the Risk Sensitivity), punishment sensitivity and behavioral inhibition (Section Modeling Punishment Mediated Behavioral Inhibition). The model is further extended to simulate PD condition. Section Modeling the Reward-punishment Sensitivity in PD thereby studies the model behavior on a probabilistic reward-punishment learning paradigm in control and PD conditions. The model equations that are adapted to represent the PD condition are given in the Section Simulating Parkinson's Disease (PD). The study results, limitations and testable predictions are finally discussed in Section Discussion.

## Model

### A model of utility-based decision making

This section quickly summarizes our extended reinforcement learning model of the BG (Balasubramani et al., 2014), where the agent (subject) tends to maximize utility. We start with the value function “*Q*,” associated with a state, “*s*,” and an action, “a,” pair, at time, “*t.*” This is the expected discounted sum of rewards obtained starting from time *t* in state *s*:

(2.1.1) $$Q^{\pi }(s,a)=E_{\pi }(r_{t+1}+\gamma r_{t+2}+\gamma^{2}r_{t+3}+\ldots |s_{t}=s,a_{t}=a)$$

where, γ, is a discount factor controlling the myopicity of the rewards. These value functions are updated using the temporal difference learning rule as follows:

(2.1.2) $$Q_{t+1}(s_{t},a_{t})=Q_{t}(s_{t},a_{t})+\eta_{Q}\delta_{t}$$

where, “δ_*t*_” is the temporal difference (TD) error, given by Equation (2.1.3) if the experiment runs for multiple time steps, and by Equation (2.1.4) in the case of single-step experiments.

(2.1.3) $$\delta_{t}\;=r_{t}+\gamma Q_{t}\left(s_{t+1},a_{t+1}\right)-Q_{t}\left(s_{t},a_{t}\right)$$

(2.1.4) $$\delta_{t}\;=r_{t}-Q_{t}\left(s_{t},a_{t}\right)$$

We introduced the notion of a risk function, “*h*,” that tracks the *variance* (δ^2^) (Bell, 1995; D'Acremont et al., 2009) in instantaneous rewards or the reward prediction error with zero mean, and is updated as follows:

(2.1.5) $$h_{t+1}(s_{t},a_{t})=h_{t}(s_{t},a_{t})+\eta_{h}\xi_{t}$$

where, ξ_*t*_ is the risk prediction error given by:

(2.1.6) $$\xi_{t}=\delta_{t}^{2}{-\text{h}}_{t}({\text{s}}_{t},\;a_{t}).$$

Finally, we define the utility “*U*,” at time, “*t*,” as a combination of the value function and the risk function as follows:

(2.1.7) $$U_{t}(s_{t},a_{t})=Q_{t}(s_{t},a_{t})-\alpha \;sign(Q_{t}(s_{t},a_{t}))\;\sqrt{h_{t}(s_{t},a_{t})}$$

where, α controls the risk sensitivity and is proposed to represent the functioning of 5HT in the BG. The *sign*() term in Equation (2.1.7) represents the non-linear risk sensitivity. Studies show that the subjects are risk averse in the case of gains and risk seeking during losses (Kahneman and Tversky, 1979). The subjective gains (losses) are represented by a positive (negative) value of *Q*; and therefore the risk component with the *sign*(*Q*) would negatively (positively) affect the Utility, in order to show risk averse (seeking) behavior. The policy used for utility maximization is *soft-max*, with the probability, “*P*,” of choosing an action from a state at time, “*t*,” given by the following Equation (2.1.8):

(2.1.8) $$P_{t}(a|s)=\exp(\beta U_{t}(s,a))/\sum_{i=\text{1}}^{n}\exp(\beta U_{t}(s,i))$$

“*n*” is the total number of actions available at state, “*s*,” and “β” is the inverse temperature parameter. Values of β tending toward 0 make the actions almost equiprobable whilst values tending toward ∞ make the soft-max action selection identical to greedy action selection.

This utility-based model of the BG described by Balasubramani et al. (2014) is an abstract, lumped model in which it is proposed that the utility function is computed in the striatum. However, in order to expand the lumped model to a network version, we first identify cellular correlates of value and risk computations in the next section.

### Cellular correlates for the value and the risk computation

Most approaches to modeling cellular level mechanisms for value computation in the striatum consist of three conditions:

1. Occurrence of TD error information in the form of DA signals in at the striatum (Schultz et al., 1997),
2. Availability of information related to the cortical sensory state in the striatum (Divac et al., 1977; Mcgeorge and Faull, 1989), and
3. DA-dependent plasticity in cortico-striatal connections (Reynolds and Wickens, 2002).

A typical formulation of DA-dependent learning (Reynolds and Wickens, 2002) may be expressed as the change in cortico-striatal connection strength, *w* (Δ*w*),

(2.2.1) Δw=ηδx

Where “*x*” in Equation (2.2.1) represents the cortical sensory input and is used in this section as a logical variable for neural encoding of the underlying state “*x*,” *x* = 1 (if *x* = *s*_*t*_) *else x* = 0; “δ” is the TD error [Equations (2.1.3, 2.1.4): representing DA activity]; and “η” is the learning rate. Similar formulations have been proposed from purely RL-theory considerations (See Chapter 9 of Abbott, 2001). A slight variation of the above equation would be as follows.

(2.2.2) $$\Delta w=\eta \lambda^{Str}(\delta )x$$

where “λ^*Str*^” is a function of δ, that represents the effect of DA on the striatal neural firing rate (Reynolds and Wickens, 2002). Thus, the learning rule of Equation (2.2.2) has a Hebb-like form, where the neuro-modulation is modeled in terms of the effect of the neuromodulator on the firing rate of the post-synaptic neuron. The form of the function λ^*Str*^ varies depending on the type of DA family receptors (R) expressed in Medium Spiny Neurons (MSNs) as explained below. In neurons with D1R expression, higher DA level increases the probability of MSN excitation by a given cortical input (Moyer et al., 2007; Surmeier et al., 2007). Hence, in models that represent MSNs, λ^*Str*^ is described as an increasing sigmoid function of DA for neurons that express D1R. In cells with D2R, the activation is higher under conditions of low DA levels (Hernandez-Echeagaray et al., 2004) and therefore the λ^*Str*^ function is modeled as a decreasing function of DA (Frank, 2005; Frank et al., 2007a). These sigmoid λ^*Str*^ functions are expressed as:

(2.2.3) $$\begin{array}{l} \lambda_{D1}^{Str}(\delta )=\frac{2c_{1}}{1+\exp(c_{2}(\delta +c_{3}))}-c_{1}\\ \lambda_{D2}^{Str}(\delta )=\frac{2c_{1}}{1+\exp(c_{2}(\delta +c_{3}))}-c_{1}\\ \lambda_{h-D1}^{Str}(\delta )=\frac{c_{1}}{1+\exp(c_{2}(\delta +c_{3}))}\\ \lambda_{h-D2}^{Str}(\delta )=\frac{c_{1}}{1+\exp(c_{2}(\delta +c_{3}))} \end{array}$$

where c_1_, c_2_, c_3_ are constants subject to the receptor type, and represent the nature of the receptors; The gain functions of D1R MSNs, D2R MSNs are given by λ^*Str*^_*D*1_, λ^*Str*^_*D*2_, and that of the D1R and the D2R component of co-expressing MSNs are given by λ^*Str*^_*h*−*D*1_, λ^*Str*^_*h*−*D*2_, respectively.

**Figure 1:**
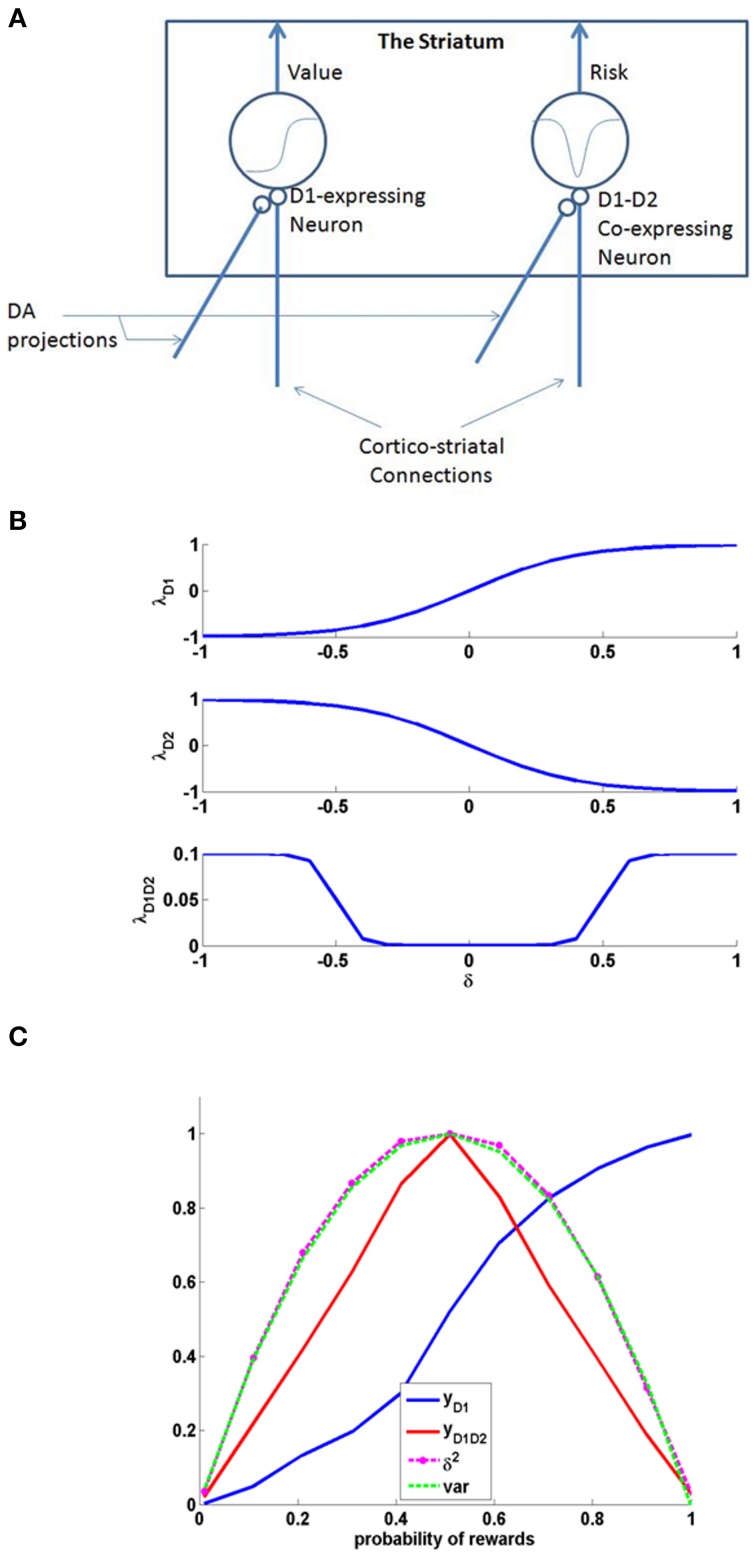
**(A)** Schematic of the cellular correlate model for the value and the risk computation in the striatum, **(B)** The D1, D2, and D1D2 gain functions, **(C)** The output activity of D1R MSN (yD1), D1R-D2R co-expressing MSN (yD1D2), variance tracked through Equation (2.1.5) containing δ2, and normalized variance computed analytically (var) = p^*^(1-p); Here p is the probability associated with rewards, i.e., with probability p, reward = 1, else reward = 0. The resemblance of var to yD1D2 shows the ability of D1R-D2R co-expressing MSN to perform risk computation.

Examples for such sigmoid λ functions with parameters (Table 1) for the D1R, D2R, and the D1R-D2R MSNs are shown in (Figure 1B).

**Table 1:** **Parameters used in Equation (2.2.3) for Figure 1**.

	**λ^***Str***^_***D*1**_**	**λ^***Str***^_***h*−*D*1**_**	**λ^***Str***^_***h*−*D*2**_**
c_1_	1	0.1	0.1
c_2_	−5	−25	25
c_3_	0	−0.5	0.5

The activity of MSNs with D1R expression (y_*D*1_) are appropriately suited for value computation (Krishnan et al., 2011; Kalva et al., 2012). They express λ_*D*1_(δ) as an increasing function of δ. The D1R MSN's activity can be thought as a network equivalent of the Equation (2.1.2) in abstract model.

The D1R MSNs receive cortico-striatal connections whose weight is denoted by “*w*_*D*1._” The value “*Q*” computed from such an MSN's activity (*y*_*D*1_) is given by Equation (2.2.4).

(2.2.4) $$y_{D1}\;=\;w_{D1}x\;and\;Q=y_{D1}$$

And change in weight for such a neuron is given by Equation (2.2.5).

(2.2.5) $$\Delta w_{D1}=\eta_{D1}\;\lambda_{D1}^{Str}(\delta )\;x$$

where η_*D*1_ is the learning rate.

A similar neuron model in which D1R and D2R are co-expressed can simulate risk computations. In case of a neuron that would compute risk, the λ^*Str*^ function is represented as “λ^*Str*^_*D*1*D*2_.”It was reported that the behavior of D1R-D2R co-expressing neurons may be described as the sum of the antagonistic actions of D1 and the D2 expressing neurons (refer to the discussion section for more details). Therefore, activation of D1R-D2R MSNs (*y*_D1D2_) could be modeled simply as an addition of the effects of independent activations of D1R and D2R MSNs, respectively (Surmeier et al., 2007; Allen et al., 2011; Hasbi et al., 2011). When their activation function is computed as a simple summation (superposition) of D1R and D2R MSNs, they capture the variance associated with the rewards and thereby form the risk function (Figure 1). The function “λ^*Str*^_*D*1*D*2_” of D1R-D2R MSNs is an even function of “δ,” with λ^*Str*^_*D*1*D*2_ (δ) increasing with increasing *magnitude* of δ, thereby increases with δ^2^. The λ^*Str*^_*D*1*D*2_ Equation (2.2.6) can be expressed as the summation of functions corresponding to a D1R component (λ^*Str*^_*h*−*D*1_) and a D2R component (λ^*Str*^_*h*−*D*2_) as follows:

(2.2.6) $$\lambda_{D1D2}^{Str}=\lambda_{h-D1}^{Str}+\lambda_{h-D2}^{Str}$$

Note that the characteristics of λ^*Str*^_*h*−*D*1_ and λ^*Str*^_*h*−*D*2_ as a function of δ depend on the constants c_1_, c_2_, c_3_ of Equation (2.2.3). Response (*y*_*D*1*D*2_) of such a neuron is given as,

(2.2.7) $$y_{D1D2}\;=\;w_{D1D2}x\;and\;h=y_{D1D2}$$

and the change in corresponding weight, Δ*w_*h*_*, is given as,

(2.2.8) $$\Delta w_{D1D2}=\eta_{D1D2}\;\lambda_{D1D2}^{Str}(\delta )\;x$$

where η_*D*1*D*2_ is the learning rate. The (D1R-expressing) striatal MSNs with δ-dependent λ^*Str*^ functions that are of increasing sigmoidal shape are capable of computing value. Similarly (D1R-D2R co-expressing) striatal neurons with δ-dependent λ^*Str*^ functions that are “U” shaped, can compute risk (Figure 1). The gain expression for risk coding MSNs (λ^*Str*^_*h*−*D*1_, λ^*Str*^_*h*−*D*2_) uses a logarithmic-sigmoid function that is unipolar, while the gain expression of other D1R-, D2R- MSNs (λ^*Str*^_*D*1_, λ^*Str*^_*D*2_) uses a tangent-sigmoid function that is bipolar Equation (2.2.3).

**Figure 2:**
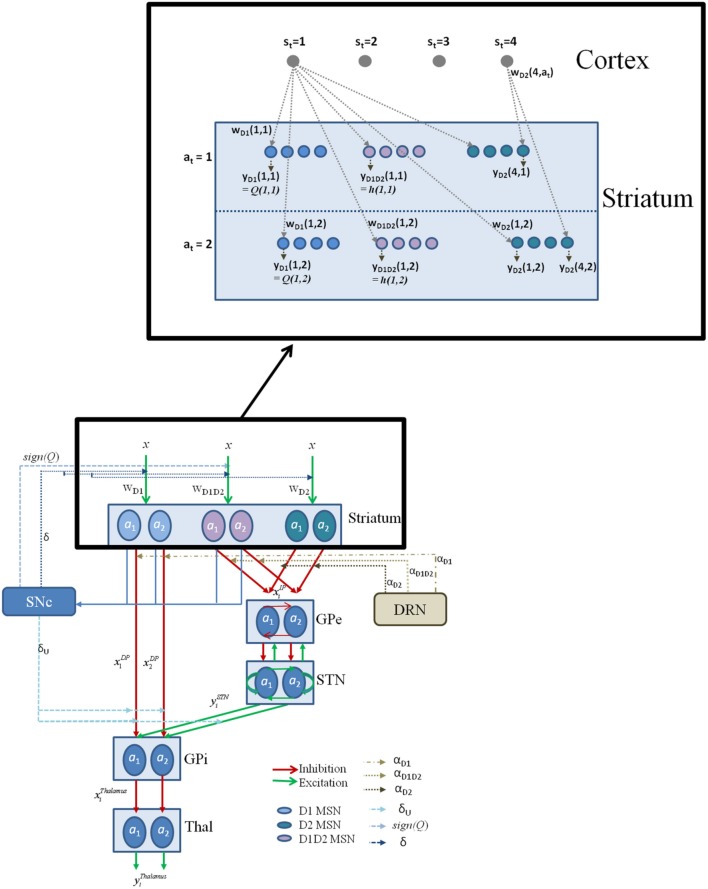
**The schematic flow of the signal in the network model**. Here *x* denotes the presence of a state; *a* denotes the action; with the subscript denoting the index *i*; Since most of the experiments in the study simulate two possible actions for any state, we depict the same in the above figure for a state *s*_*i*_; The D1, D2, D1D2 represent the D1R-, D2R-, D1R-D2R MSNs, respectively, and *w* denotes subscript- corresponding cortico-striatal weights. The schematic also have the representation of DA forms: (1) The δ affecting the cortico-striatal connection weights (Schultz et al., 1997; Houk et al., 2007), (2) The δ_U_ affecting the action selection at the GPi (Chakravarthy and Balasubramani, 2014), (3) The Q affecting the D1/D2 MSNs (Schultz, 2010b); and 5HT forms represented by α_D1_, α_D2_, and α_D1D2_ modulating the D1R, D2R, and the D1R-D2R co-expressing neurons, respectively. The inset details the notations used in model section for representing cortico-striatal weights (*w*) and responses (*y*) of various kinds of MSNs (D1R expressing, D2R expressing, and D1R-D2R co-expressing) in the striatum, with a sample cortical state size of 4, and maximum number of action choices available for performing selection in every state as 2.

Just as D1R expressing MSNs can be regarded as cellular level substrates for value computation in the striatum, D1R-D2R co-expressing MSNs are suitable to be cellular level substrates for risk computation [Figures 1, 2 (inset)]. The D1R-D1R co-expressing MSN's activity can be thought as a network equivalent of the Equation (2.1.5) in abstract model. Particularly, the even property of their activation as a function of δ is essential to capture the variance associated with rewards (Figure 1C).

We now introduce the above cellular substrates for value and risk computation in a network model of the BG and show that the network is capable of reward-punishment-risk based decision making.

### Modeling the BG network in healthy control subjects

The cellular level substrates for value and risk computation in the BG, described above, are now incorporated into a network model of the BG. This model captures the anatomical details of the BG and represents the following nuclei (described in the Section Cellular Correlates for the Value and the Risk Computation)—the striatum, STN, GPe and GPi. The training of the cortico-striatal connections by nigro-striatal DA correlate (δ) also occurs as described in the earlier Section Cellular Correlates for the Value and the Risk Computation. It models, in an elementary form, the action of DA in switching between DP and IP, via the differential action of DA on the D1, D2, and D1-D2 co-expressing receptors (R) of striatal MSNs. The model also claims different DA signals for the updating of cortico-striatal weights and the switching in GPi (Chakravarthy and Balasubramani, 2014). Some of the key properties of the STN-GPe system such as their bi-directional connectivity facilitating oscillations and “Exploratory” behavior are also captured.

The equations for the individual modules of the proposed network model of the BG (Figure 2) are as follows:

#### Striatum

The Striatum is proposed to have three types of MSNs: D1R expressing, D2R expressing, and D1R-D2R co-expressing MSNs, all of which follow the model described in Section Cellular Correlates for the Value and the Risk Computation. The cortico-striatal weight update equations for different types of neurons (with subscripts—D1, D2, and D1D2: for the D1R expressing, D2R expressing, and D1R-D2R co-expressing MSNs, respectively) with the gain function (λ^*Str*^_*D*1_, λ^*Str*^_*D*2_, λ^*Str*^_*D*1*D*2_, respectively) as given by Equation (2.2.3), would then be:

(2.3.1) $$\begin{array}{l} \Delta w_{D1}(s_{t},a_{t})=\eta_{D1}\lambda_{D1}^{Str}(\delta (t))x\\ \Delta w_{D2}(s_{t},a_{t})=\eta_{D2}\lambda_{D2}^{Str}(\delta (t))\;x\\ \Delta w_{D1D2}(s_{t},a_{t})=\eta_{D1D2}\lambda_{D1D2}^{Str}(\delta (t))\;x \end{array}$$

Each state-action (*s*-*a*) pair is associated with a cortico-striatal weight Equation (2.3.1). The weight corresponding to the encountered *s* and *a*, at a time *t*, is then updated using Equation (2.3.1). The λ^*Str*^ gain function for the D1R, D2R, D1R-D2R MSNs are the same as in Equation (2.2.3). The δ in the weight update equations is given by Equation (2.3.2) to capture the immediate reward conditions:

(2.3.2) $$\delta (t)=r-Q_{t}(s_{t},a_{t})$$

η_D1_, η_D2_, η_D1D2_ are the learning rates for the D1R, D2R and the D1R-D2R MSN cortico-striatal weights, respectively. The “*Q*” function as calculated in the previous section would be computed by the output of D1R MSNs as in Equation (2.3.3).

(2.3.3) $$\begin{array}{l} \;Q_{t}(s_{t},a_{t})=y_{D1}(s_{t},a_{t})\\ where\;y_{D1}(s_{t},a_{t})=w_{D1}(s_{t},a_{t})\;x \end{array}$$

The risk function (*h*_*t*_) associated with choosing each action, *a_*t*_* is then calculated by Equation (2.3.4)

(2.3.4) $$\begin{array}{l} \;h_{t}(s_{t},a_{t})=y_{D1D2}(s_{t},a_{t})\\ where\;y_{D1D2}(s_{t},a_{t})=w_{D1D2}(s_{t},a_{t})\;x \end{array}$$

For a conservative development of a network model from the earlier mentioned abstract level model of Section A Model of Utility-based Decision Making, the utility function for a state-action pair can be written as Equation (2.3.5).

(2.3.5) $$U_{t}(s_{t},a_{t})=Q_{t}(s_{t},a_{t})-\alpha_{D1D2}\;sign(Q_{t}(s_{t},a_{t}))\;\sqrt{h_{t}(s_{t},a_{t})}$$

The change in utility is calculated using Equation (2.3.6).

(2.3.6) $$\delta_{U}(t)=U_{t}(s_{t},a_{t})-U_{t-1}(s_{t},a_{t-1})$$

Here α_D1D2_ in Equation (2.3.5) denotes the modulation of 5HT particularly on the D1R-D2R co-expressing MSNs which computes the risk value “h.” More details on modeling 5HT modulation are described later in this section.

#### STN-GPe system

In the STN-GPe model, STN and GPe layers have equal number of neurons, with each neuron in STN uniquely connected bi-directionally to a neuron in GPe. Both STN and GPe layers are further assumed to have weak lateral connections within the layer. A more detailed description of this model can be obtained from Chakravarthy and Balasubramani (2014). The number of neurons in the STN (or GPe) (Figure 2) is taken to be equal to the number of possible actions for any given state (Amemori et al., 2011; Sarvestani et al., 2011). The dynamics of the STN-GPe network is given below

(2.3.7) $$\begin{array}{l} \tau_{s}\frac{dx_{i}^{STN}}{dt}=-x_{i}^{STN}+\sum_{j\;=\;m1}^{n}W_{ij}^{STN}y_{i}^{STN}-x_{i}^{GPe}\\ \;y_{i}^{STN}=tanh(\lambda^{STN}x_{i}^{STN})\\ \tau_{g}\frac{dx_{i}^{GPe}}{dt}=-x_{i}^{GPe}+\sum_{j\;=\;1}^{n}W_{ij}^{GPe}x_{i}^{GPe}+y_{i}^{STN}-x_{i}^{IP} \end{array}$$

*x*^*GPe*^_*i*_ - internal state (same as the output) representation of *i*th neuron in GPe;

*x*^*STN*^_*i*_ - internal state representation of *i*th neuron in STN, with the output represented by *y*^*STN*^_*i*_;

*W*^*GPe*^ - lateral connections within GPe, equated to a small negative number ϵ_g_ for both the self (*i* = *j*) and non-self (*i* ≠ *j*) connections for every GPe neuron.

*W*^*STN*^ - lateral connections within STN, equated to a small positive number ϵ_s_ for all non-self (*i* ≠ *j*) lateral connections, while the weight of self-connection (*i* = *j*) is equal to 1 + ϵ_*s*_, for each STN neuron *i*.

We assume that both STN and GPe have complete internal connectivity, where every neuron in the layer is connected to every other neuron in the same layer, with the same connection strength. That common lateral connection strength is ϵ_*s*_ for STN, and ϵ_*g*_ for GPe. Likewise, STN and GPe neurons are connected in a one-to-one fashion—the *I*'th neuron in STN is connected to the *i*'th neuron in GPe and vice-versa. For all simulations presented below, the parameters: ϵ_g_ = −ϵ_s_ = 0.1; the step-sizes: 1/τ_S_ = 0.1; 1/τ_g_ = 0.033; and the slope: λ^STN^ = 3;

#### Striatal output toward the direct (DP) and the indirect pathway (IP)

Assuming that the striatal D1R MSNs project via the DP to GPi (Albin et al., 1989; Frank, 2005; Chakravarthy et al., 2010), the contribution of the DP to GPi is given by:

(2.3.8) $$x_{i}^{DP}=\;\alpha_{D1}\;\lambda_{D1}^{GPi}(\delta_{U}(t))\;y_{D1}(s_{t},a_{t})\;$$

The GPe is modeled to receive inputs from both the D2R and D1R-D2R MSNs of the striatum (Hasbi et al., 2011; Perreault et al., 2011; Wallman et al., 2011; Balasubramani et al., 2014) in the indirect pathway. The input to the GPe is therefore given by:

(2.3.9) $$\begin{array}{l} x_{i}^{IP}=\alpha_{D2}\;\lambda_{D2}^{GPi}(\delta_{U}(t))\;y_{D2}(s_{t},a_{t})\;+\alpha_{D1D2}\;sign(y_{D1}(s_{t},a_{t}))\\ \;\lambda_{D1D2}^{GPi}(\delta_{U}(t))\sqrt{y_{D1D2}(s_{t},a_{t})\;} \end{array}$$

where the response functions of various kinds of MSNs are denoted by variable “*y*”:

$$\begin{array}{l} y_{D1}(s_{t},a_{t})\;=w_{D1}(s_{t},a_{t})\;x\\ y_{D2}(s_{t},a_{t})\;=w_{D2}(s_{t},a_{t})\;x\\ y_{D1D2}(s_{t},a_{t})\;=w_{D1D2}(s_{t},a_{t})\;x \end{array}$$

and

$$\begin{array}{l} \lambda_{D1}^{GPi}(\delta_{U})=\frac{2c_{1}}{1+\exp(c_{2}(\delta_{U}+c_{3}))}-c_{1}\\ \lambda_{D2}^{GPi}(\delta_{U})=\frac{2c_{1}}{1+\exp(c_{2}(\delta_{U}+c_{3}))}-c_{1}\\ \lambda_{h-D1}^{GPi}(\delta_{U})=\frac{c_{1}}{1+\exp(c_{2}(\delta_{U}+c_{3}))}\\ \lambda_{h-D2}^{GPi}(\delta_{U})=\frac{c_{1}}{1+\exp(c_{2}(\delta_{U}+c_{3}))} \end{array}$$

It should also be noted that λ^*Str*^*s* used as gain factors for the striatal neural outputs of Equations (2.3.8, 2.3.9) are different from that used in Equation (2.3.1). The λ s used in weight dynamics of Equation (2.3.1) are dependent on the TD error of Equation (2.3.2) in immediate reward condition. Whereas, DA used in the λ^*GPi*^ of Equations (2.3.8, 2.3.9) is different—it is the temporal gradient of *U* [δ_U_: Equation (2.3.6)] which has a direct role in switching between DP and IP (Kliem et al., 2007). The temporal difference in utility function between time *t* and *t-*1 is modeled to control exploitation and exploration dynamics of action selection (Balasubramani et al., 2015) in the BG as follows. In the case of δ_U_ being high, then according to Equation (2.3.6), the action at time, *t*, has a higher utility compared to that at time, *t-*1. This case facilitates DP Equation (2.3.8) that is popularly dubbed as *Go* pathway which exploits by selecting the same action *a*_*t*_. In contrary, if δ_U_ is low, then the *NoGo* pathway (IP) is selected Equation (2.3.9) for facilitating the action taken at time, *t*−1. This is because the action at time, *t*−1, has a higher utility compared to that at time, *t* Equation (2.3.6). In the third case of δ_U_ between high and low levels, a random selection of choice from the action repertoire is made, by the *Explore* pathway (IP) (Chakravarthy and Balasubramani, 2014). Further, DAergic neural activity in monkeys is recently found to be well correlating to the computed utility-difference at a time, *t*, while performing a decision making task (Stauffer et al., 2014).

In the lumped model of Section A Model of Utility-based Decision Making (Balasubramani et al., 2014), the parameter α represents 5HT activity Equation (2.1.7). The following can be realized on carrying over the concept to a network version. Since α controls risk term only in Equation (2.1.7), and it is shown in Section Cellular Correlates for the Value and the Risk Computation that D1R-D2R co-expression MSNs compute risk, it is natural to formulate the network model such that α modulates only the D1R-D2R MSNs in the striatum. However, experimental evidence to support such specificity in 5HT modulation of striatal neurons is unavailable (Refer to the Discussion section for details). Concerning the unspecific nature of 5HT action in the striatum, we introduce three α's in this section, to differentially module D1R, D2R and D1R-D2R MSNs, respectively. Precisely, 5HT α in Equation (2.1.7) is modeled as the parameters α_*D*1_ Equation (2.3.8), α_*D*2_, and α_*D*1*D*2_ Equation (2.3.9), for representing its differential modulation on D1R, D2R and the D1R-D2R MSNs, respectively (Figure 2, Table 2). The α's are optimized for each experimental condition separately.

**Table 2:** **The model correlates for DA and 5HT**.

**Neuromodulator**	**Model correlate**	**Description**	
DA	δ	Updating cortico-striatal weights (Schultz et al., 1997; Houk et al., 2007)	Equation (2.3.2)
	δ_U_	Switching between DP and IP–action selection dynamics (Stauffer et al., 2014)	Equation (2.3.6)
	*sign*(*Q*)	Controlling the risk sensitivity of utility based decision making (Schultz, 2010a,b)	Equation (2.3.9)
5HT	α_*D*1_	Controlling differential modulation of 5HT on *D1R, D2R and the D1R-D2R MSNs* (Ward and Dorsa, 1996; Eberle-Wang et al., 1997; Di Matteo et al., 2008b)	Equation (2.3.8)
	α_D2_		Equation (2.3.9)
	α_D1D2_		Equation (2.3.9)

The outputs of D1R and D2R MSNs to GPi flow via the DP and IP, respectively (O'Doherty et al., 2004; Amemori et al., 2011; Chakravarthy and Balasubramani, 2014). We propose that D1R-D2R MSNs also project to GPi via the IP (Perreault et al., 2010, 2011). The first term on the RHS of Equation (2.3.9) denotes projections from D2R expressing MSNs to GPe, whereas the second term represents projections from D1R-D2R co-expressing MSNs to the same target. The second term is analogous to the risk term in the utility function of Equation (2.1.7) (Balasubramani et al., 2014). This term contributes to the non-linear risk sensitivity, i.e., being risk-aversive in the case of gains as outcomes, and being risk-seeking during losses (Kahneman and Tversky, 1979).

The different forms of DA signals used in this study along with references to their biological plausibility are summarized as follows (Figure 2, Table 2):

1. Representing the TD error used in updating the cortico-striatal weights of the MSNs Equation (2.3.2), as reported by many experimental studies (Schultz et al., 1997; Reynolds and Wickens, 2002; Houk et al., 2007).
2. Representing the temporal gradient of the utility function [: = δ_U_ Equation (2.3.6)], used for switching between DP and IP (Chakravarthy and Balasubramani, 2014). For such a DA signal (: = δ_U_) from the SNc, those neurons might be using the information of the value component received due to the D1R MSN projections from striatum to SNc (Schultz et al., 1997; Doya, 2002; Houk et al., 2007), and the risk component from the projections of D1R-D2R MSNs to SNc (Surmeier et al., 1996; Perreault et al., 2010, 2011). Further, there are evidences for D1R MSNs and the co-expressing D1R-D2R MSNs forming the striosomal component that could assist in computing the utility prediction error from SNc (Jakab et al., 1996; Surmeier et al., 1996; Nadjar et al., 2006; Amemori et al., 2011; Calabresi et al., 2014). This form of DA signal is reported by a recent study on utility based decision making in monkeys by Schultz and colleagues (Stauffer et al., 2014).
3. The neurobiological interpretation of the *sign*(*Q*) used in the second term of the Equation (2.3.9) could be also linked to the SNc functioning. The “value function” coding DA neurons (represented by the projections marked by “Q” in the Figure 2) as reported in studies by Schultz and colleagues (Schultz, 2010b) might be preferentially targeting the D1R-D2R co-expressing neurons in the striatum. This modulation is roughly captured in our model through the *sign*(*Q*) term in Equations (2.3.5, 2.3.9).

#### Combining DP and IP in GPi

Each action neuron in GPi is modeled to combine the contributions of DP and IP (Kliem et al., 2007) as given in Equation (2.3.10),

(2.3.10) $$x_{i}^{GP_{i}}=-x_{i}^{DP}+w_{i}^{STN-Gpi}y_{i}^{STN}$$

where *x^*DP*^* is from Equation (2.3.8), and *y^*STN*^* that denotes output of STN, is from Equation (2.3.7). The relative weightage of STN projections to GPi, compared to that of the DP projections, is represented by *w*^*STN*−*GPi*^. For the simulations in this study, *w*^*STN*−*GPi*^ is set to 1 for all the GPi neurons.

#### Action selection at thalamus

The direct and indirect pathway is combined downstream either in GPi, or further along in the thalamic nuclei, which receive afferents from GPi (Humphries and Gurney, 2002; Chakravarthy et al., 2010). GPi neurons project to thalamus over inhibitory connections. Hence the thalamic afferents for a neuron *i*, may be expressed simply as,

(2.3.11) $$x_{i}^{Thalamus_{i}}=x_{i}^{DP}-w_{i}^{STN-Gpi}y_{i}^{STN}$$

These afferents activate thalamic neurons as follows,

(2.3.12) $$\frac{dy_{i}^{Thalamus}}{dt}=-y_{i}^{Thalamus}+x_{i}^{Thalamus}$$

where *y*^*Thalamus*^_*i*_ is the state of the *i*th thalamic neuron. Action selected is simply the “*i*” (*i* = 1, 2, .., n) whose *y*^*Thalamus*^_*i*_ is maximum on integration. In our simulations, the integration process is carried over for 25 time steps.

### Simulating parkinson's disease (PD)

A model of PD may incorporate the following features in terms of DA and 5HT levels:

1. DA levels are lower in PD than in controls: This feature is simulated by clamping “δ,” and upper bounding δ to δ_Lim_. Since there is a reduced number of DA cells, Substantia Nigra pars compacta (SNc) is thought to be capable of producing a weak signal reliably, but the highest firing levels in PD are smaller compared to controls (Kish et al., 1988).
2. PD medication (L-dopa, DA agonists) facilitates DA activity. This is simulated by simply adding a fixed constant to the preexisting clamped δ (Dauer and Przedborski, 2003; Foley et al., 2004).

Hence, to represent the PD condition, the Equation (2.3.2) describing DA activity is first clamped to δ_Lim_, as in Equation (2.4.1):

(2.4.1) $$if\;\delta >\delta_{Lim};\;\delta =\delta_{Lim}$$

Equation (2.4.1) represents the never-medicated case (PD-OFF). In the recently-medicated case (PD-ON), in addition to the clamping step (to δ_Lim_) just described, there is a transient increase in DA (to model the medication factor δ_*Med*_) to the clamped δ, which is implemented as:

(2.4.2) $$\delta :=\delta_{+}\delta_{Med}$$

This altered δ, that represents any medication condition, is then used for the corresponding simulations in the Section Modeling the BG Network in Healthy Control Subjects. The ON and the OFF medication status is brought out by Equation (2.4.3).

(2.4.3) $$\delta (t)=\{\begin{array}{ll} \left[a,b\right] & \;for\;controls\\ \left[a,\delta_{Lim}\right] & \;for\;PD\;OFF\\ \left[a,\delta_{Lim}+\delta_{Med}\right] & \;for\;PD\;ON \end{array}$$

where δ_*Lim*_ and δ_*Lim*_ + δ_*Med*_ are lesser than *b*.

Serotonin levels are also found to be lower in the PD patients (Fahn et al., 1971; Halliday et al., 1990; Bedard et al., 2011). The same is verified by the model parameters α_D1_, α_D2_, and α_D1D2_ in various medication cases of PD (Section Modeling the Reward-punishment Sensitivity in PD).

## Experiments and results

In this section, we apply the model of 5HT and DA in the BG (Section Modeling the BG Network in Healthy Control Subjects) to explain several reward/punishment/risk-based decision making phenomena pertaining to the BG function.

1. Simulating risk sensitivity (Long et al., 2009).
2. Simulating reward-punishment sensitivity (Cools et al., 2008).
3. Simulating reward-punishment sensitivity in Parkinson's Disease (Bodi et al., 2009).

In the simulation studies described in Sections Modeling the Risk Sensitivity to Modeling the Reward-punishment Sensitivity in PD, the BG model parameters [λ^GPi^—Equations (2.3.8, 2.3.9)] are set as shown in Table 3. The other parameters: gain functions (λ^Str^) of the D1R-, D2R-, D1R-D2R MSNs in the striatum equations (2.3.1, 2.2.3, 2.2.6); the model neuromodulator correlates for 5HT viz., α_D1_, α_D2_, α_D1D2_ that affect D1R, D2R, and the D1R-D2R MSNs, respectively; and DA parameters that condition PD (δ_Lim_, δ_Med_), are optimized for each experiment. The parameter values are initially selected using grid search and are eventually optimized using genetic algorithm (GA) (Goldberg, 1989) (Details of the GA option set are given in Supplementary Material A).

**Table 3:** **Parameters used in simulation studies of Sections Modeling the Risk Sensitivity to Modeling the Reward-punishment Sensitivity in PD Equations [2.3.8, 2.3.9]**.

	**λ^*GPi*^_*D*1_**	**λ^*GPi*^_*D*2_**	**λ^*GPi*^_*h*−*D*1_**	**λ^*GPi*^_*h*−*D*2_**
c_1_	1	1	0.05	0.05
c_2_	−50	50	−0.01	0.01
c_3_	0.01	0.01	−0.05	0.05

On studying the significance of 5HT modulation on different pools of MSNs, 5HT is found to significantly affect the D2R and the D1R-D2R co-expressing MSNs for explaining the experiments that deal with risk and punishment-based decision making (Cools et al., 2008; Bodi et al., 2009; Long et al., 2009) (Supplementary Material B). α_D1_ did not show much sensitivity to these experimental results. The results presented in the next section therefore equate α_D1_ = 1, and optimize α_D1D2_ and α_D2_ for every experimental condition (Refer to discussion section also).

### Modeling the risk sensitivity

#### Overview

In the study of Long et al. (2009), monkeys were presented with two choices of juice rewards, differing in the variances associated with the availability of the rewards (Long et al., 2009). One choice was associated with a risky reward and the other with that of a deterministic/safe one; these choices were of equal expected value (EEV) or unequal expected value (UEV) types. In EEV case both the safe and the risky choices to possess the same mean reward, while in UEV case mean rewards are unequal (Table 4). The monkey's risk sensitivity in the variable tryptophan conditions, viz., baseline (balanced) and Rapid tryptophan depleted (RTD), were recorded by analyzing their safe vs. risky reward selection ratio, under EEV and UEV cases.

**Table 4:** **The sample reward schedule adapted from Long et al. (2009)**.

**States, “***s***”**	**Safe target (ms)**	**Risky targets (ms)—each with probability 0.5**
	***(r^*j*^)***	
1	150	125,175
2	150	100,200
3	150	50,250
4	140	40,240
5	200	40,240
6	210	40,240

A non-linear risk sensitivity toward juice rewards was displayed by the monkeys: they exhibited risk-seeking behavior for small juice rewards and risk-aversive behavior for larger ones (Long et al., 2009). Furthermore, the experiment showed that when 5HT levels were reduced, the monkeys made more risky choices over the safer alternatives (Long et al., 2009), linking 5HT functioning to risk-based decision making. Therefore, this section analyses the property of risk sensitivity of the network model.

#### Simulation

The D1R, D2R and the D1R-D2R neuron weights are computed using Equation (2.3.1) and are updated using δ Equation (2.3.2). Learning rates are chosen as: η_D1_ = 0.3; η_D2_ = 0.1; η_D1D2_ = 0.1. The corticostriatal weights of D1R (*w*_*D*1_), D2R (*w*_*D*2_) and the D1R-D2R (*w*_*D*1*D*2_) MSNs are initialized randomly between 0 and 1; the value, risk and the utility functions are calculated using Equations (2.3.3–2.3.5). The parameters for the λ^Str^ in Equation (2.3.1) are provided in (Table 5).

**Table 5:** **Section Modeling the Risk Sensitivity: the parameters for Equations (2.3.1, 2.2.3, 2.2.6)**.

	**λ^*Str*^_*D*1_**	**λ^*Str*^_*D*2_**	**λ^*Str*^_*h*−*D*1_**	**λ^*Str*^_*h*−*D*2_**
c_1_	10	0.01	0.05	0.05
c_2_	−0.1	0.05	−5	0.5
c_3_	0	0	−100.1	100.1

This is done for all states “*s*” (tabulated in Table 4), and action sets consisting of “*a*” reaching the safe target and the risky target. The non-linearity in risk attitudes observed by the agent is accounted for by considering a reward base (*r*^*b*^) that is subtracted from the juice reward (*r*^*j*^) obtained. The resultant subjective reward *(r)* is treated as the actual immediate reward received by the agent Equation (3.1.1). Subtracting *r^*b*^* from *r^*j*^*, associates any *r*^*j*^ < *r*^*b*^ with an effect similar to losses, and any *r*^*j*^ > *r*^*b*^ with gains.

(3.1.1) $$r=r^{j}-r^{b}$$

The reward base (*r^*b*^*) optimized for the experiment is 159.83.

#### Results

**Figure 3:**
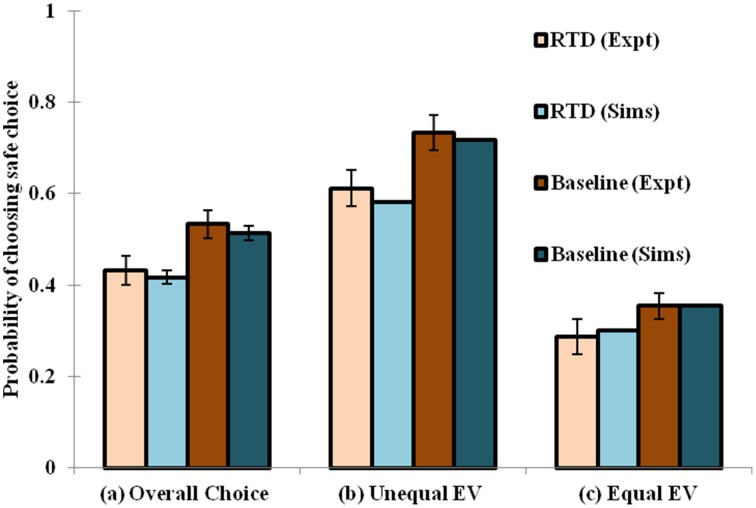
**Comparison between the experimental and simulated results for the (A) overall choice (B) Unequal EV (C) Equal EV, under Rapid Tryptophan Depletion (RTD) and Baseline (balanced) condition**. Error bars represent the Standard Error (SE) with size “*N*” = 100 (*N* = number of simulation instances). The experiment (Expt) and the simulation (Sims) results of any condition are not found to be significantly different. Here the experimental results are adapted from Long et al. (2009).

When the RTD condition is simulated by setting [α_D1_, α_D2_, α_D1D2_] = [1, 1, 0.0012], and the baseline by [α_D1_, α_D2_, α_D1D2_] = [1, 1, 1.32], a decrease in the selection of the safe choices is observed in the simulation as demonstrated in the experiment. The model has shown increased risk seeking behavior for low α condition particularly in the D1R-D2R co-expressing MSNs. Hence, modulating the α_D1D2_ best captures the baseline (high α_D1D2_) and RTD (low α_D1D2_) conditions for explaining risk sensitivity. The performance of the network model shown in this section is consistent with that of the lumped model described earlier (Balasubramani et al., 2014) in depicting the role of 5HT in risk-based action selection (Figure 3). More analysis on the effect of α_D1_, α_D2_, α_D1D2_ in showing risk sensitivity are provided in Supplementary Material B.

### Modeling punishment mediated behavioral inhibition

#### Overview

This section models an experiment showing differential variation in reward and punishment-based sensitivity in response to changing 5HT levels. In that experiment, the subjects underwent a reversal learning paradigm associated with deterministic rewards (Cools et al., 2008; Robinson et al., 2012). They were presented with two types of stimuli associated with reward and punishment, respectively. On each trial, the subject had to predict whether the stimulus presented to them would yield a reward or a punishment response, in a balanced or tryptophan depleted condition (Cools et al., 2008). The trials were grouped into blocks. Each subject performed 4 experimental blocks, that were preceded by a practice block in order to familiarize the subject with the task. Each experimental block consisted of an acquisition stage followed by a variable number of reversal stages. One of two possible experimental conditions was applied to each block: unexpected reward (punishment) condition where a stimulus previously associated with punishment (reward) becomes rewarding (punishing). Since there are 4 blocks of trials, there were two blocks for each condition. Performance of the subjects in the non-reversal trials was evaluated as a function of—(a) drink and condition (conditions: unexpected reward, unexpected punishment), and (b) drink and outcome (outcomes: reward, punishment) trial type. Results showed that performance did not vary significantly with condition in both balanced and tryptophan depleted cases. Errors were lesser for tryptophan depleted cases than balanced cases in both conditions. Specifically, errors decreased significantly for punishment-prediction trials compared to reward-prediction trials in tryptophan-depleted cases. Thus, the results suggest that tryptophan-depletion selectively enhances punishment-prediction relative to reward-prediction; and that 5HT maintains the behavioral inhibition (for active avoidance of the punishment). For a detailed explanation of the experimental setup refer to Cools et al. (2008).

#### Simulation

The two stimuli “*s*” (*s* ∈ {*s*_1_, *s*_2_}) are modeled as states, “*s*,” and the action, “*a*” (action *a* ∈ {*a*_1_ = *reward*, *a*_2_ = *punishment*}) associating the presented stimulus to a reward or punishment response. At any particular trial “*t*,” the rewarding association is coded by *r*_*t*_ = + 1, and the punitive association is coded by *r*_*t*_ = − 1. i.e., the outcome was stimulus-dependent and not response-dependent. The feedback of performance is given indirectly as followed in the experiment: erroneous trials are followed by the same stimulus until it is predicted by the agent correctly. The D1R, D2R, and the D1R-D2R neuron weights are trained using Equation (2.3.1) where δ is from Equation (2.3.2). The learning rates are: η_D1_ = η_D2_ = η_D1D2_ = 0.01. The weights of the D1R, D2R, and the D1R-D2R neurons are initialized randomly between 0 and 1; the value, risk and the utility functions are calculated using Equations (2.3.3–2.3.5). The parameters used for λ^*Str*^ in Equation (2.3.1) are as in (Table 6).

**Table 6:** **Section Modeling Punishment Mediated Behavioral Inhibition: parameters for λ used in Equations (2.3.1, 2.2.3, 2.2.6)**.

	**λ^***Str***^_***D*1**_**	**λ^***Str***^_***D*2**_**	**λ^***Str***^_***h*−*D*1**_**	**λ^***Str***^_***h*−*D*2**_**
c_1_	0.06	0.115	0.939	0.939
c_2_	−0.155	0.488	−0.188	0.188
c_3_	−0.574	0.317	−1.723	1.723

Similar, to the experiment, three types of trials are simulated as follows: non-reversal trials in which the association of a stimulus–response pair is learnt; reversal trials in which the change of the learnt association is triggered; and the switch trials where the reversed associations are tested. The maximum number of reversal stages per experimental block is 16, with each stage to continue till the correct responses fall in the range of (5–9). The block terminates automatically after 120 trials. There are two blocks in each condition, and hence a total of 480 trials (4 blocks) conducted per agent. The design of the experiment has an inbuilt complementarity in the association of the actions to a particular stimulus (i.e., increasing the action value of *a*_1_ for a stimulus, *s*, decreases the same for *a*_2_ to *s*), and the stimuli to a particular action (i.e., increasing the action value of *a* to *s*_1_ decreases the same for *a* to *s*_2_). Hence in the simulations, the action values associated with the two actions (*Q*(*s*, *a*_1_) and *Q*(*s*, *a*_2_)) for any particular state “s” are simulated to be complimentary Equation (3.2.1) at any trial “*t.*”

(3.2.1) $$w_{D1}(s,a_{1})=-w_{D1}(s,a_{2})$$

The action values of the two stimuli “*s*” (*Q*(*s*_1_, *a*) and *Q*(*s*_2_, *a*)) mapped to the same action, “*a*” are also complimentary Equation (3.2.2) at any trial “*t.*”

(3.2.2) $$w_{D1}(s_{1},a)=-w_{D1}(s_{2},a)$$

Hence, only one out of the four value functions (Q(s_1_, a_1_), Q(s_1_, a_2_), Q(s_2_, a_1_), Q(s_2_, a_2_)) or their corresponding weights is learnt by training, while the other 3 are set by the complementarity rules to capture the experimental design. We assume that, in the experiment, such a complementarity could be learnt during the initial practice block that promoted familiarity.

#### Results

**Figure 4:**
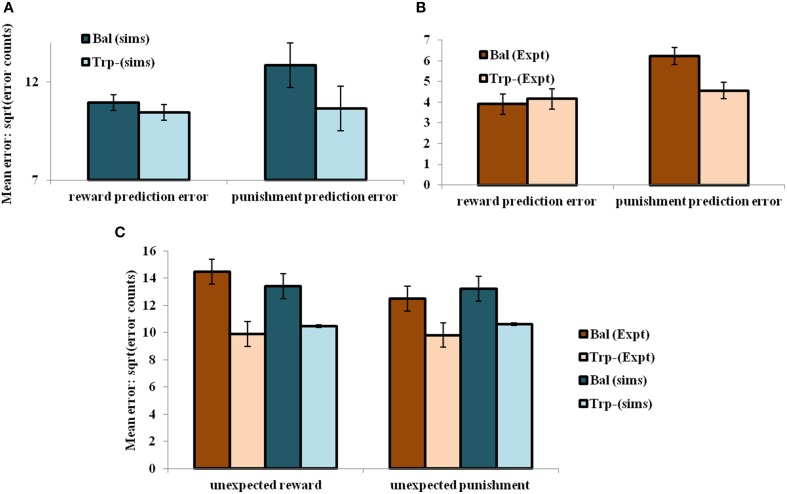
**The mean number of errors in non-switch trials (A) as a function of “α” and outcome trial type; Error bars represent standard errors of the difference as a function of “α” in simulation for size “*N*” = 100 (*N* = number of simulation instances) (Sims)**. **(B)** Experimental error percentages adapted from Cools et al. (2008). Error bars represent standard errors as a function of drink in experiment (Expt). The results in **(B)** were reported after the exclusion of the trials from the acquisition stage of each block. **(C)** The mean number of errors in non-switch trials as a function of condition with experimental (Expt) results adapted from Cools et al. (2008). Error bars represent standard errors either as a function of drink in experiment (or α) in simulation for size “*N*” = 100, with bal and Trp- representing balanced and tryptophan depleted conditions, respectively. The experiment (Expt) and the simulation (Sims) results of any condition or outcome trial type are not found to be significantly different.

On analyzing the results in terms of experimental condition (viz., unexpected reward and unexpected punishment valences), the overall error decreased on the reduction of 5HT (α) level [α_D1_, α_D2_, α_D1D2_] = [1, 2.25, 1] (tryptophan-depleted condition) from [α_D1_, α_D2_, α_D1D2_] = [1, 5, 1] (balanced condition) (Figure 4C). Particularly 5HT modulation on the D2R MSN is predicted to control the increased punishment prediction observed during the depleted tryptophan conditions. The punishment prediction error decreased significantly more than the reward prediction error (Figure 4B) on the reduced α_D2_ condition. Hence α_D2_ in our model best represents 5HT's role in selectively modulating punishment sensitivity (Figure 4).

Increased 5HT levels in balanced condition are seen promoting the inhibition of responses to punishing outcomes (Figure 4A) as proposed by Cools et al. (2008) (Figure 4B). Reducing 5HT via tryptophan depletion then removes this inhibition. The *sign*() term in the Equation (2.3.5) is essential in showing the non-linear reward-punishment sensitivity, as observed in our earlier study (Balasubramani et al., 2014). The errors as a function of conditions i.e., in unexpectedly rewarding and punitive trials, are obtained to be the same in both the balanced and tryptophan depleted cases (Figure 4C: sims values) again matching with the experiment (Figure 4C: expt values adapted from Cools et al., 2008).

### Modeling the reward-punishment sensitivity in PD

#### Overview

**Figure 5:**
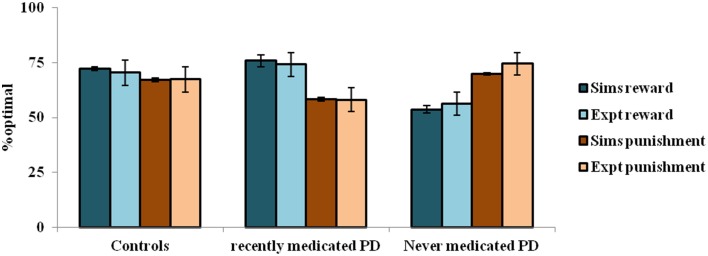
**The reward punishment sensitivity obtained by simulated (Sims)- PD and controls model to explain the experiment (Expt) of Bodi et al. (2009)**. Error bars represent the standard error (SE) with *N* = 100 (*N* = number of simulation instances). The Sims matches the Expt value distribution closely, and are not found to be significantly different.

The simulation studies presented so far are performed under controlled conditions. This section simulates a study related to reward-punishment learning that involved PD patients. Bodi et al. (2009) used a probabilistic classification task for assessing reward-punishment learning under the different medication conditions of PD patients. The medications used in the study were a mix of DA agonists (Pramipexole and Ropinirole) and L-Dopa. The task was as follows: one of four random fractal images (I1–I4) were presented. In response to each image, the subject had to press on one of two buttons—A or B–on a keypad. Stimuli I1 and I2 was always associated with reward (+25 points), while I3, I4 was associated with loss/punishment (−25 points). The probability of reward or punishment outcome depended on the button (A or B) that the subject pressed in response to viewing an image. The reward/punishment probabilities associated with two responses, for each of the four stimuli, are summarized in Table 7. There are 160 trials administered in 4 blocks. Experiments were performed on controls, never-medicated (PD-OFF) and recently-medicated PD (PD-ON) patients. The study (Bodi et al., 2009) showed that the never-medicated patients were more sensitive to punishment than the recently-medicated patients and controls. On the other hand, the recently-medicated patients outperformed the never-medicated patients and controls on reward learning tasks (Figure 5). The optimal decision (as shown in the Figure 5) is the selection of A for I1 and I3, and B for I2 and I4.

**Table 7:** **The four types of images (I1–I4) associated with response type A and B with the following probability are presented to the agent, and the optimality in sensing the reward (right associations) and the punishment (incorrect associations) are tested in control and PD condition**.

**Learning**	**Reward**		**Punishment**	
Image presented	I1	I2	I3	I4
Optimal type	A	B	A	B
Probability(points)	0.8(+25)	0.8(+25)	0.8(0)	0.8 (0)
for optimal type	0.2(0)	0.2(0)	0.2(−25)	0.2 (−25)
Non-optimal type	B	A	B	A
Probability(points)	0.2(+25)	0.2(+25)	0.2(0)	0.2(0)
for non-optimal type	0.8(0)	0.8(0)	0.8 (−25)	0.8(−25)

#### Simulation

The immediate reward condition of the experiment is expressed by Equation (2.3.2), with which the weights of value (D1R) update and the risk (D1R-D2R) update Equation (2.3.1) are made for every (state-action) pair. The states here are the 4 images and the action, *a*, is categorizing them as A or B. The utility for a particular (state-action) pair is constructed using Equation (2.3.5). On presentation of an image, the change the utility associated with it Equation (2.3.6) is used for the action selection which is defined by dynamics described in Sections Modeling the BG Network in Healthy Control Subjects. It must be noted that the +25 reward is parameterized as reward “*r* = 1” and the -25 punishment as “*r* = −1.” The weights for the D1R, D2R, and the D1R-D2R neurons are initialized randomly between 0 and 1. The parameters used for the λ^*Str*^ in Equation (2.3.1) are as in (Table 8). The modeling of the PD-ON (on dopamine agonists medication), and PD OFF (OFF dopamine agonists medication) are as Equation (2.4.3); and step sizes set are η_D1_ = 0.01; η_D2_ = 0.1; η_D1D2_ = 0.1;

**Table 8:** **Section Modeling the Reward-punishment Sensitivity in PD: Parameters used for the λ in Equations (2.3.1, 2.2.3, 2.2.6)**.

	**λ^*Str*^_*D*1_**	**λ^*Str*^_*D*2_**	**λ^*Str*^_*h*−*D*1_**	**λ^*Str*^_*h*−*D*2_**
c_1_	1	1	0.05	0.05
c_2_	−50	50	−0.01	0.01
c_3_	0	−1	−0.05	0.05

#### Results

In the experiment, the controls show almost equal sensitivity to rewards and punishments. The PD ON patients show an increased sensitivity to reward compared to that of punishment, whereas the PD OFF patients show the opposite trend. The parameters of the model that best represent the experiment are: [α_D1_, α_D2_, α_D1D2_] = [1, 1, 0.2] for the healthy controls; [δ_Lim_, α_D1_, α_D2_, α_D1D2_] = [0.001, 1, 0.99, 0.001] for PD-OFF; and [δ_Lim_, δ_Med_, α_D1_, α_D2_, α_D1D2_] = [0.001, 0.021, 1, 0.2, 0.001] for PD-ON.

The depleted DA levels limit the update [through the Equation (2.3.2)] of the cortico-striatal connections. The resulting erroneous value and the risk components would interfere with the reward-punishment sensitivity of the PD patients. Particularly, the exact nature of the impairment is shown to be different under cases of ON and OFF DA medications. In PD-ON, DA-agonist medication tends to increase the tonic levels of DA (Frank et al., 2007b). This leads to faulty updates of the states associated with punishment, which must be ideally associated with a low “value.” This also increases the risk component associated with those states to eventually decrease their selection optimality. The opposite trend occurs in PD-OFF condition which decreases the optimality in selection associated with the states scoring rewards. Moreover, the results substantiate both the differential modulation of 5HT in the MSNs and their changes marking PD (Figure 5). Modulating 5HT along with DA is essential for representing PD OFF and ON medication conditions (Supplementary Material B) as identified in experimental studies (Fahn et al., 1971; Halliday et al., 1990; Tan et al., 1996; Bedard et al., 2011). Specifically, a lowered α_D1D2_ is seen in both OFF and ON medication cases, while a lowered α_D2_ is seen in the PD-ON case.

Supplementary Material C is added to demonstrate the relative influence of *sign*() term on the reward, punishment sensitivity under various conditions (controls, PD-ON, PD-OFF). Supplementary Materials B,C also predicts the significance of treating PD patients with 5HT (α_D1_, α_D2_, α_D1D2_) + DA medication (δ_Lim_, δ_Med_) for improving their reward and punishment learning. The non-linearity in the utility formulation due to the *sign*() term is also found to be essential for capturing the increased punishment sensitivity in PD-OFF case, and an increased reward sensitivity in PD-ON case (Supplementary Material C).

## Discussion

### The DA-5HT based BG network model for utility based decision making

The model presented in Section Cellular Correlates for the Value and the Risk Computation is an abstract mathematical model (and not a network model). It aims to explain the results from behavioral experiments that embody the diversity of existing theories of serotonin in the BG. In classical Actor-Critic approaches to modeling the BG function, value computation is thought to occur in the striatum (Joel et al., 2002). There is evidence from functional imaging that supports this theory (O'Doherty et al., 2006). In the present study, we seek to replace the value function with the more general utility function, so as to include the neuromodulatory actions of 5HT in addition to DA. Ideally, a convincing model of value computation in the striatum must go beyond an abstract lumped representation and demonstrate how value may be computed by neural substrates of the striatum. There is strong evidence for the existence of dopamine-modulated plasticity in corticostriatal connections, an effect that is necessary to account for value computation in the MSNs of the striatum (See review by Kötter and Wickens, 1998). The idea that MSNs are probably cellular substrates for value computation has found its place in recent modeling literature (Morita et al., 2012). Starting from the fact that the effect of dopamine on the D1R—expressing MSNs of the striatum is to increase the firing rate, it has been shown in a computational model of the BG that the D1R-expressing MSNs are capable of computing value (Krishnan et al., 2011). We then extend this idea and show that a model of D1R-D2R co-expressing MSNs in the striatum is capable of computing the risk function in Section Cellular Correlates for the Value and the Risk Computation.

The present study presented a model of co-expressing D1R-D2R MSNs' gain function as an addition of the gain functions of D1R and the D2R MSNs. As a result the D1D2R MSNs acquire a “U”-shaped gain function. A few experiments provide support for such a representation, for instance the study by Allen et al. (2011) on neurons coexpressing D1-like and D2-like receptors in *C. elegans* (Allen et al., 2011). Here the D1R and D2R of a co-expressing neuron have antagonistic effects on neurotransmittor (acetylcholine) release. In conclusion, they propose that the D1R-D2R coexpressing neurons could simply be a combination of D1R and D2R neurons. Even studies on rodents and *in-vitro* striatal cultures have shown the antagonistic nature of the D1 and the D2 receptor components of a co-expressing neuron (Hasbi et al., 2011). They report that these co-expressing neurons activate the CAMKII and BDNF machinery, each of which is known to play opposing roles in synaptic plasticity—long term potentiation and long term depression, which are generally agreed to be dependent on the D1R and the D2R, respectively (Surmeier et al., 2007). We follow such a perspective of simple addition of the antagonistic D1 and the D2 neuronal gain functions to model the D1R-D2R MSN in our modeling study.

Few studies in the BG show the ventral striatal neurons to be specially involved in risk processing (Stopper and Floresco, 2011). In this regard, we further hypothesize that D1R-D2R MSNs in those nuclei (Stopper and Floresco, 2011) would specifically contribute to risk computation observed in Stopper and Floresco (2011). We also predict that selective loss of these co-expressing neurons would make the subject less sensitive to risk, and therefore show risk-seeking behavior. The next part of the model (Section Modeling the BG Network in Healthy Control Subjects) deals with realizing action selection through network dynamics of the BG. The underlying stochasticity in the soft-max rule used in our early study (Balasubramani et al., 2014) is achieved indirectly by the chaotic dynamics of the STN-GPe loop (Kalva et al., 2012). A schematic of the network model is presented in Figure 2.

#### Improvements over the abstract model

This study involves a systematic expansion of the lumped model proposed earlier (Balasubramani et al., 2014) to a complete network model of the BG that describes the interactions between DA and 5HT in action selection dynamics. Though it has a shortcoming that it does not include the detailed elaboration of DA-5HT interactions in the various kinds of receptors in the BG, it reconciles the principal network theories with the cellular machinery in the BG for modeling the behavioral results listed in the experiments of Section Experiments and Results.

Furthermore, the previous abstract model is primarily a model of the striatum. It focuses on the utility function, which is thought to be computed in the striatum, and its role in decision making. The actual decision making is done using softmax function applied to the utility function (Section A Model of Utility-based Decision Making). But the present study attempts to model the entire basal ganglia. It includes downstream structures like GPe, STN, and GPi. Decision making occurs in GPi and thalamus. Thus, softmax-like stochastic decision making is implemented in the present model by the chaotic activity of STN-GPe oscillations and the competitive action selection in the GPi and thalamic modules (Section Modeling the BG Network in Healthy Control Subjects). The δ_*U*_ plays a role in determining the competition/cooperation between the direct and indirect pathways, a mechanism that could not have been accommodated in the previous abstract model.

There exists a model of risk based on an “asymmetric learning rule” that works by multiplying a risk sensitivity factor with the temporal difference function, without explicitly representing the “risk” component (Mihatsch and Neuneier, 2002). This study follows the idea of utility computation with explicit risk coding, as reported in various studies (Preuschoff et al., 2006; Brown and Braver, 2007; Christopoulos et al., 2009; D'Acremont et al., 2009), for modeling the utility computation in the BG.

#### The co-expressing D1R-D2R MSNs

There have been varied reports of the proportion of co-expressing D1R-D2R MSNs in the striatum. These neurons were not modeled in any of the earlier studies (Frank et al., 2004; Ashby et al., 2010; Humphries and Prescott, 2010; Krishnan et al., 2011). Such unacknowledged nature of the D1R-D2R MSNs in the striatum might be due to the following: The existence of co-expressing D1R-D2R MSNs has been debated for years. Many studies supported distinct populations of the striatal MSNs projecting in striatonigral and striatopallidal pathways including neurochemical and genetic ontology analysis in mice (Araki et al., 2007), transgenic mice engineered using Bacterial artificial chromosome with enhanced green fluorescent protein (Bertler and Rosengren, 1966; Shuen et al., 2008; Matamales et al., 2009; Valjent et al., 2009), biochemical and imaging assays including *in situ* hybridization (ISH) combined with retrograde axonal tracing (Gerfen et al., 1990; Le Moine et al., 1991; Le Moine and Bloch, 1995), fluorescence-activated cell sorting (FACS) of MSNs or translating ribosome affinity purification approach (TRAP) (Lobo et al., 2006; Heiman et al., 2008). These studies report that D1Rs are present in striatonigral MSNs and are Substance P positive, whereas the D2R are enriched with enkephalin and are striatopallidal in nature (Classical models of the BG: Albin et al., 1989; Delong, 1990). However, some of these highly sensitive studies are under debate due to the following reasons (Bertran-Gonzalez et al., 2010; Calabresi et al., 2014). The developmental regulation of D1R and D2R mRNAs as analyzed in the genetic ontology studies with mice (Araki et al., 2007) would result from intrinsic genetic programs that control the receptors' expression, whereas the actual dopaminergic neuron's innervations in a projection area (here, the striatum) is studied to control the D1R and D2R expression (Jung and Bennett, 1996). Furthermore, the genetically engineered BAC mice show differences from wild-type mice in terms of behavioral, electrophysiological and molecular characterization. Experimental support for the segregation of the pathways offered by even highly advanced optogenetics and other imaging techniques is questioned for their ability to monitor subcortical activity accurately in the behaving animals (See the reviews by Bertran-Gonzalez et al., 2010; Calabresi et al., 2014).

Meanwhile, there are many other findings questioning the strict segregation of the direct and the indirect pathways. See review by Bertran-Gonzalez et al. (2010), Calabresi et al. (2014) for more details. These studies report various modes of cross-talk existing between the “classical” dichotomous projections from the striatum. Studies also report co-expression of the D1R and the D2R in a MSN to be a medium for cross-talk. They even propose the receptors' heteromerization to such an extent that these co-expressing MSNs would have their downstream effects completely different from that of the neurons solely expressing the D1R or the D2R. The studies reporting co-expression of D1R-D2R in the MSNs analyze components such as calcium and BDNF (Brain-derived neurotrophic factor) (Rashid et al., 2007; Hasbi et al., 2009), using techniques such as RT-PCR (Reverse transcription polymerase chain reaction) that is reviewed in Surmeier and Kitai (1993), Surmeier et al. (1996), co-immunoprecipitation (Lee et al., 2004), or FRET (Fluorescence resonance energy transfer) using fluorophore-labeled antibodies (Hasbi et al., 2009). Some quantitative measures regarding the proportion of D1R-D2R MSNs in the striatum include nearly 17% in the nucleus accumbens- shell, and 6% in the caudate-putamen, when estimated using BAC transgenic mice (Bertran-Gonzalez et al., 2008). Though there have been doubts regarding the accurate neuronal labeling in BAC transgenic mice, the proportions have been confirmed by the later studies too (Matamales et al., 2009). A recent study employing confocal FRET analysis also confirmed the colocalised D1R-D2R in the striatum (Hasbi et al., 2009; Perreault et al., 2010). Hence these studies favor the presence of D1R-D2R MSNs in significant levels in the striatum.

A few studies report the projection of D1R-D2R co-expressing neurons to GPi also (Perreault et al., 2010, 2011). Though our present study accounts for their projection to GPe alone, out of this study comes a strong suggestion that the D1R-D2R co-expressing neurons targeting the pallidum would mainly contribute to risk computation as in Equation (2.3.9). Those D1R-D2R MSNs that project to SNc may be utilized for generating temporal difference in utility computation Equation (2.3.6). These projections of the D1R-D2R co-expressing neurons toward both the indirect pathway and the direct pathway, support the study that DA D1R containing neurons may not solely project onto the direct pathway. This is because some of the D1R containing MSNs are known to also project to the indirect pathway (Calabresi et al., 2014). Those D1R neurons could be co-expressing D2R, since D1R-D2R co-expressing MSNs are capable of invading both the direct and the indirect pathways (Nadjar et al., 2006; Bertran-Gonzalez et al., 2010; Hasbi et al., 2010, 2011; Perreault et al., 2010; Calabresi et al., 2014). Similarly the D2R MSN need not just solely project to the indirect pathway. The study of Calabresi et al. (2014) shows that D1R-D2R MSNs are one of the means by which the direct and the indirect pathways interact. Such a notion is preserved in our modeling study too, and hence these D1R-D2R co-expressing MSNs might play a major role in the cross-talk between the direct and the indirect pathways.

Moreover, DA D1R and D2R are also shown to form heteromeric complexes with unique functional properties and phenotype (Hasbi et al., 2011; Perreault et al., 2012). These heteromers are found to have increased sensitivity following repeated increases in DA transmission. The up-regulated state of these heteromers persisted after DA agonist removal, identifying these heteromeric complexes as therapeutic targets in DA-related disorders, such as schizophrenia and drug addiction. These heteromers are also predicted to significantly influence cognition, learning, and memory (Perreault et al., 2011, 2012). We would expect that there might be differences between the co-expressing neurons and the heteromers, but in the absence of more data, this study has used the simple model of addition of D1R and D2R MSN's gain functions to represent the D1R-D2R co-expressing neurons.

#### Striatal DA and 5HT

The DA signals used in our model are a function of reward/value, and temporal difference in value/utility (Figure 2, Table 2). The existence of different forms could be possible because:

1. Distinct sets of dopamine neurons are known to project to striatum. For instance structures such as the striosome and matrisome are proposed to receive different DA modulatory signals (See the Section “Modularity of dopamine signals” in Amemori et al., 2011). Some studies found that though all the SNc DA neurons innervate both the striosomes and matrisomes, there is a bias at the level of individual neurons (Matsuda et al., 2009).
2. Similarly dopaminergic neurons from different regions dorsal/ventral of SNc/VTA might represent different computational quantities (See Section “Modularity of dopamine signals” in Amemori et al., 2011).
3. Moreover certain DAergic signals are known to specifically modulate between trials, while some other are proposed to act like a teaching signal within a trial (Tai et al., 2012; Stauffer et al., 2014).
  A review by Schultz (2013) along with other studies (Lak et al., 2014; Stauffer et al., 2014) state that the dopamine neurons are known to reflect various reward attributes such as the magnitude, probability and delay. In fact the above-mentioned attributes also get reflected when dopamine neurons can inform the first derivative of value or the utility function, as a common neuronal implementation (Stauffer et al., 2014).
4. Our model proposes that the δ and *sign*(*Q*) (Figure 2, Table 2) affect the computation of utility function by the MSNs. It must be noted that δ affects all the three kinds of MSNs (D1R, D2R, and the D1R-D2R MSNs) pre-synaptically as investigated through many experimental studies (Refer, Kötter and Wickens, 1998; Reynolds and Wickens, 2002). But the *sign*(Q) correlate of DA is proposed to affect the responses of D1R-D2R MSNs.

Whereas, the neuromodulator 5HT is predicted to significantly modulate the D2R and the D1R-D2R co-expressing neurons (refer Supplementary Material B for the simulations). The receptors 5HT 1, 2A, 2C and 6 (Ward and Dorsa, 1996; Di Matteo et al., 2008b) are most abundantly expressed in the striatum. None of these receptors show preferential co-localisation to any striatal proteins, such as substance P, dynorphin (neurons that contribute to the striato-nigral direct pathway) or enkephalin (contributing to the indirect pathway). But a differential expression indeed exists—5HT2C is highly expressed in the patches, and 5HT2A in the matrix (Eberle-Wang et al., 1997). These 5HT receptors are more likely to be co-expressed even along with the D1R-D2R MSNs which form a substantial portion of the striatum according to certain experimental studies (Nadjar et al., 2006; Bertran-Gonzalez et al., 2010; Hasbi et al., 2010, 2011; Perreault et al., 2010; Calabresi et al., 2014). It is true that 5HT's specificity in expression along with a particular type of MSN is still not clear.

In order to investigate the possibility that 5HT modulation of MSNs may not be limited only to D1R-D2R MSNs, but could have a differential action on the three pools of MSNs (D1R, D2R, and D1R-D2R), we have conducted additional simulations and obtained quite revealing results (Supplementary Material B). On varying different subsets of {α_*D*1_ *eqn*. 2.3.8)α_*D*2_, *and* α_*D*1*D*2_ *eqn*. 2.3.9)}, the following inferences are made:

1. The modulation of α_D1_ alone [α_D2_ = 1, α_D1D2_ = 1] is not able to consistently model the behavior of a balanced (high α_D1_) or the reduced tryptophan (low α_D1_) conditions in any experiment. Similar is the case of modulating α_D2_ [α_D1_ = 1, α_D1D2_ = 1] alone.
2. The joint modulation of α_D1_ and α_D2_ [α_D1D2_ = 1] was not able to explain any of the experiments satisfactorily.
3. α_D1D2_ is found to be able to explain the results of the experiment by Cools et al. (2008) better only when optimized along with α_D2_. The joint modulation of α_D2_ and α_D1D2_ [α_D1_ = 1] achieves best fit for all the experiments.
4. α_D1_ is not found to be as sensitive as α_D1D2_ and α_D2_ in all the experiments, though a non-zero α_D1_ is preferred.
5. In summary, α_D1_ representation of 5HT can be fixed at 1, while the others α_D1D2_ and α_D2_ can be varied and optimized to explain different 5HT based experimental results.

The optimization of fixed 5HT values might also be related to the tonic modulation exerted by DRN during reward processing (Jiang et al., 1990; Alex and Pehek, 2007; Nakamura, 2013).

Such a framework is shown to effectively relate to the lumped model of the BG (Balasubramani et al., 2014) by explaining the experiments analyzing risk, reward, and punishment sensitivity. Especially the roles of DA-5HT in risk sensitivity, time scale of reward prediction and punishment sensitivity/behavioral inhibition are reconciled using a value and risk based decision making framework. Thereby the test beds include experiments to analyse the behavioral parameters such as DA and 5HT for risk (Long et al., 2009), punishment sensitivity and behavioral inhibition (Cools et al., 2008) and probabilistic reward-punishment sensitivity (Bodi et al., 2009).

One other property of 5HT is coding for the time scale of reward prediction. This was verified in our earlier study (Balasubramani et al., 2014) by correlating 5HT parameter α_D1D2_ that is modulating the D1R-D2R MSNs to the time discount factor γ as in Equation (2.1.3). Risk sensitivity has also been correlated to the reward delays by various other experimental studies (Hayden and Platt, 2007; Kalenscher, 2007). These studies predict that primates make risky choices when rewarded probabilistically with shorter delays, and they become risk aversive on increasing the waiting period for observing the probabilistic rewards, again substantiating our earlier lumped model relating α_D1D2_ to γ. Since this paper focuses on realizing our earlier empirical study at the network level, we focus only on the experiments affecting the network attributes such as risk coding D1R-D2R MSNs (in Section Modeling the Reward-punishment Sensitivity in PD), and the non-linear risk sensitivity (in Section Modeling Punishment Mediated Behavioral Inhibition).

Note that the proposed model brings the analysis of the reward-punishment sensitivity into a risk-based decision making framework, but there exist some tasks that deterministically test for the reward-punishment sensitivity. The D2 MSNs are known to mediate the No-Go effect that predominates in a reflexive behavioral inhibition in the face of expected punishment (loss function) alone, that is, free of risk (Frank et al., 2004; Nambu, 2004, 2008; Chakravarthy et al., 2010). This study also shows the importance of 5HT in modulating the D2 MSNs, for explaining the property of behavioral inhibition (ref: Supplementary Material B) in Cools et al. (2008) in the face of expected punishment.

In summary, the proposed network model of the BG associates the three pools of striatal MSNs—with D1R, D2R, and co-expressing D1R-D2R to three different sensitivities—reward, punishment and risk, controlling decision-making activity, respectively.

#### The DA-5HT interactions

Serotonin does not monopolize in controlling risk and punishment sensitivity. Besides having a role in reward prediction, DA in the midbrain is proposed to represent the risk component of the environment (Schultz, 2010a), and DA levels in the frontal cortex are known to rise in response to inescapable punitive stimuli, establishing a collaborative effect with 5HT. The collaborative and the opposing effects of DA and 5HT at the behavioral level are also seen at the cellular and receptor level (Di Matteo et al., 2008a,b). Increased meso-striatal DA levels on the blockade of the central 5HT2C receptors, is an instance of the opposing effect; while collaborative responses like an increased antipsychotic effect by combining the blocking of 5HT2A and D2 receptors, moreover an antidepressant effect is seen on boosting either 5HT or DA; whereas cases of neither collaborative or opposing effects are observed on responding to inescapable punishment conditions and aversive learning (Cools et al., 2010; Boureau and Dayan, 2011). Complex interactions exist between DA and 5HT making it difficult to tease apart precisely the relative roles of the two molecules in reward evaluation. Even at the neuromodulator releasing sites, some subtypes of 5HT receptors facilitate DA release, while others (like 5HT2C) inhibit them (Alex and Pehek, 2007). In summary, it is clear that the relationship between DA and 5HT is not one of simple complementarity—both synergistic and opposing interactions exist between these two neuromodulators in the brain (Boureau and Dayan, 2011).

Though this study does not specifically model DA and 5HT interactions at any particular BG region, the *sign*(*Q*) term in the utility formulation Equation (2.3.9) may be regarded as a reflection of complex interactions between DA and 5HT in modeling terms. This is because the *sign(Q)* term gets multiplied with the α_D1D2_ (5HT) term and the D1R-D2R co-expressing MSN output to eventually represent the “risk component.” A more detailed network model of the BG, in which the striatum is divided into striosomes and matrisomes (Amemori et al., 2011), is currently being developed by our group. The striosomes are modeled to constitute the D1R and the D1R-D2R co-expressing MSNs that target DA releasing SNc. The SNc neurons, which receive the information about the value and the risk function from the D1R and the D1R-D2R co-expressing MSNs, release their DA to the striatal matrisomes (Jakab et al., 1996; Surmeier et al., 1996; Nadjar et al., 2006; Amemori et al., 2011; Calabresi et al., 2014). Hence DA could be a potential source of interaction among the striosomes and matrisomes, which is also roughly captured by a DA form [: = *sign*(*Q*) term in the Equations (2.1.7, 2.3.5, 2.3.9), and Figure 2]. Such a value function like response of DA neurons have been reported earlier by experimental studies (Schultz, 2010b). The matrisomes contain the D1R MSNs projecting over the DP, and the other MSNs (D2, D1R-D2R) projecting over the IP. The selection of a striosome appropriately activates the corresponding matrisomes for action selection dynamics.

#### Study outcomes on reinforcer-sensitivity in controls and parkinson's disease

The key study outcomes include the following:

- The action of DA in the BG is proposed to be of different forms [δ in Equation (2.3.2), δ_U_ in Equation (2.3.6), and *sign*(*Q*) in Equations (2.3.5, 2.3.9)] as summarized in Figure 2.
- The DA-5HT joint action on D1R MSNs and the D1R-D2R coexpressing MSNs makes them suitable as cellular substrates for value and risk function computations, respectively.
- The modulation of 5HT (α_D1_) on D1R MSN is not found to be particularly sensitive for explaining the experimental tasks described in Section Experiments and Results (Supplementary Material B).

##### Risk sensitivity in controls:

- The modulation of 5HT (α_D1D2_) on *D1R-D2R co-expressing MSN* is found to be significant (Section Modeling the Risk Sensitivity, Supplementary Material B) for explaining *risk-sensitivity* (Long et al., 2009).
- The simulation results with decreased model 5HT levels are shown to effectively explain the increased risk seeking behavior shown in Long et al. (2009) experimental study.

##### Punishment sensitivity in controls:

- The modulation of 5HT (α_D2_) on the *D2R MSN* is found to be important (Section Modeling Punishment Mediated Behavioral Inhibition, Supplementary Material B) for explaining the behavioral inhibition and punishment-sensitivity (Cools et al., 2008).
- Balanced condition of the model with high 5HT levels is shown to be facilitating behavioral inhibition in comparison to Tryptophan depleted condition (reduced 5HT levels) as proposed by Cools et al. (2008) experimental study.

##### Reinforcer sensitivity in Parkinson's Disease:

- A model (Section Modeling the Reward-punishment Sensitivity in PD) of limited DA availability simulates the PD-OFF, while an added medication factor to the limited DA marks the PD-ON. Differential modulation of 5HT in the D1R-D2R MSNs with α_D1D2_ = 0.2 (in controls) and α_D1D2_ < 0.2 (in PD) explain the increased reward optimality in PD-ON and increased punishment optimality in PD-OFF condition reported in experimental studies (Bodi et al., 2009).
- The activity of 5HT in the D2R MSNs is significantly lowered specifically in the PD-ON condition (PD-ON α_D2_ = 0.2 compared to α_D2_ > 0.2 in PD-OFF and controls). Many neurobiological experimental studies have observed lowered 5HT levels in PD compared to the controls (Fahn et al., 1971; Halliday et al., 1990; Bedard et al., 2011). This is captured in our modeling study (Section Modeling the Reward-punishment Sensitivity in PD) with a smaller α value observed to modulate both the D2R and the D1R-D2R MSNs.
- The PD-ON condition is reported to have lowered 5HT levels than the OFF medicated PD condition. This is shown by reduced 5HT release, and increased DA release from the serotonergic neurons in the presence of L-Dopa (Tan et al., 1996; Reed et al., 2012). This is specifically reflected by a significant decrease in the level of α_D2_ affecting the D2R MSNs of our modeling study (Section Modeling the Reward-punishment Sensitivity in PD).

#### Predictions and future work

The 5HT correlate of the model is a parameter denoting the *tonic* serotonergic activity. Many experimental recordings show tonic activity as the prevalent form of serotonergic action (Aghajanian et al., 1978; Vandermaelen and Aghajanian, 1983). Though there are some computational models on phasic serotonergic activity (Daw et al., 2002), its biological existence and relevance is still dubious (Boureau and Dayan, 2011; Cools et al., 2011; Dayan and Huys, 2015). We look forward to study more about the tonic and phasic forms of serotonergic activity in the future. Further, investigation should examine more detailed DA-5HT interactions based on the specific receptor type distribution in the BG. This study only deals with the theoretical principles behind DA-5HT interactions in the BG, which can be then expanded to understand the detailed influence of the same interactions in the cortex, SNc, and Raphe nucleus. Apart from analyzing the details of the interactions in various regions of the brain, attempts to include other major neuromodulators like acetylcholine (Ach) and norepinephrine (NE) are also desired. This could be realized by including a self-organized map (SOM) model of the striatum which captures its topologically ordered arrangement of the striosomes and matrisomes (Stringer et al., 2002) and is controlled by the Ach mediated tonically active inter-neurons. The model would help to analyse Ach influence in the selection of striosome–matrisome pairs and the plasticity of cortico-striatal connections (Spehlmann and Stahl, 1976; Ding et al., 2011). Specific investigation of how the neuromodulator NE affects the STN-GPe system and the BG dynamics is also of special interest. Neuromodulator NE has been compared to the inverse temperature parameter of Equation (2.1.8) and is thought to specifically affect the exploration dynamics of the BG action selection machinery (Doya, 2002; Aston-Jones and Cohen, 2005). In our earlier study, we have showed that the STN lateral connections can also influence the BG exploration dynamics significantly (Chakravarthy and Balasubramani, 2014). The impact of DA and NE activity on STN functioning should be tested in future, paving way to a comprehensive computational understanding of the roles of all the four major neuromodulators (DA, 5HT, NE, Ach) in the BG dynamics.

### Conflict of interest statement

The authors declare that the research was conducted in the absence of any commercial or financial relationships that could be construed as a potential conflict of interest.

## Abbreviations

5HT: Serotonin
Ach: Acetylcholine
BG: Basal Ganglia
D1R: Dopamine D1 receptor
D1R-D2R: Dopamine D1 and D2 receptors
D2R: Dopamine D2 receptor
DA: Dopamine
DP: Direct Pathway
DRN: Dorsal Raphe Nucleus
GPe: Globus Pallidus externa
GPi: Globus Pallidus interna
IP: Indirect Pathway
MSN: Medium Spiny Neuron
NE: Norepinephrine
PD: Parkinson's Disease
PD-OFF: Parkinson's Disease- OFF medication
PD-ON: Parkinson's Disease- ON medication
R: Receptor
RL: Reinforcement Learning
SNc: Substantia Nigra pars compacta
STN: SubThalamic Nucleus
TD: Temporal Difference.
