# Supplementary material for: A network model of basal ganglia for understanding the roles of dopamine and serotonin in reward-punishment-risk based decision making
Source: Front Comput Neurosci. 2015 Jun 17;9:76. doi: 10.3389/fncom.2015.00076 (PMC4469836; doi:10.3389/fncom.2015.00076)
Supplement: Supplementary file 1 [file DataSheet1.DOCX]

**Supplementary material A**

The Genetic Algorithm (Goldberg, 1989) option set for optimization is given in the following table. Optimization toolbox 6.0, Matlab R2011a, The Mathworks Inc. is used.

*Option set for the GA tool*

| **Option** | **Value** |
| --- | --- |
| Population Size | 20 |
| Crossover fraction | 0.8 |
| Elite count | 4 |
| Generation time | 1000 |
| Function tolerance | 1 e-6 |
| Cost function | (Expt measure - Sims measure)^2^ |
