# Supplementary material for: A network model of basal ganglia for understanding the roles of dopamine and serotonin in reward-punishment-risk based decision making
Source: Front Comput Neurosci. 2015 Jun 17;9:76. doi: 10.3389/fncom.2015.00076 (PMC4469836; doi:10.3389/fncom.2015.00076)
Supplement: Supplementary file 2 [file DataSheet2.DOCX]

**Supplementary material B**

This material deals with the analysis of different subsets of the group containing α_D1,_ α_D2,_ α_D1D2_. Since the final decision only depends on the relative magnitudes of the three terms defined above in eqns. (2.3.8, 2.3.9), the α parameters are varied at the most two at a time. Thus the different cases that can be analysed from this material are summarized in the following table. Here, ‘*’ indicates that corresponding coefficient is varied, while ‘1’ indicates that it is fixed at 1.

|  | α_D1_ | α_D2_ | α_D1D2_ |
| --- | --- | --- | --- |
| Case 1 | * | 1 | 1 |
| Case 2 | 1 | * | 1 |
| Case 3 | 1 | 1 | * |
| Case 4 | * | * | 1 |
| Case 5 | * | 1 | * |
| Case 6 | * | * | * |

The results (shown in supplementary material B) depict the ability of each of the cases to capture the functions of serotonin in risk and reward-punishment sensitivity.

The experiments analysed are that reported in the manuscript:

1. Long et al. (2009),
2. Cools et al. (2008), and
3. Bodi et al. (2009),

The color bar defines the normalised error in meeting the mean experimental value by the mean simulation value in each of the experiment (I, II and III) as explained.

# Long et al. (2009)

Representing normalised Error = ((expt-sims)/expt)^2 summated for the mean probability of choosing the safe choices in the Overall [all] , UEV and the EEV cases

Error = ((expt_all_-sims_all_)/expt_all_)^2 + ((expt_UEV_-sims_UEV_)/expt_UEV_)^2 + ((expt_EEV_-sims_EEV_)/expt_EEV_)^2

The Expt values are given in the following table:

|  | RTD | BAL |
| --- | --- | --- |
| All | 0.432 | 0.533538 |
| UEV | 0.611111 | 0.733333 |
| EEV | 0.287037 | 0.353704 |

## Rapid tryptophan depletion condition

The first row represents cases 1-3 in which the appropriate parameter (noted in the legend for that data plot) is varied, and the others in set (α_D1_, α_D2_, α_D1D2_) are fixed to 1. The subsequent rows show cases 4 and 5 where α_D1D2_ and α_D2_ are fixed to 1 respectively, and the other two parameters vary across axes. The later rows present the more general case 6 as a function of (α_D2_, α_D1D2_), for a given α_D1._

| 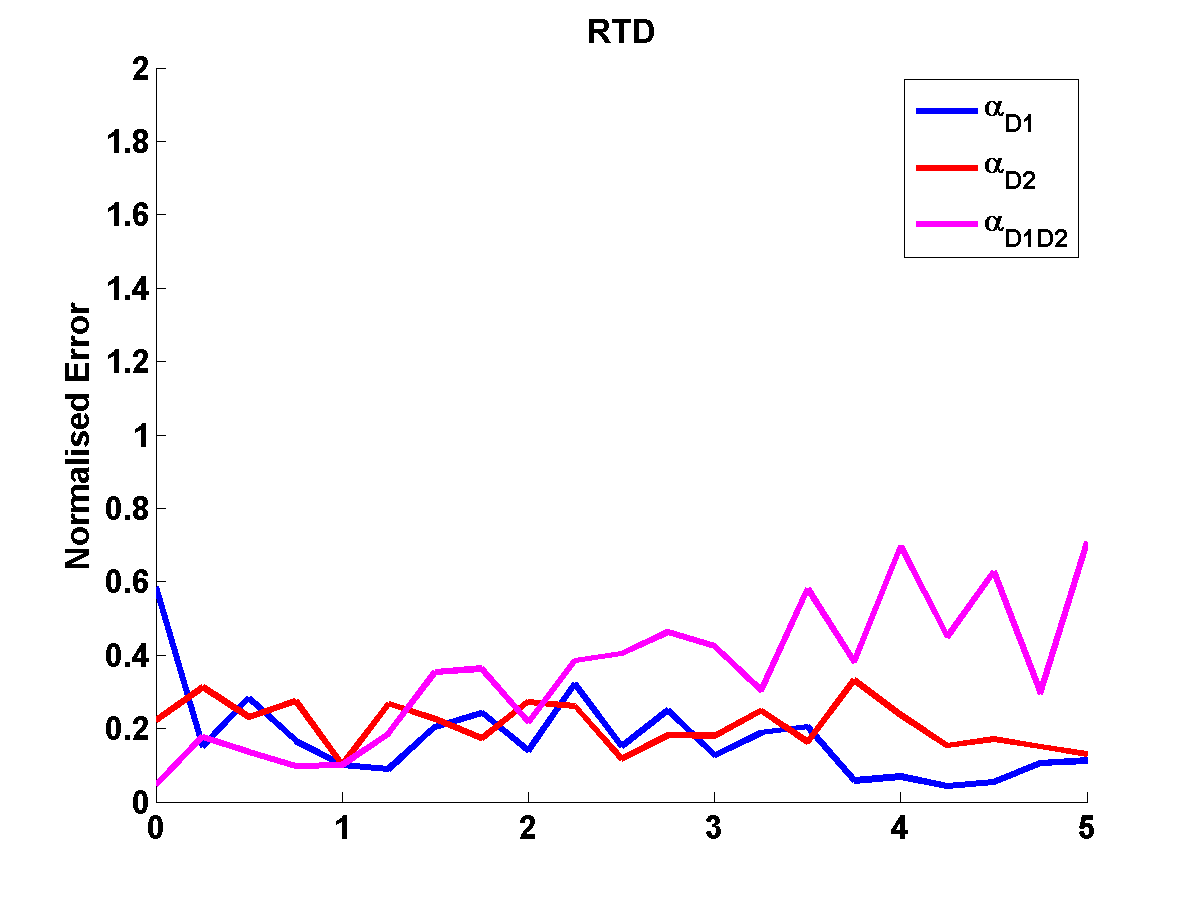  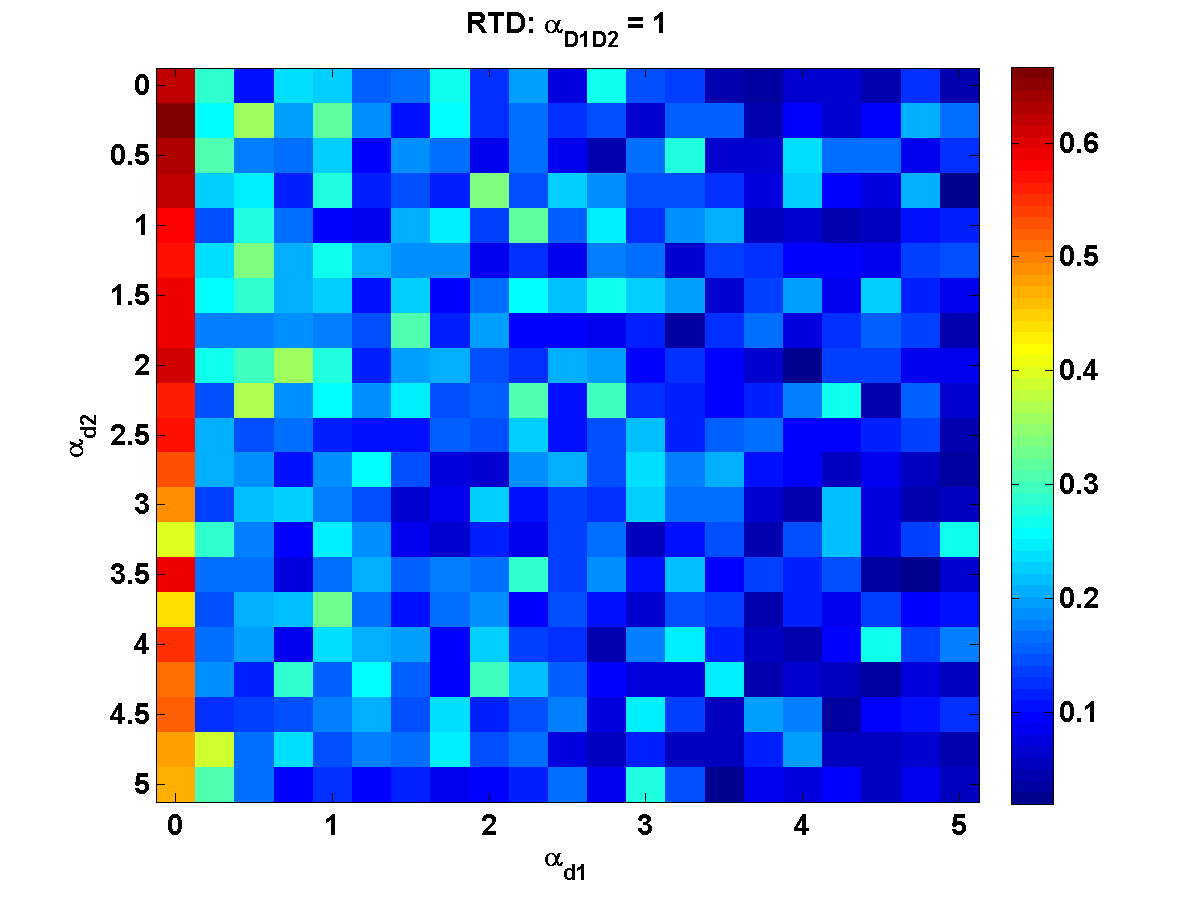  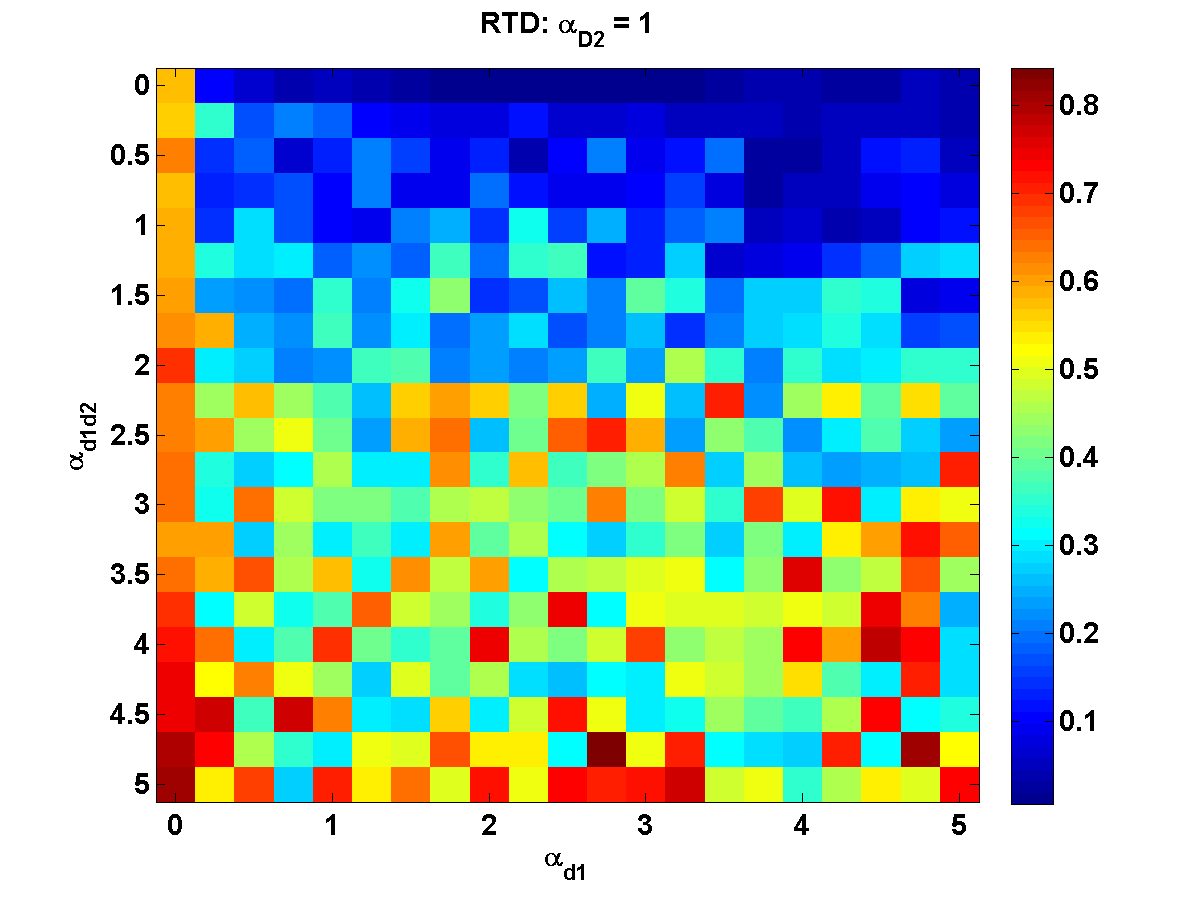  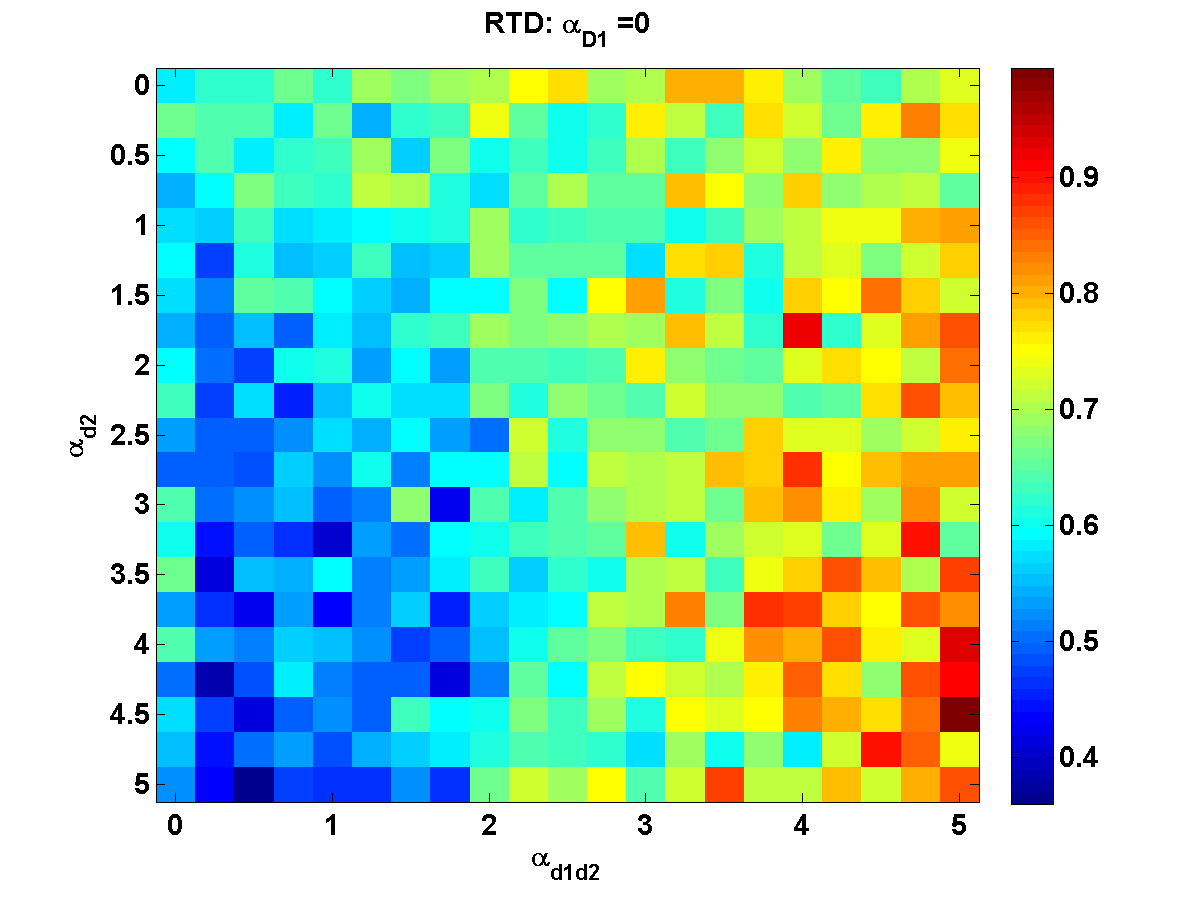  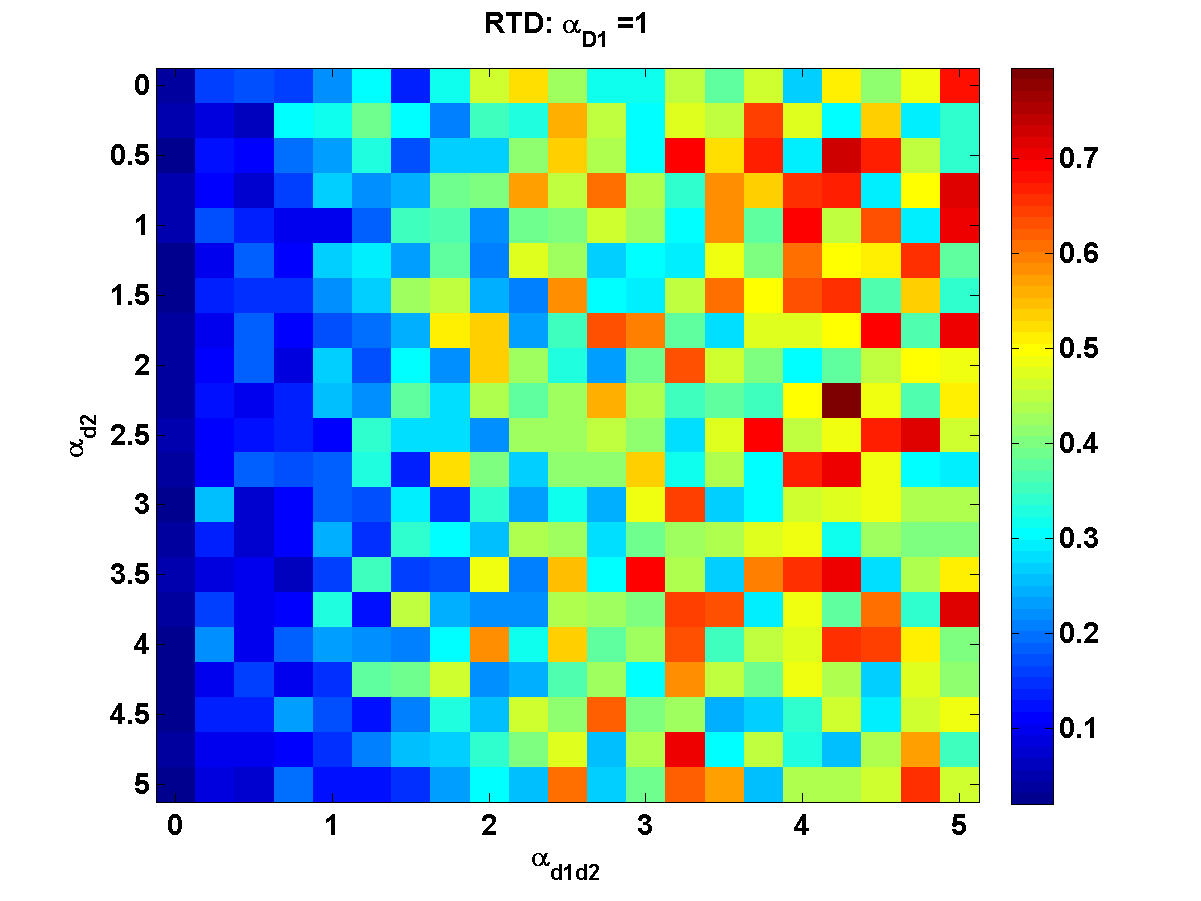  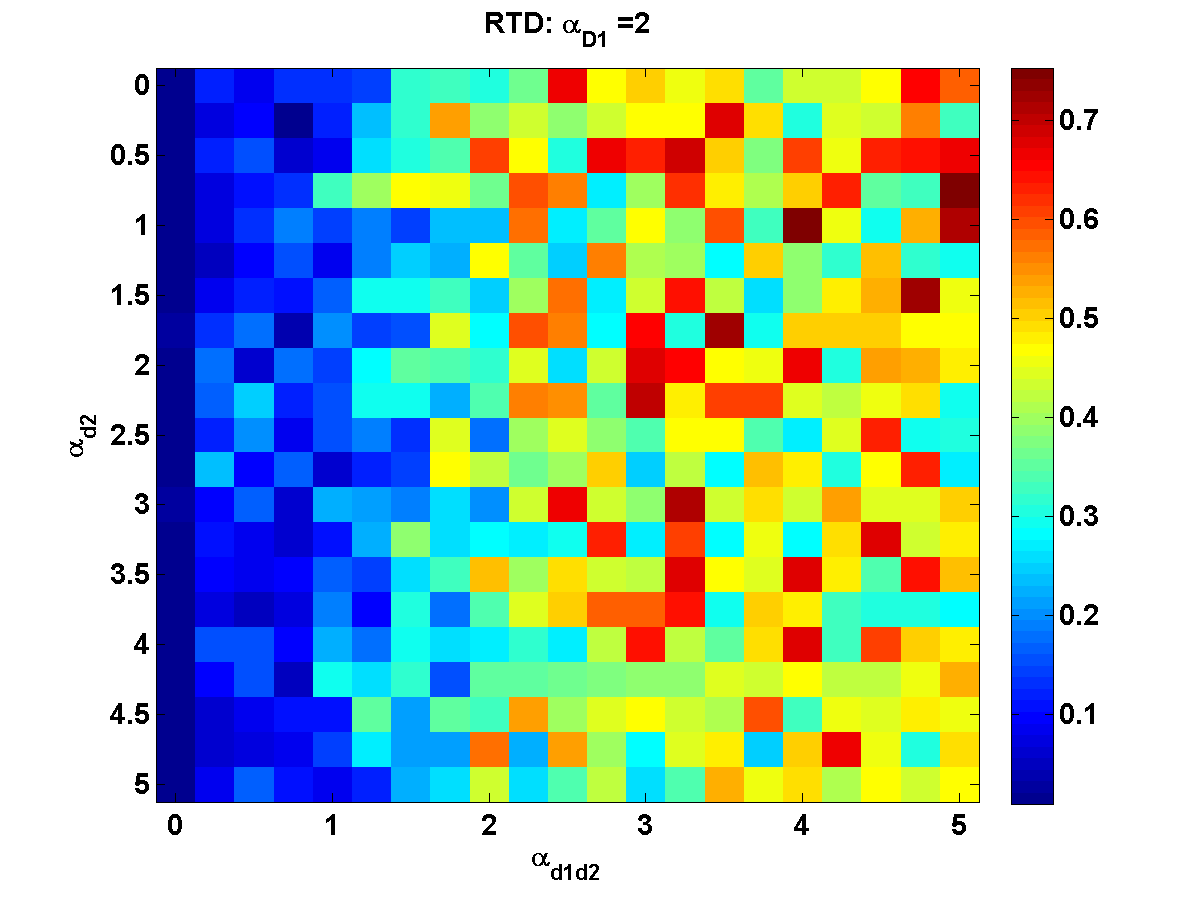  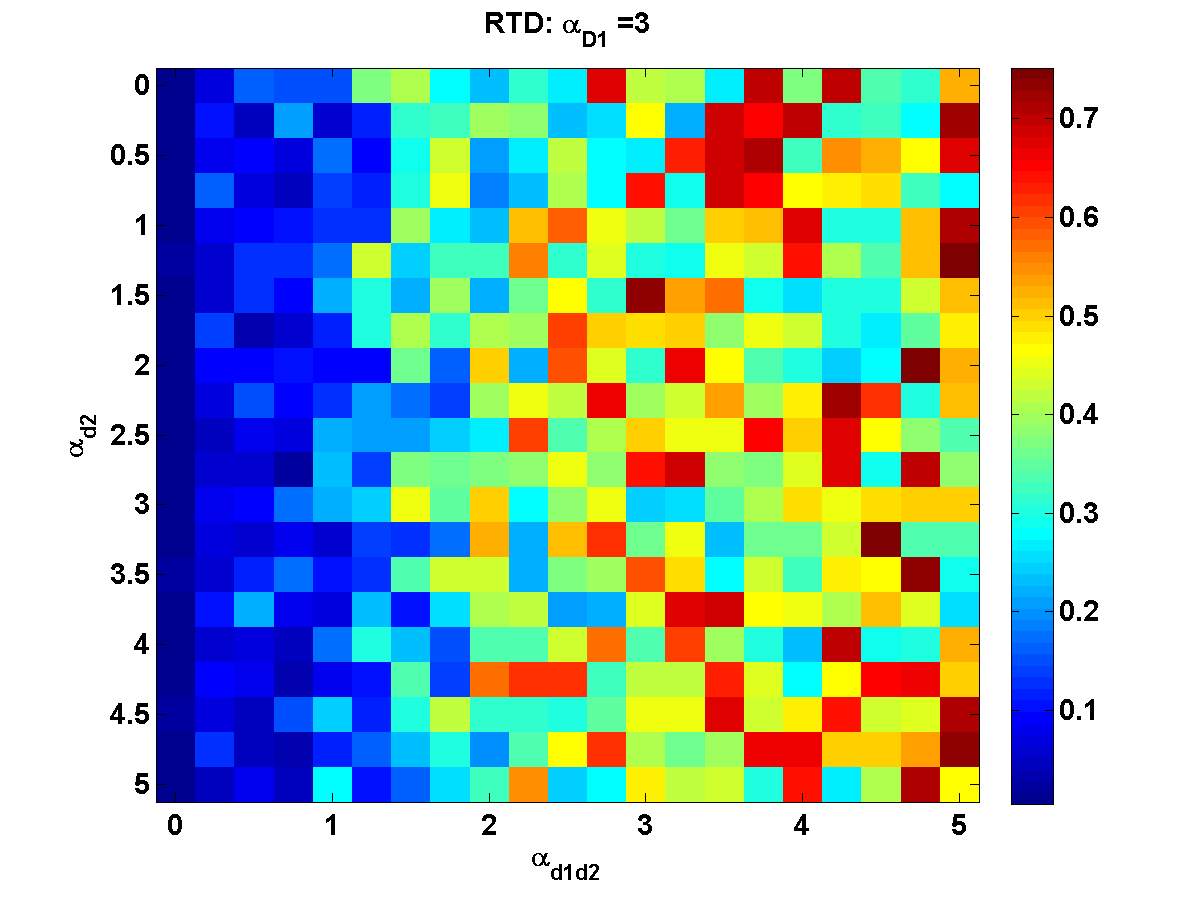  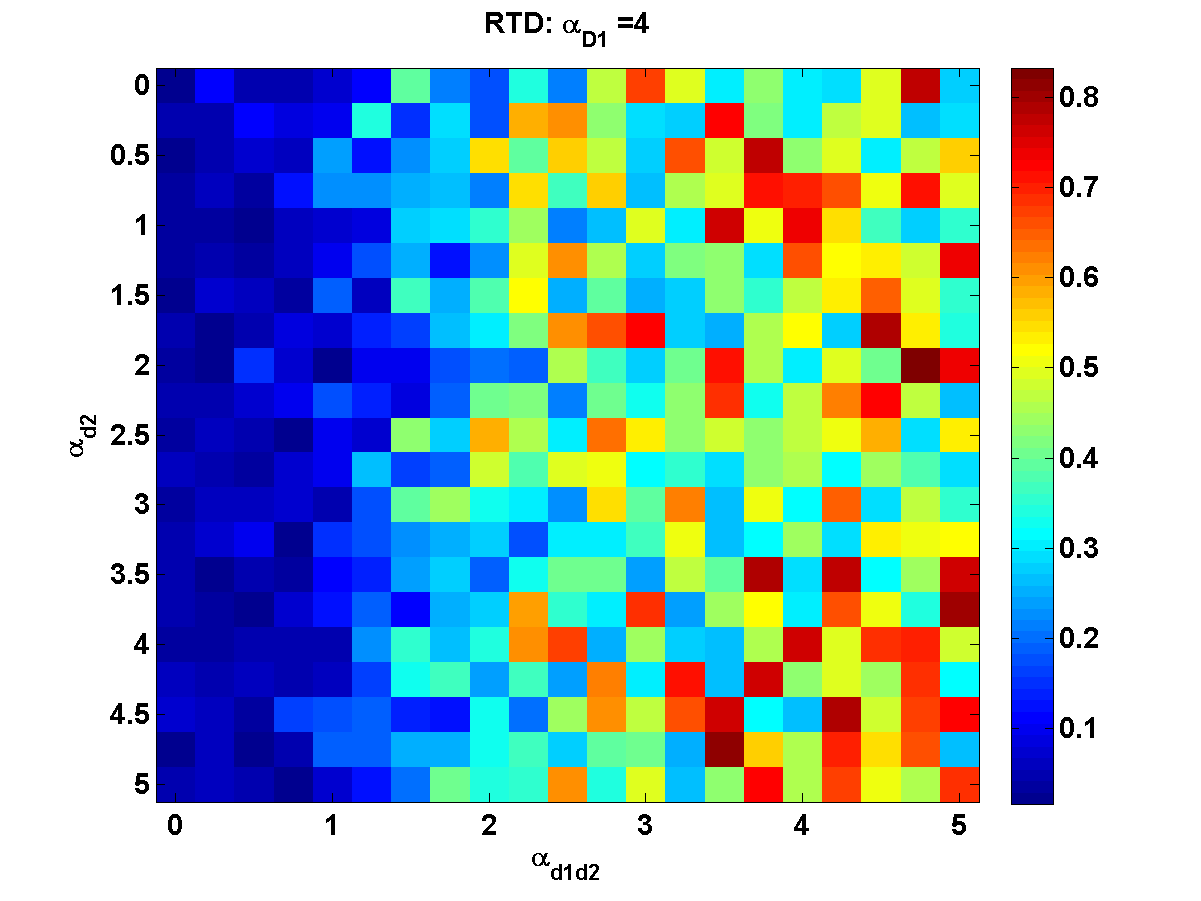  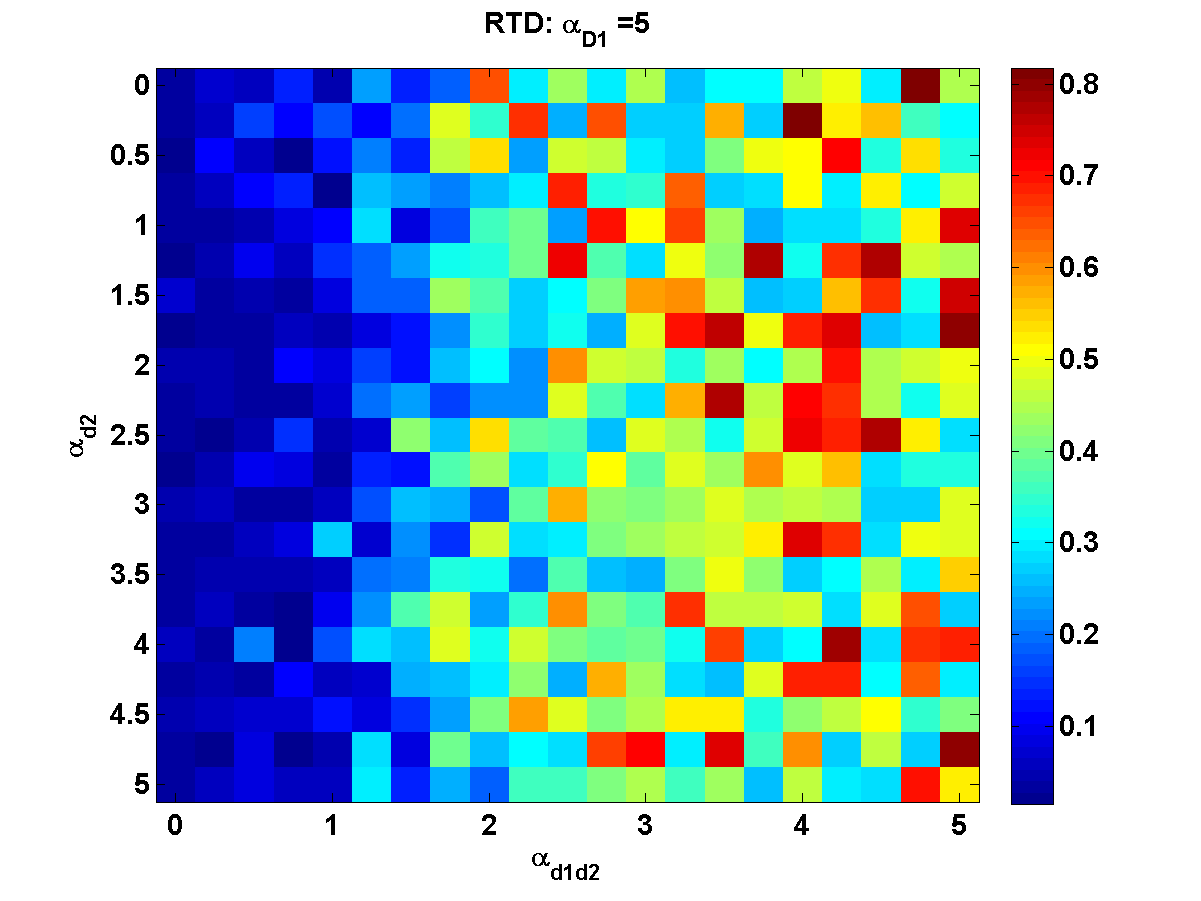 |
| --- |

## Tryptophan balance condition:

The first row represents cases 1-3 in which the appropriate parameter (noted in the legend for that data plot) is varied, and the others in set (α_D1_, α_D2_, α_D1D2_) are fixed to 1. The subsequent rows show cases 4 and 5 where α_D1D2_ and α_D2_ are fixed to 1 respectively, and the other two parameters vary across axes. The later rows present the more general case 6 as a function of (α_D2_, α_D1D2_), for a given α_D1._

| 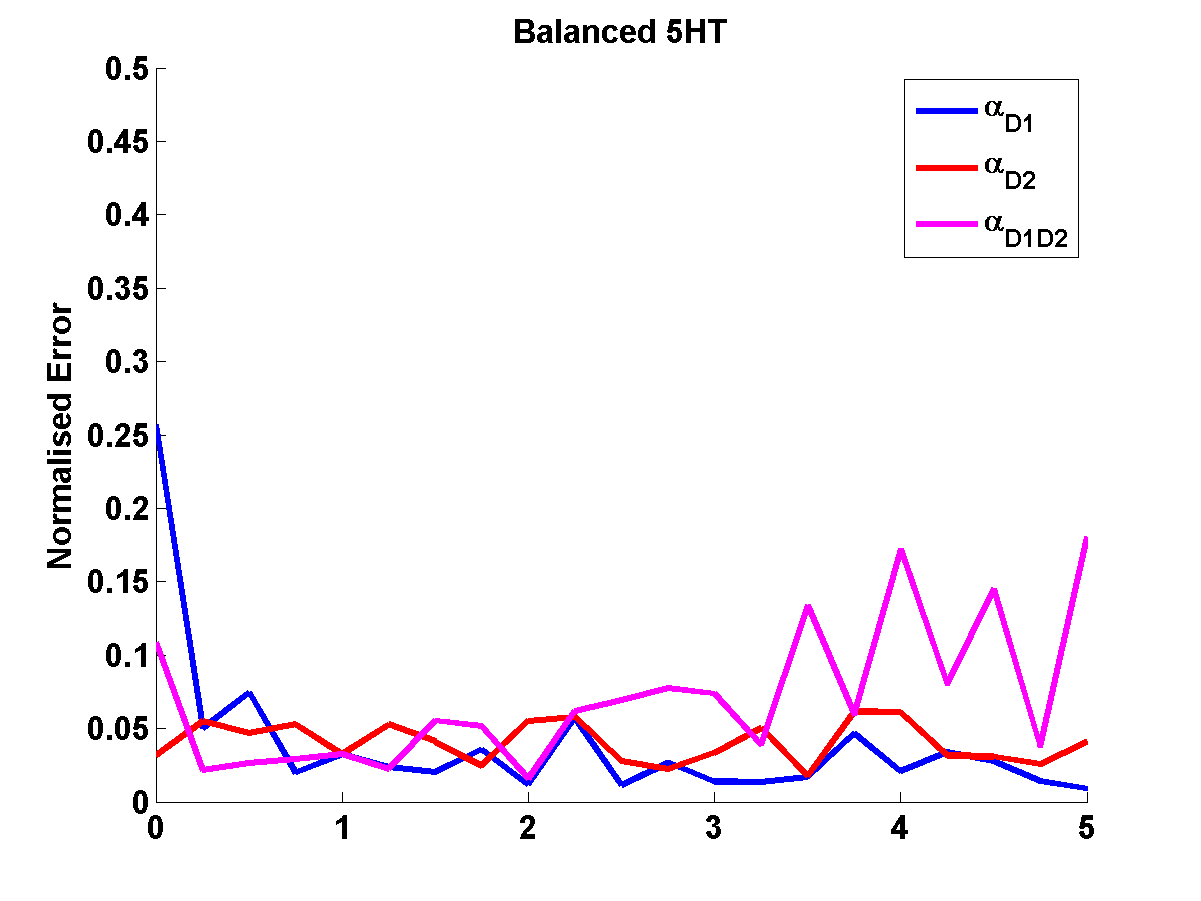  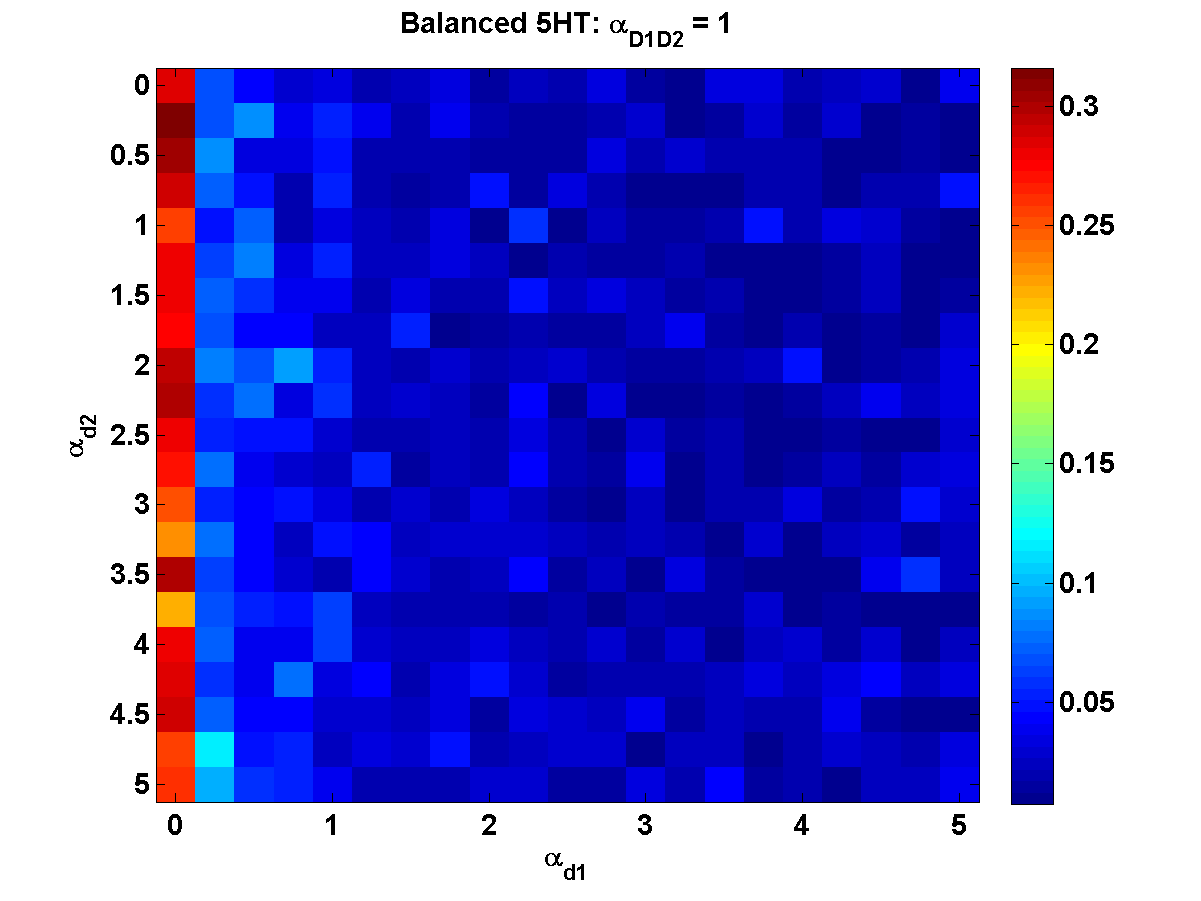  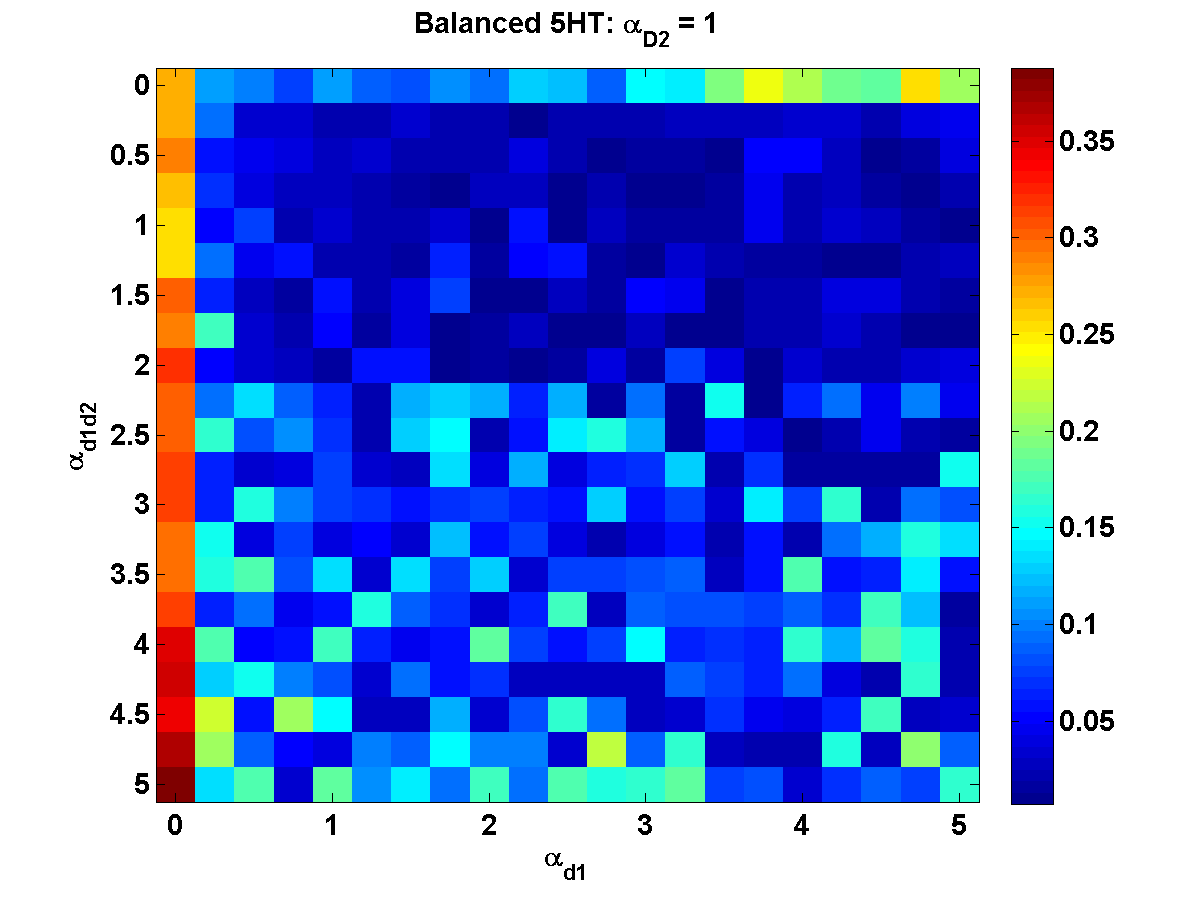  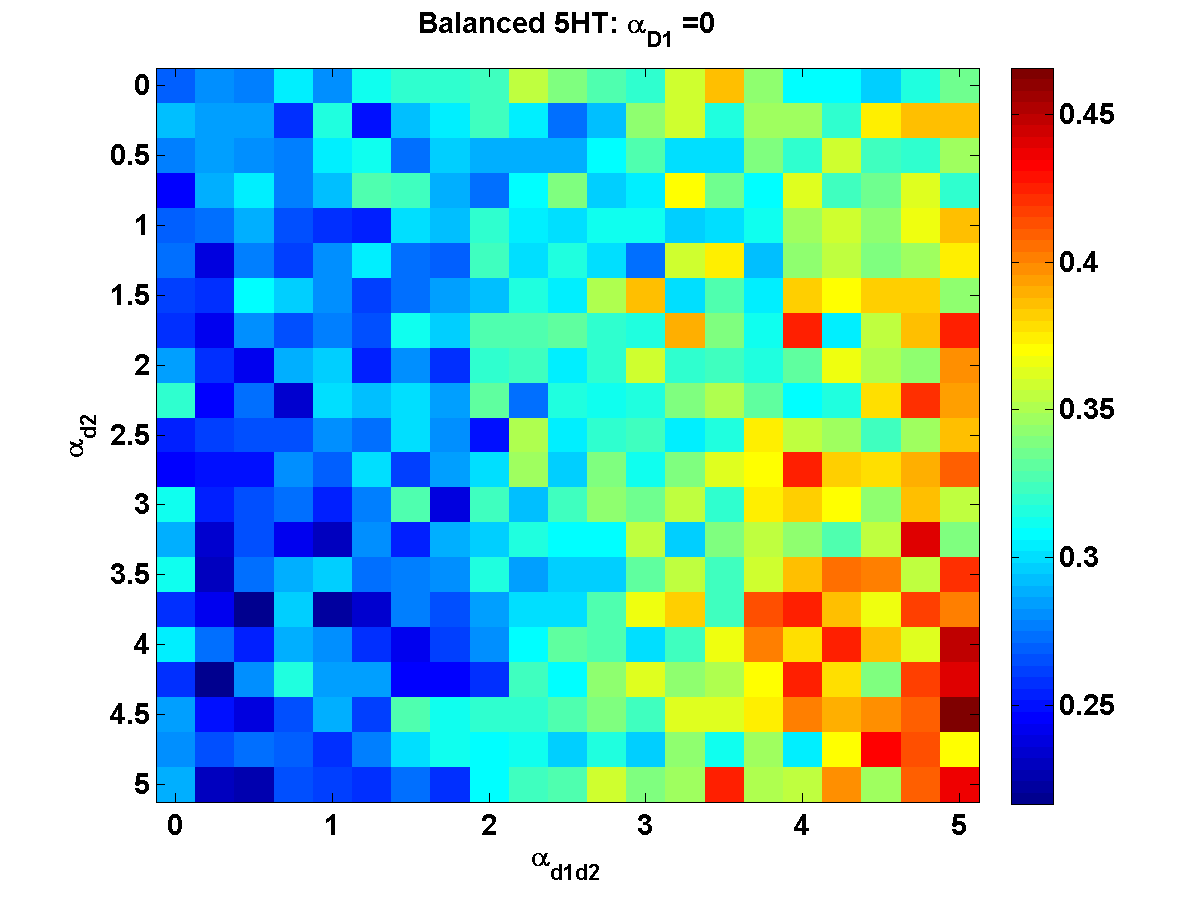  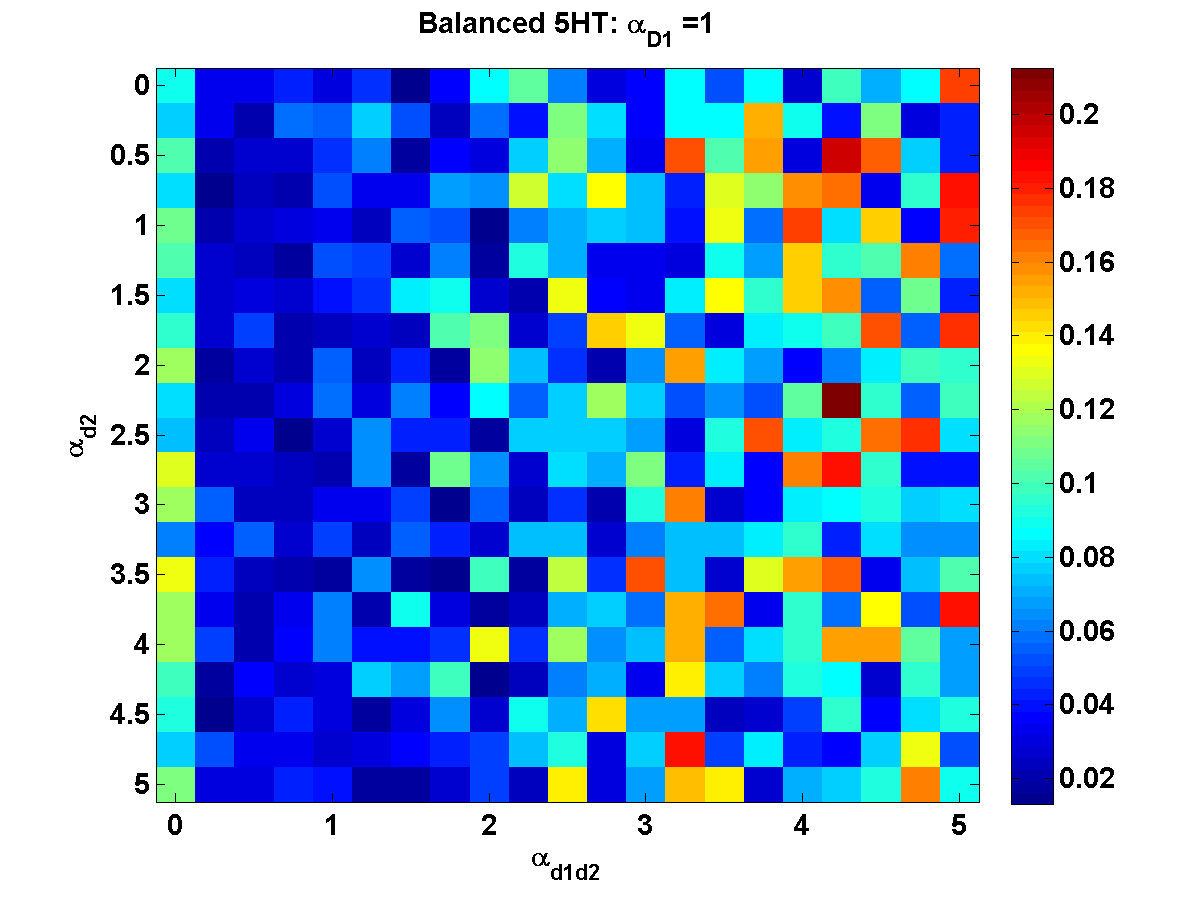  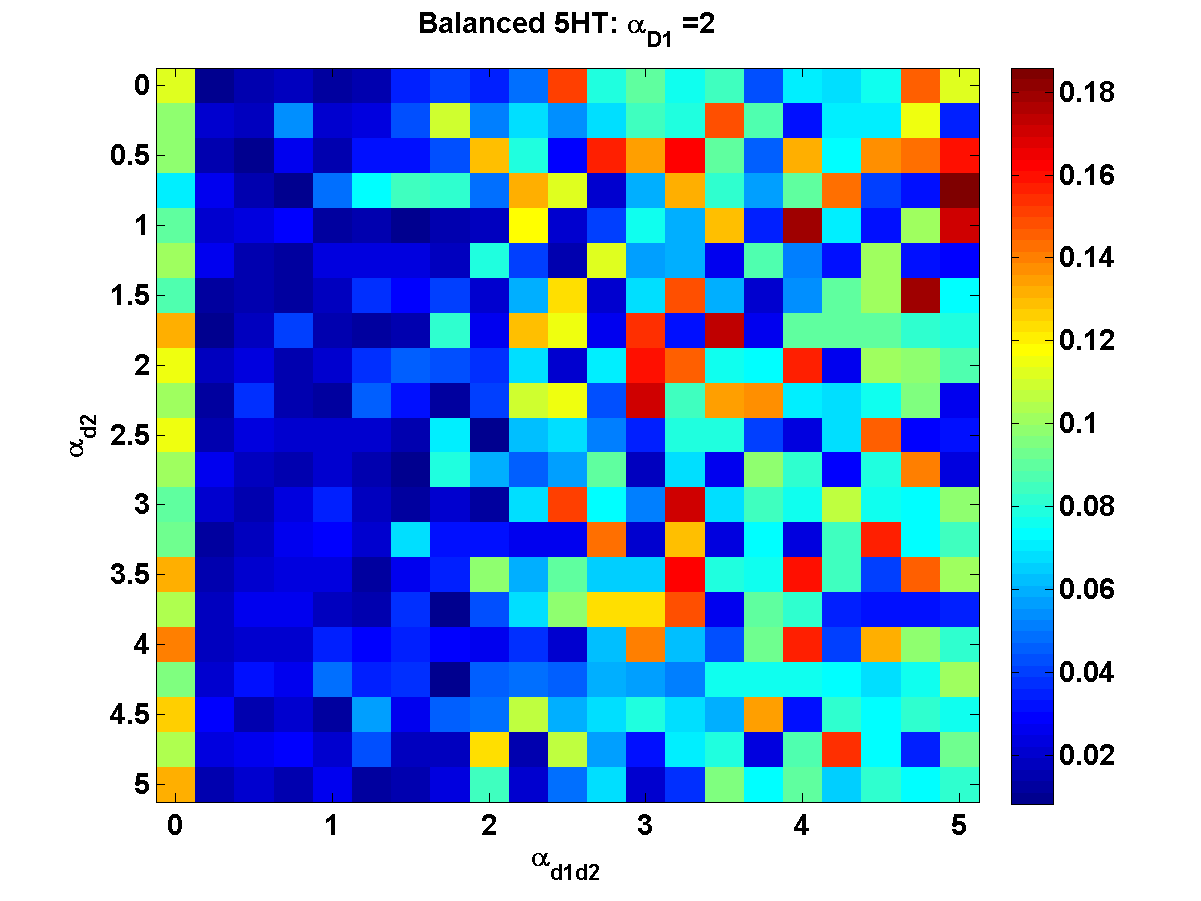  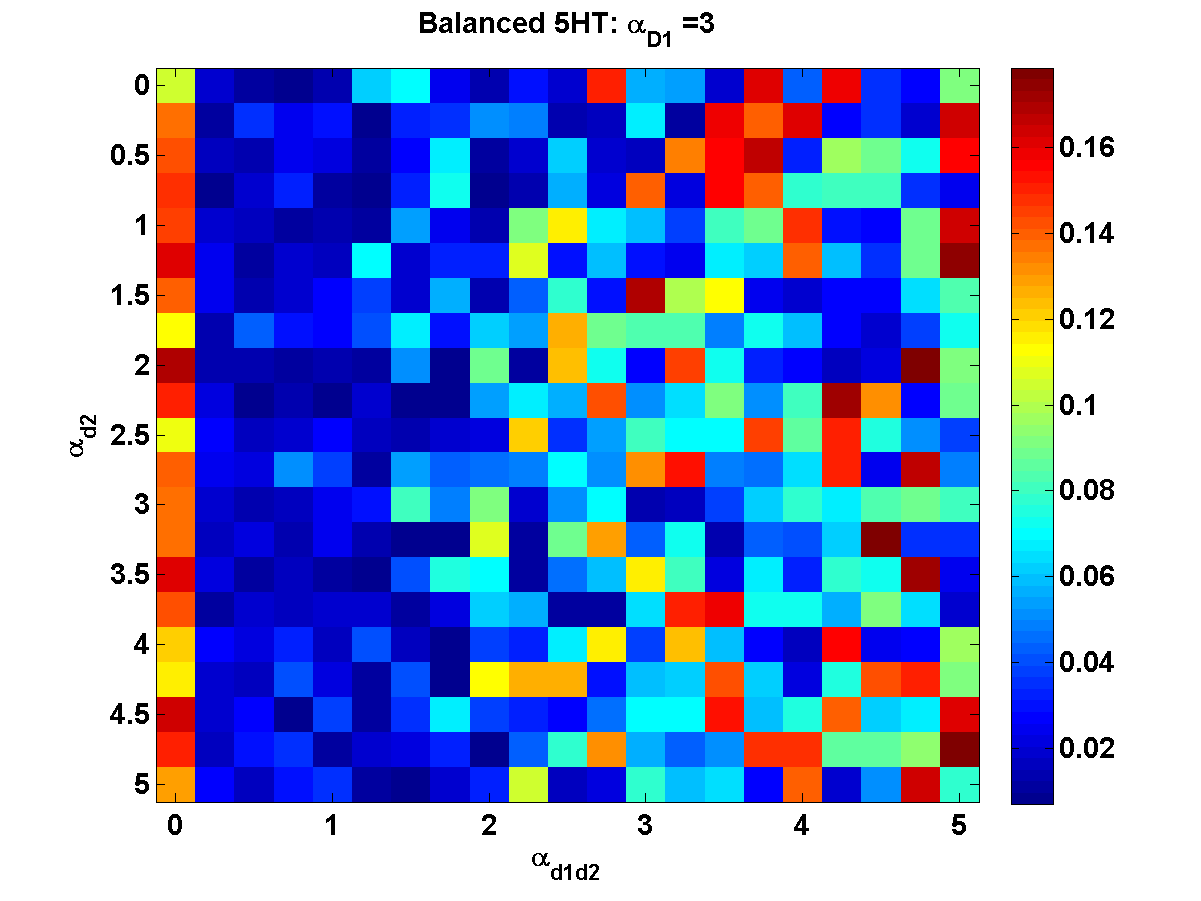  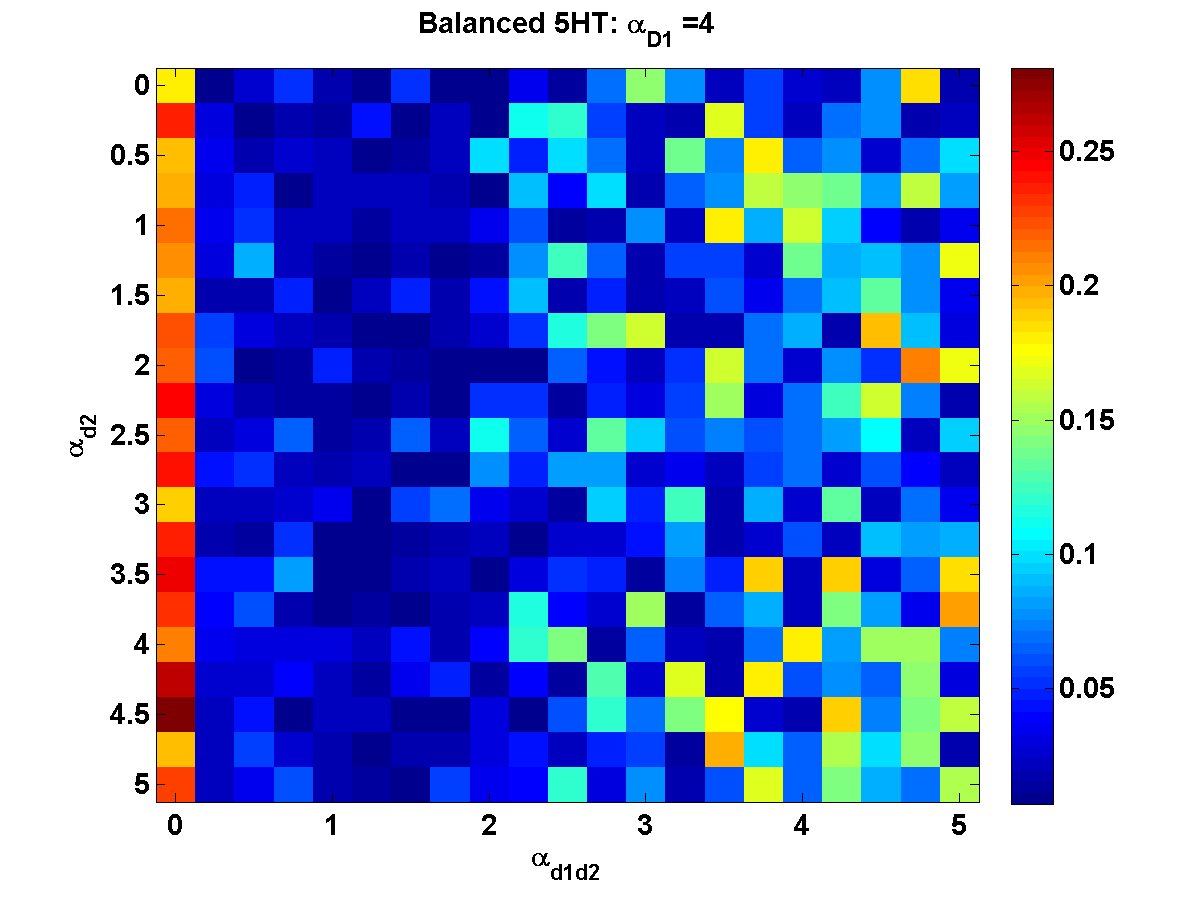  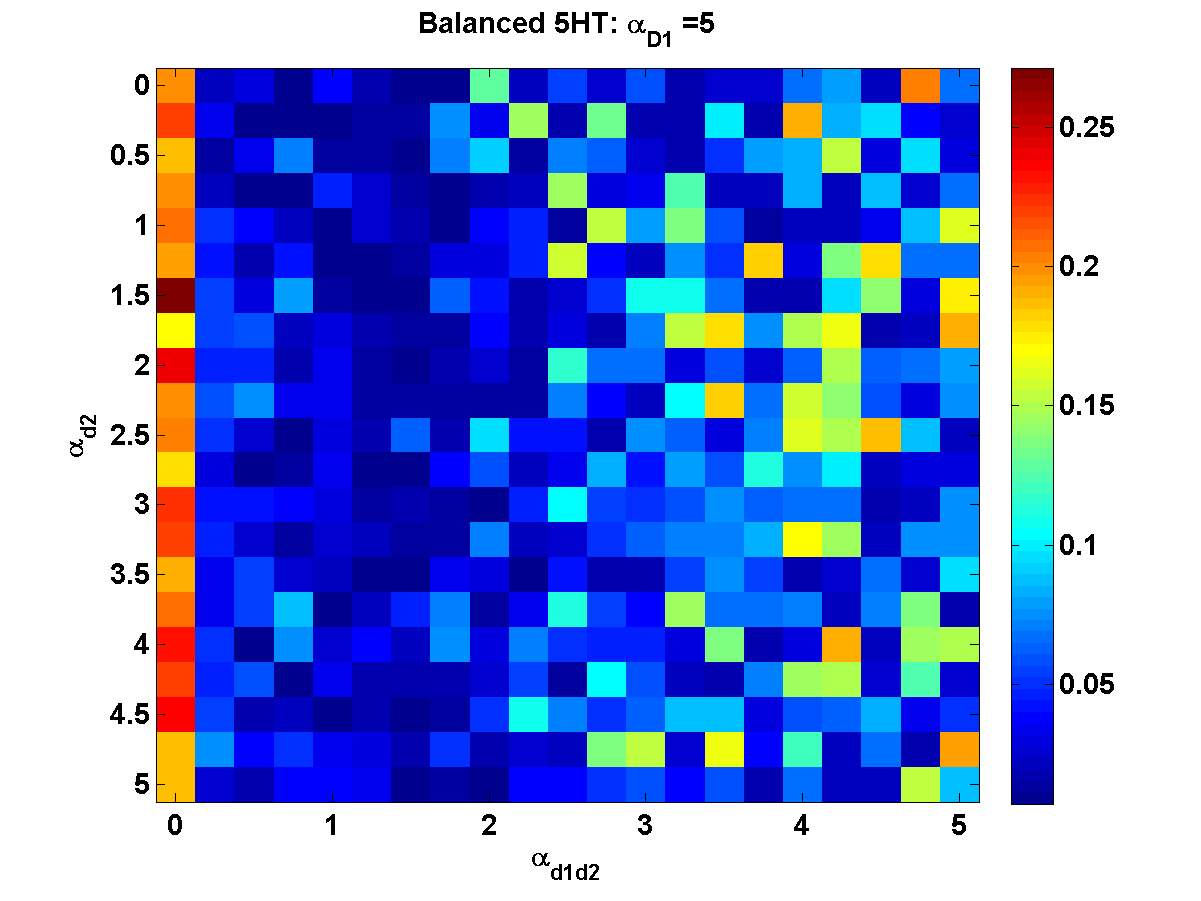 |
| --- |

# Cools et al. (2008)

Representing normalised Error = ((expt-sims)/expt)^2 summated for the mean error (= sqrt(error counts)) as the function of valences (reward prediction [rp], punishment prediction [pp]) and conditions (unexpected reward [ur], unexpected punishment [up]).

Error = ((expt_rp_-sims_rp_)/expt_rp_)^2 + ((expt_pp_-sims_pp_)/expt_pp_)^2 + ((expt_ur_-sims_ur_)

/ expt_ur_)^2 + ((expt_up_-sims_up_)/expt_up_)^2

The desired values are given in the following table:

|  | RTD | BAL |
| --- | --- | --- |
| rp | 11.71782 | 10.00218 |
| pp | 10.04999 | 16.24009 |
| ur | 10.91652 | 13.77223 |
| up | 11.02578 | 13.98352 |

## Rapid tryptophan depletion condition:

The first row represents cases 1-3 in which the appropriate parameter (noted in the legend for that data plot) is varied, and the others in set (α_D1_, α_D2_, α_D1D2_) are fixed to 1. The subsequent rows show cases 4 and 5 where α_D1D2_ and α_D2_ are fixed to 1 respectively, and the other two parameters vary across axes. The later rows present the more general case 6 as a function of (α_D2_, α_D1D2_), for a given α_D1._

| 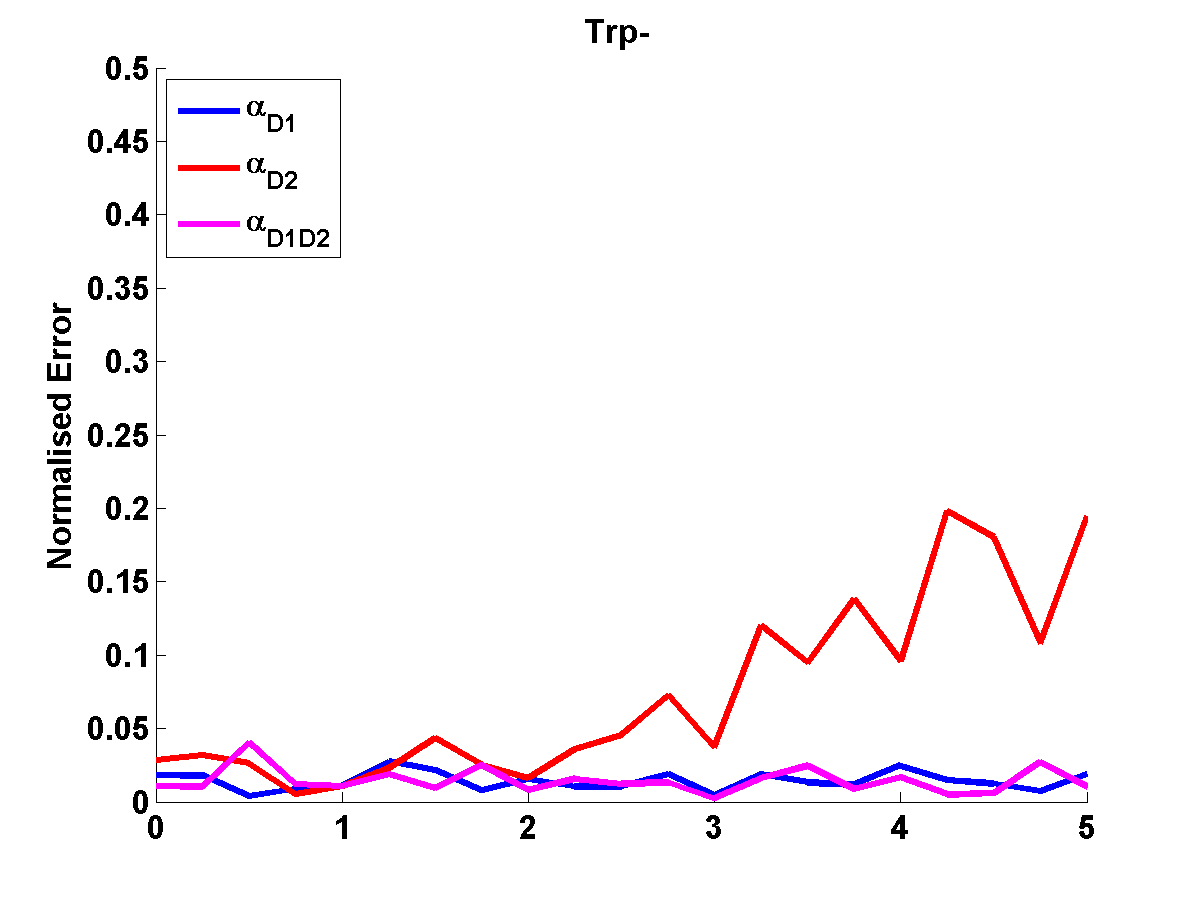  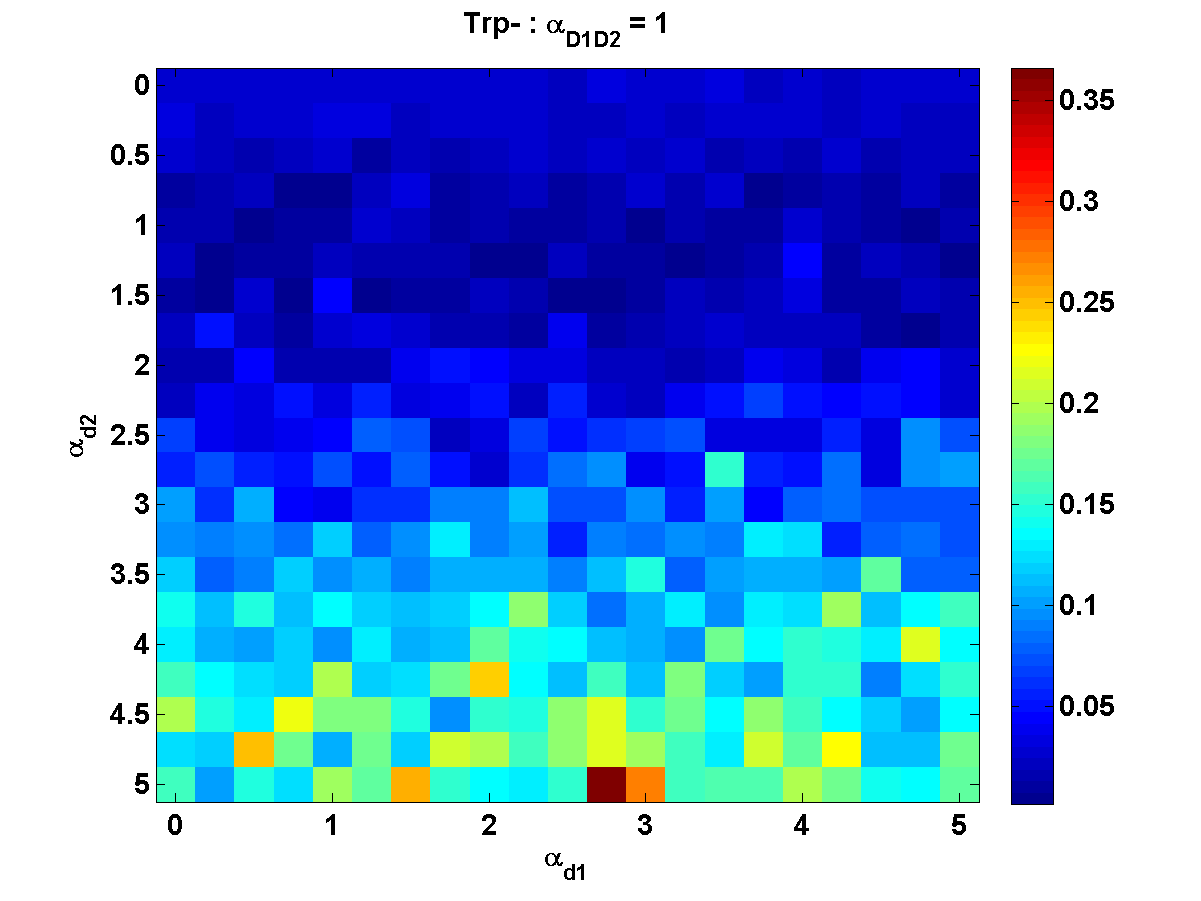  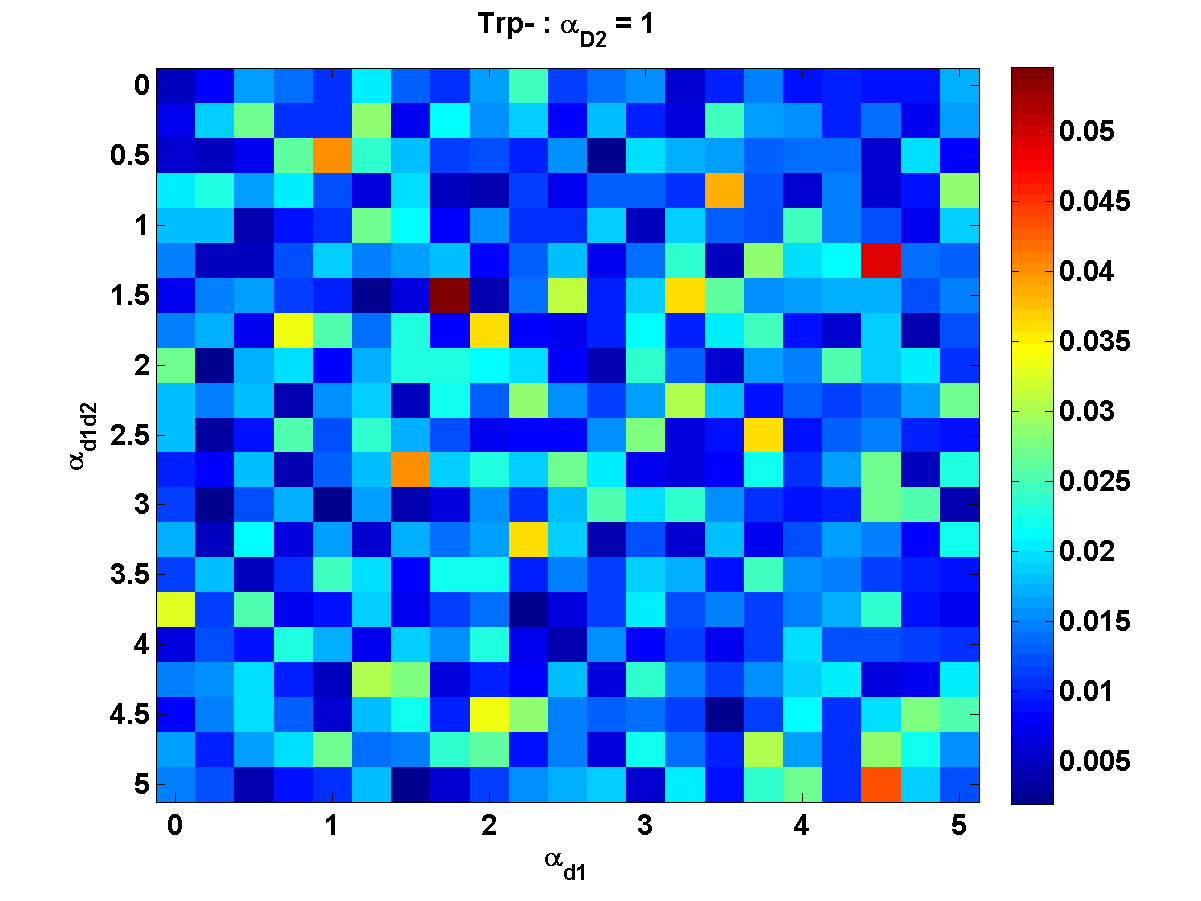  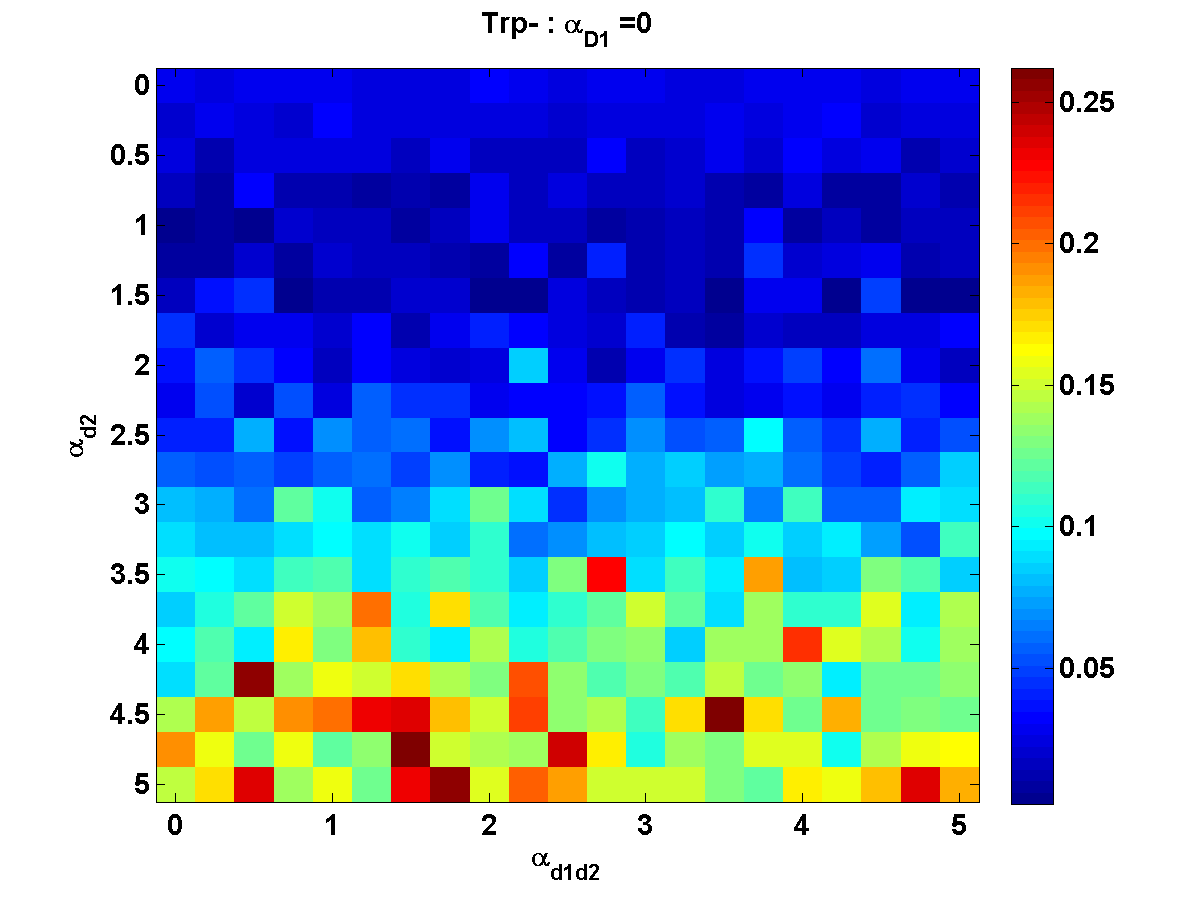  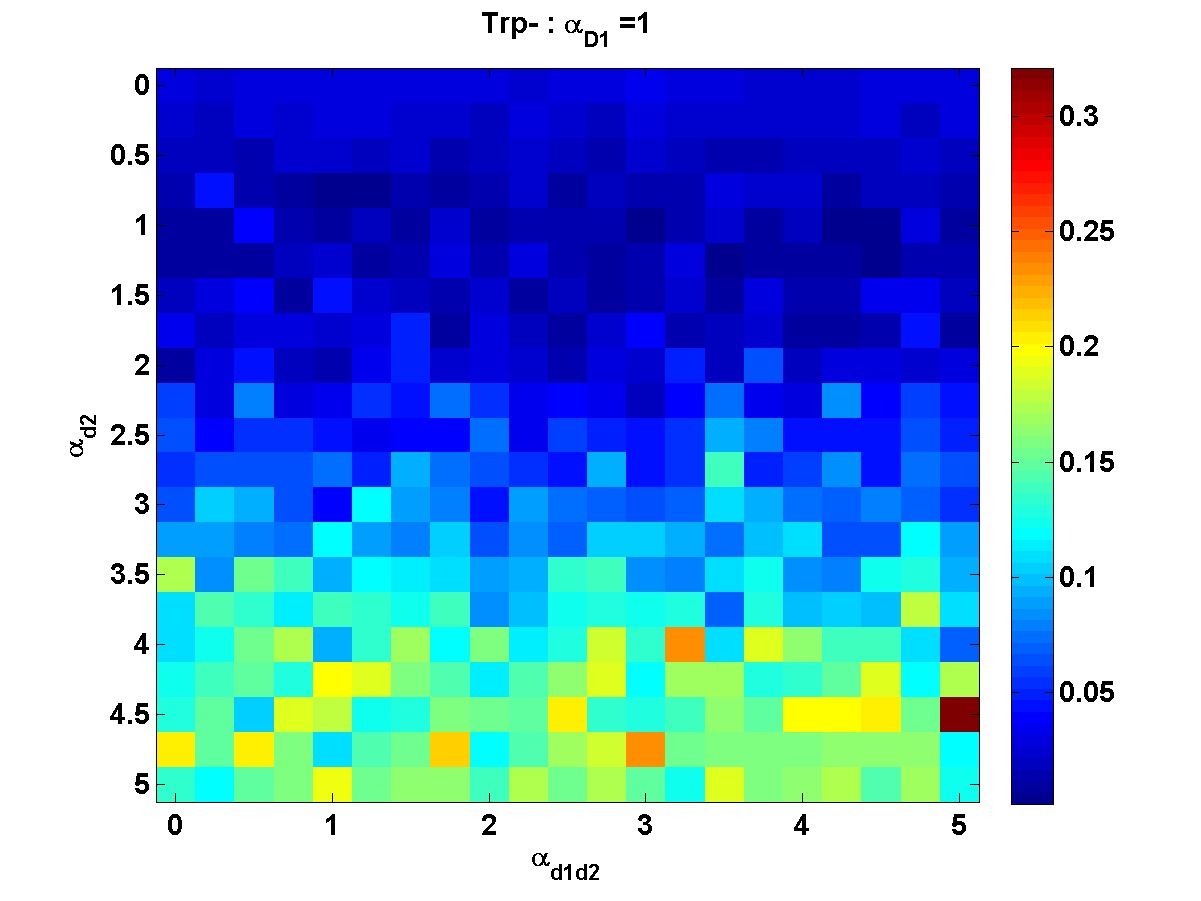  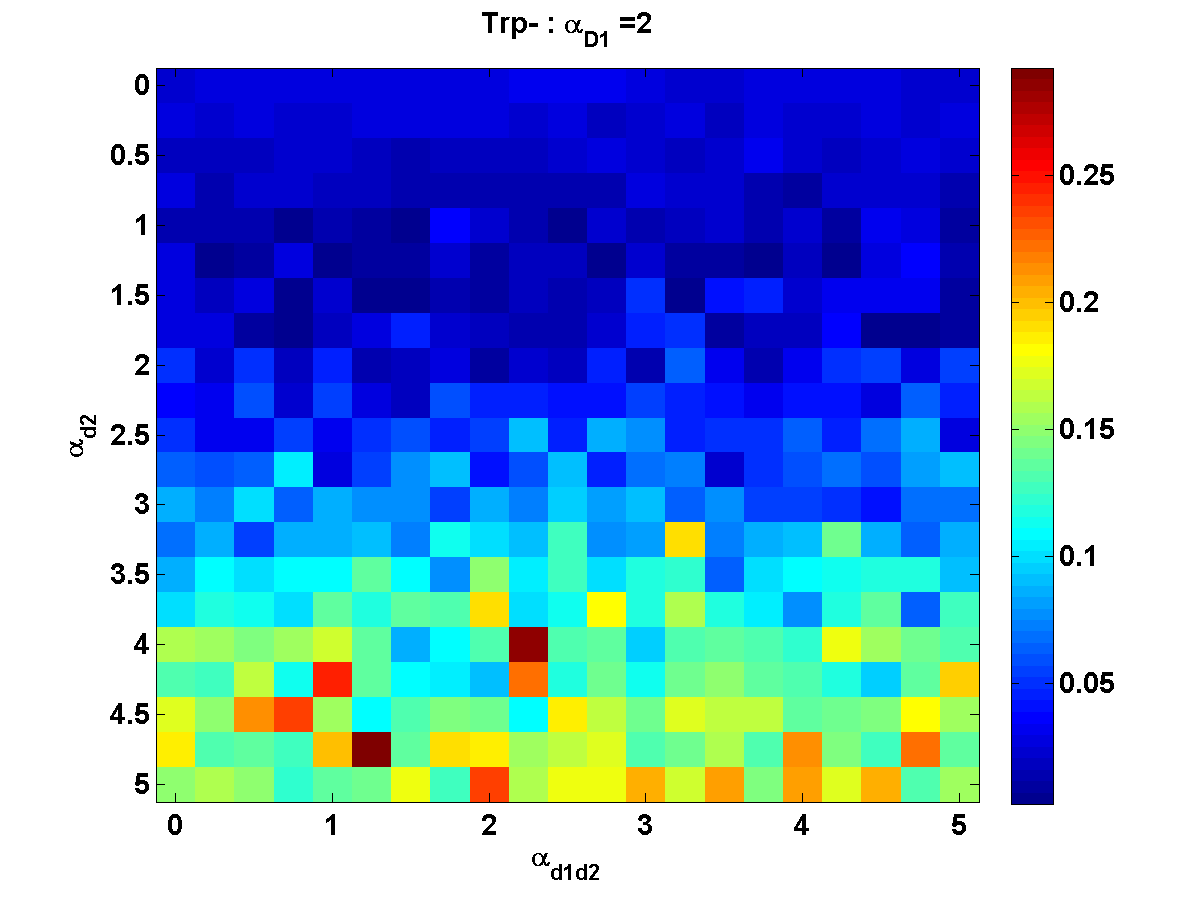  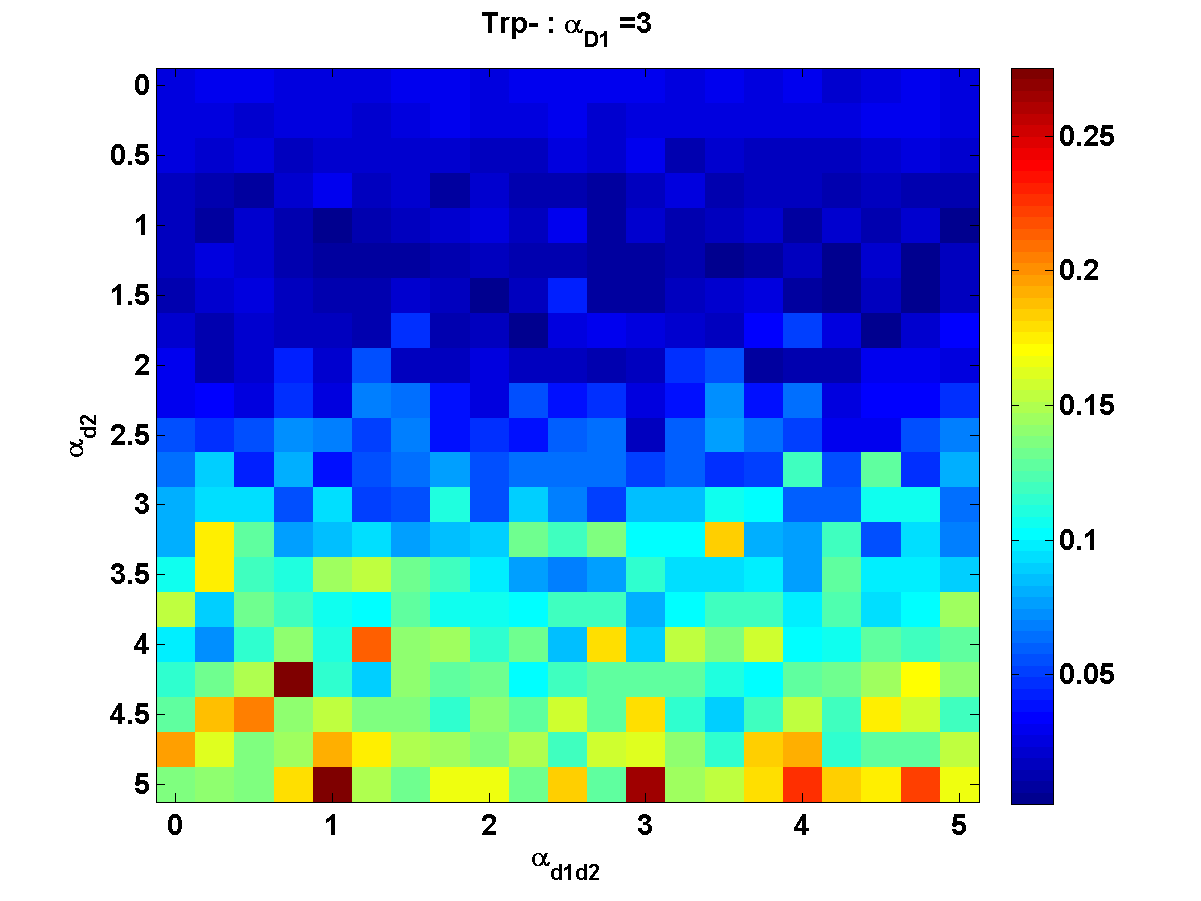  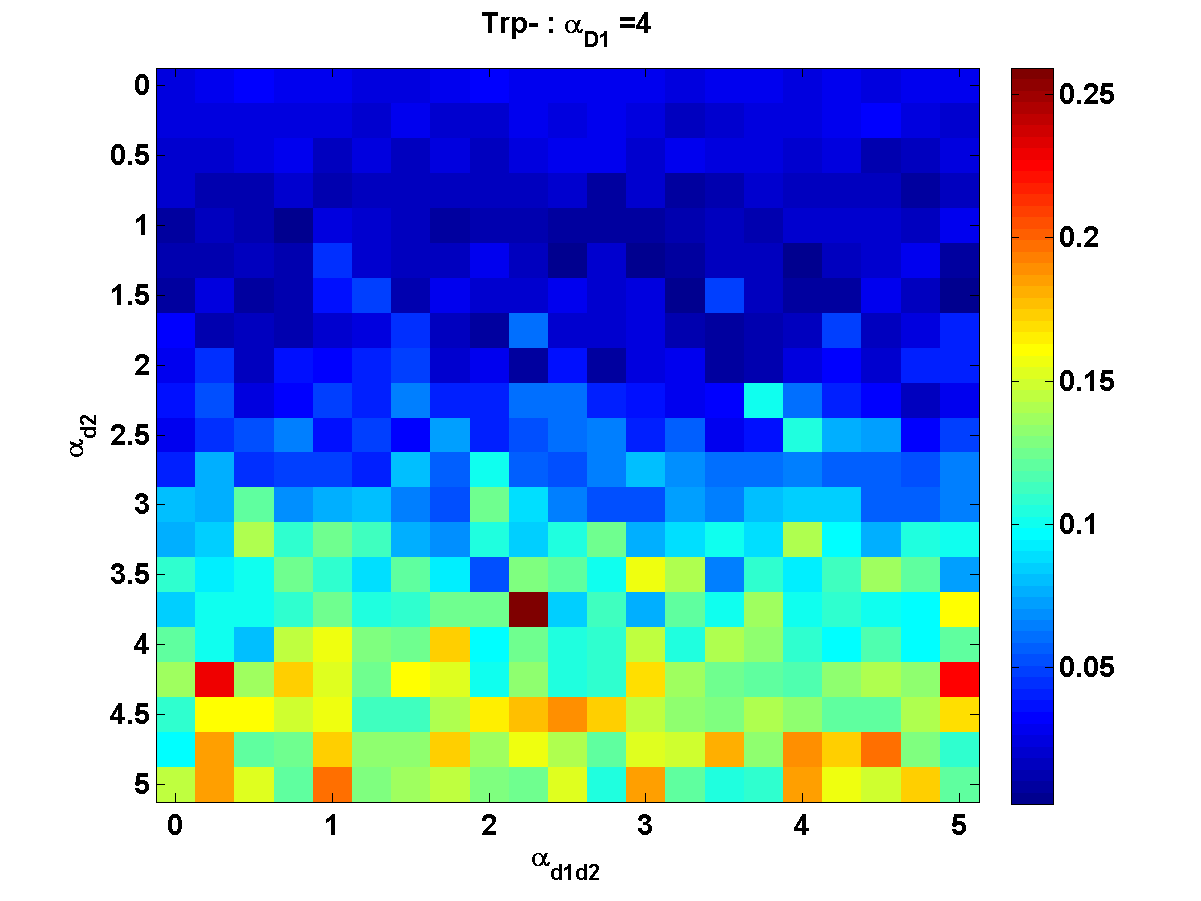  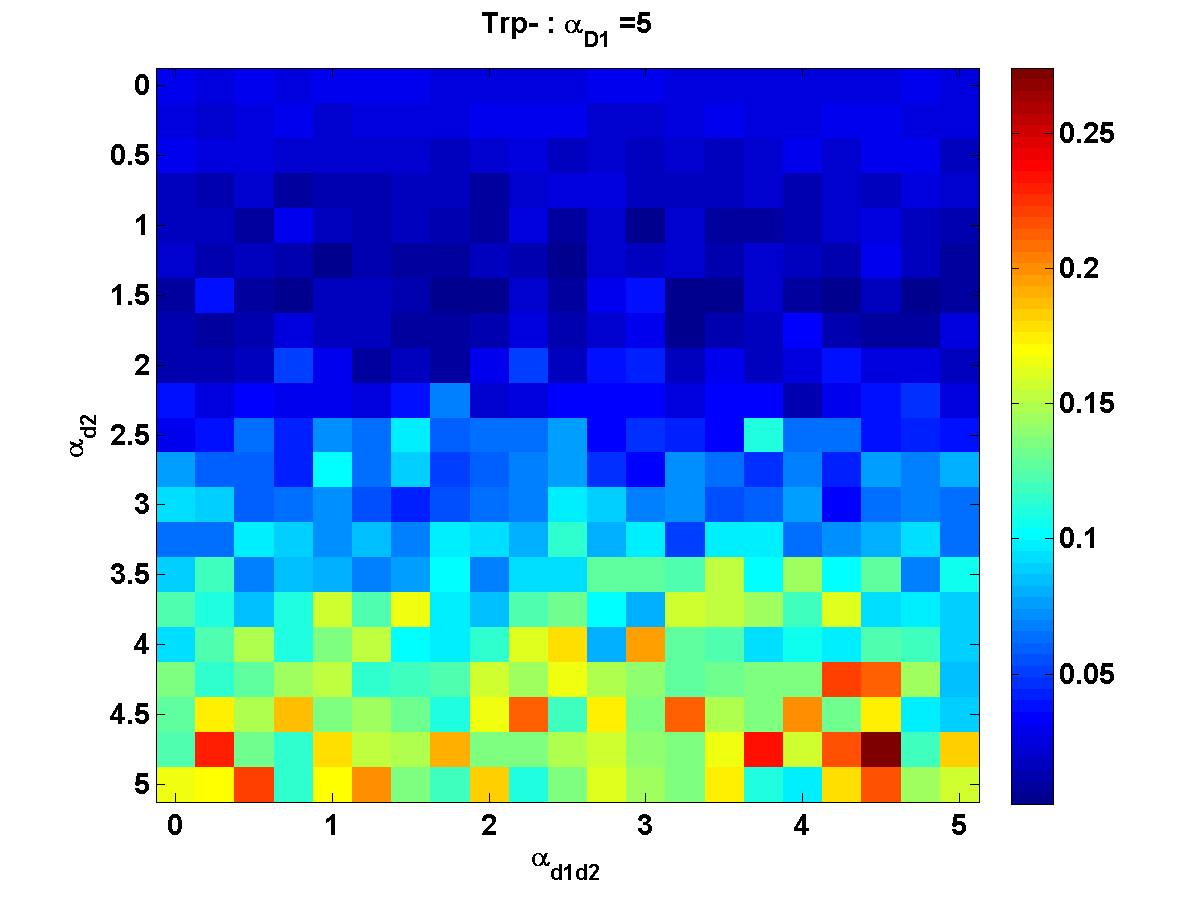 |
| --- |

## Tryptophan balance condition:

The first row represents cases 1-3 in which the appropriate parameter (noted in the legend for that data plot) is varied, with the others in set (α_D1_, α_D2_, α_D1D2_) are fixed to 1. The subsequent rows present cases 4 and 5 where α_D1D2_ and α_D2_ are fixed to 1, respectively, and the other two parameters varying across axes. The later rows present the more general case 6 as a function of (α_D2_, α_D1D2_), for a given α_D1_.

| 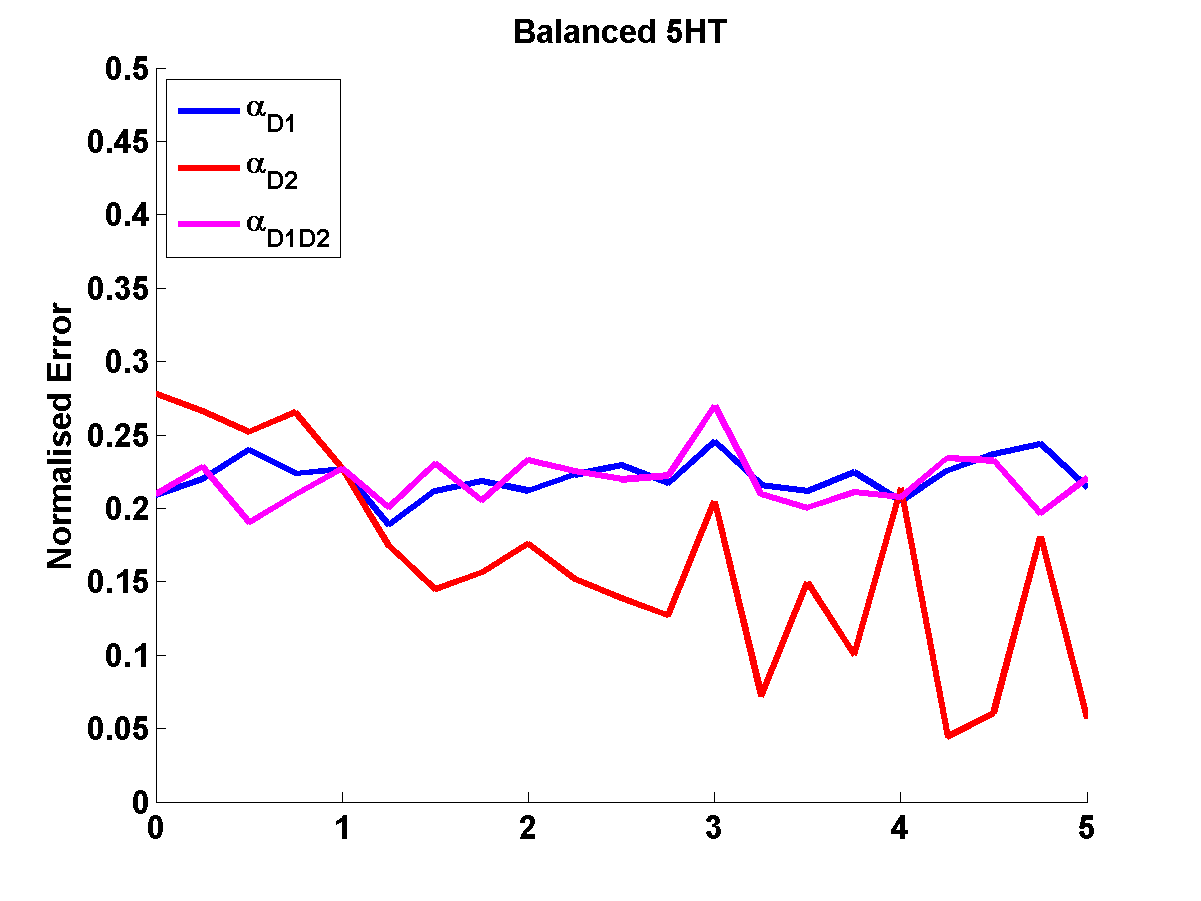  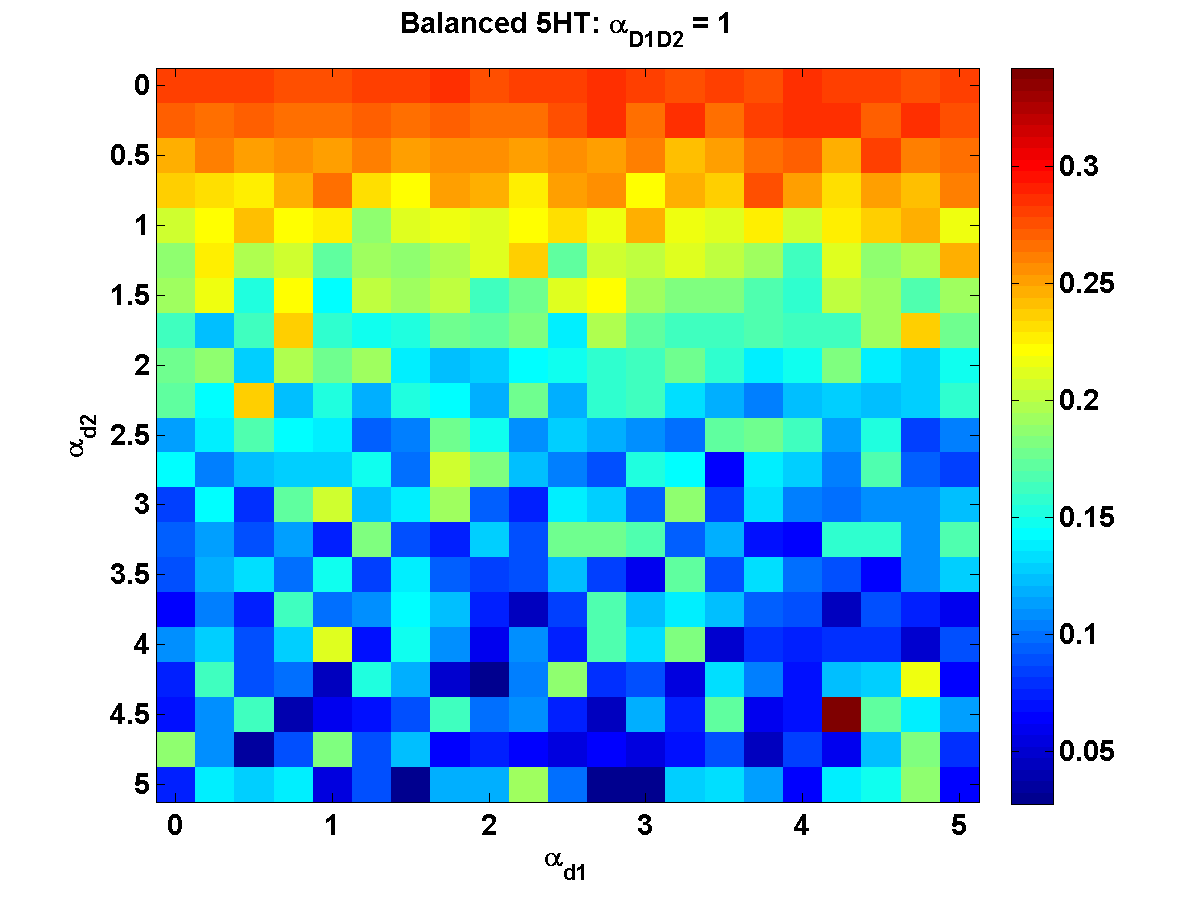  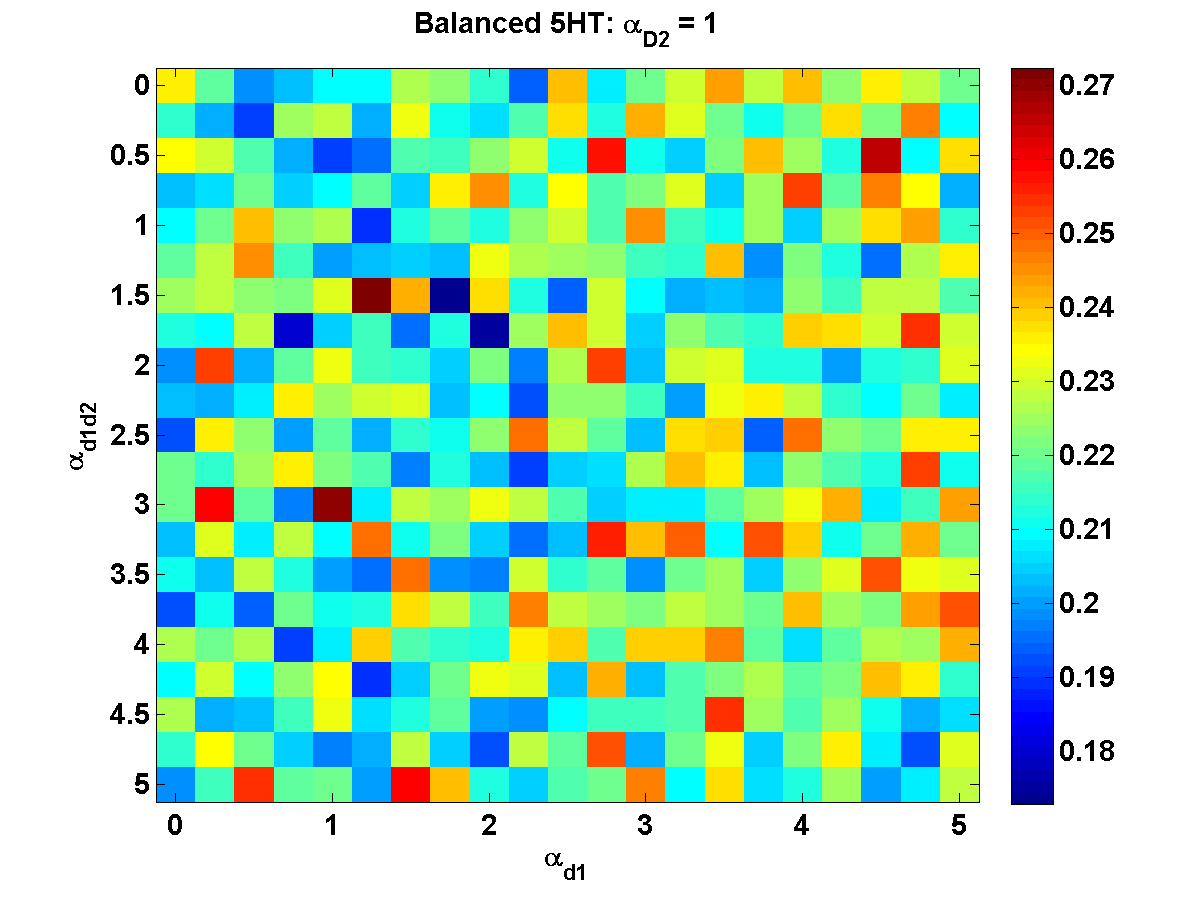  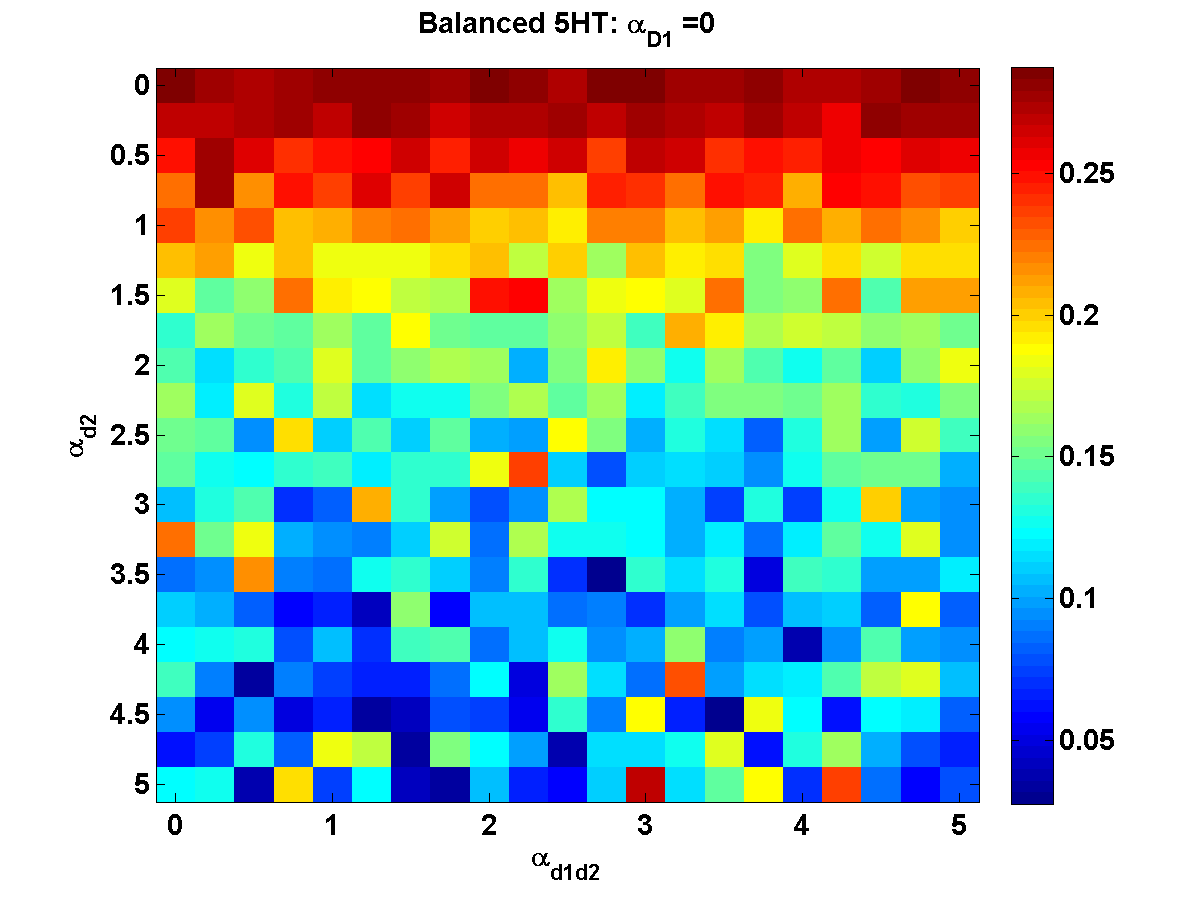  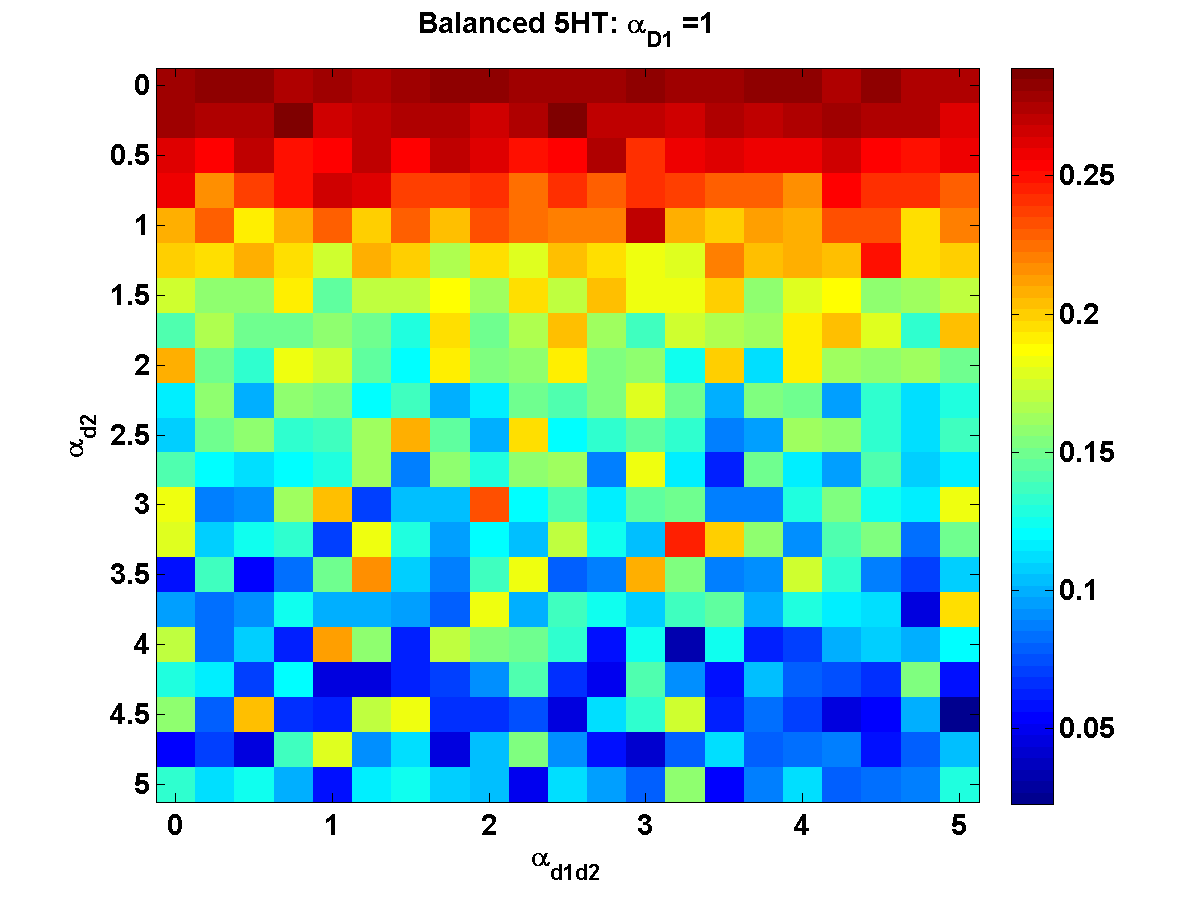  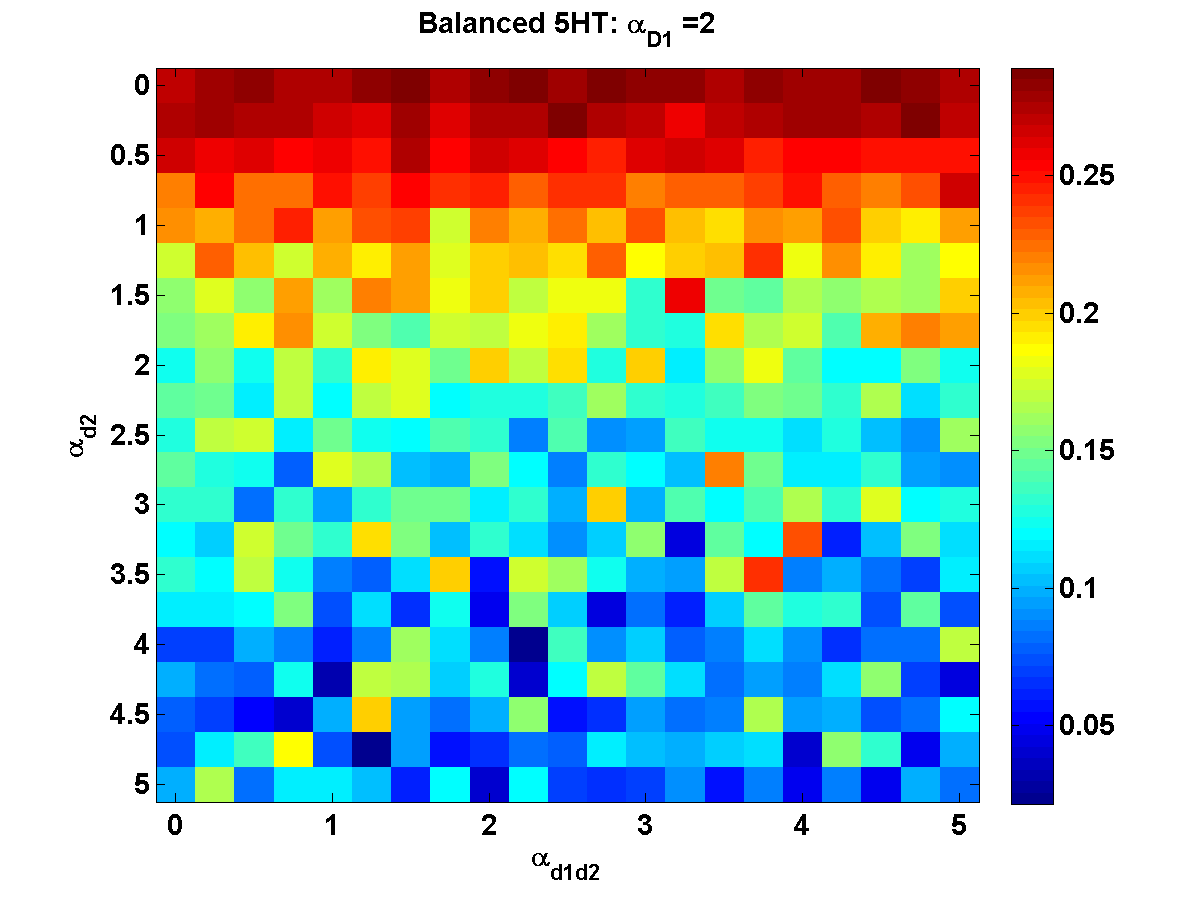  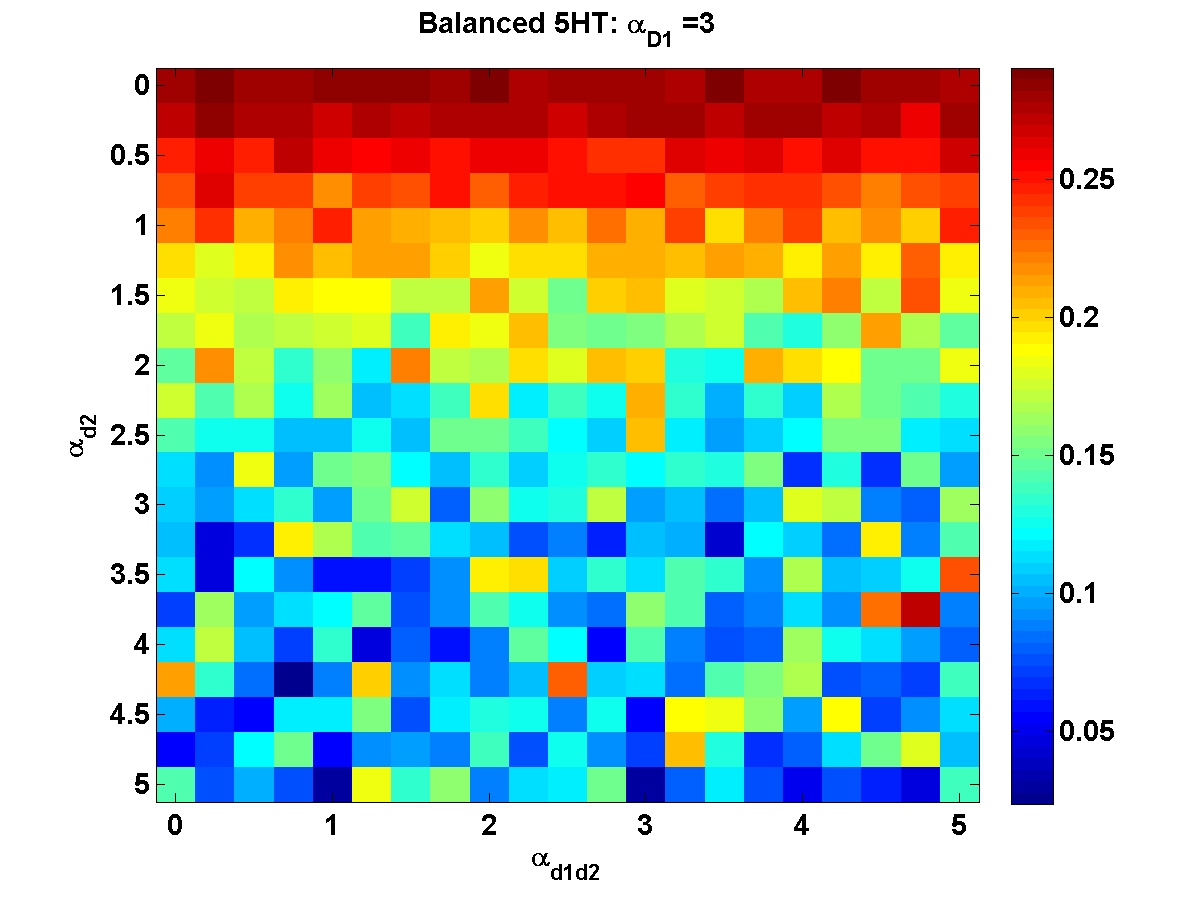  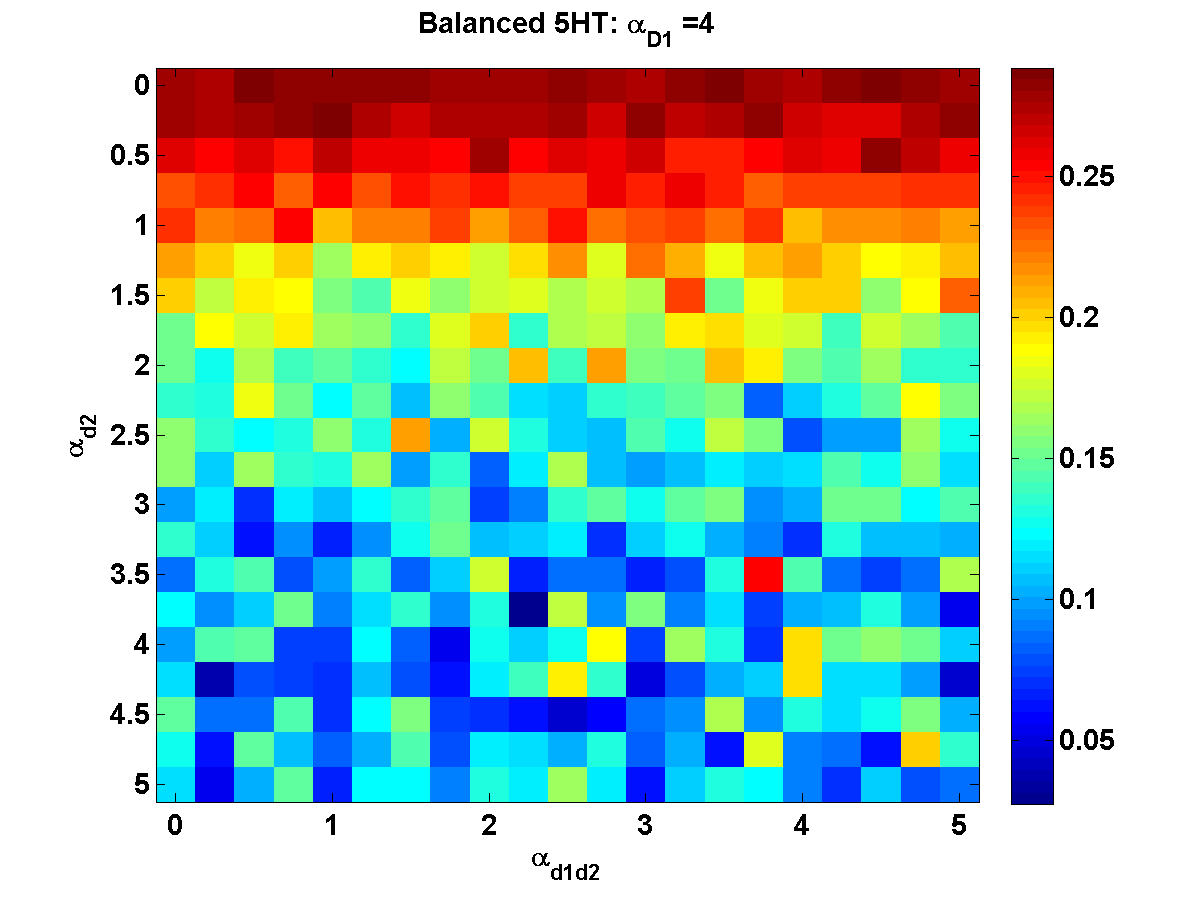  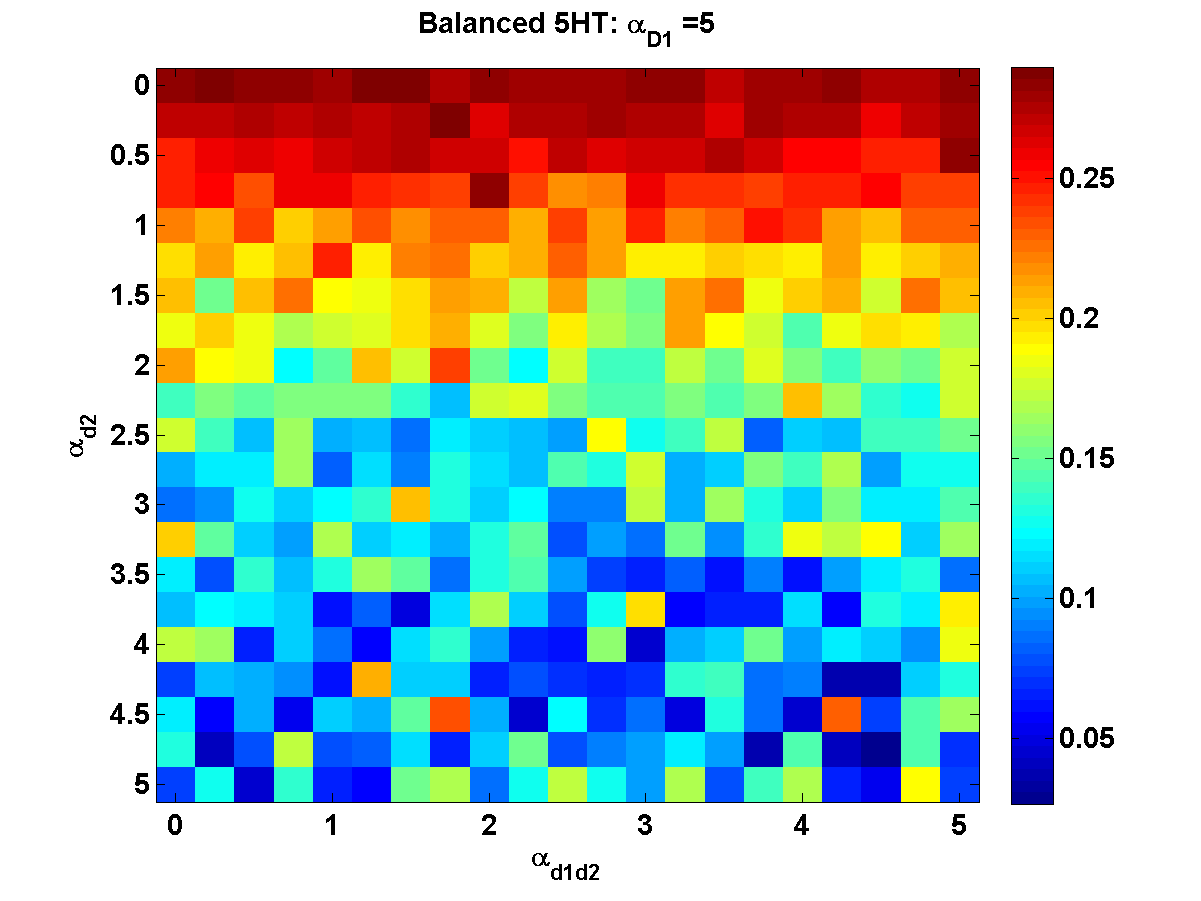 |
| --- |

# Bodi et al. (2009)

Representing normalised Error = ((expt-sims)/expt)^2 summated for the % mean reward [rew] and the % mean punishment [pun] optimality.

Error = ((expt_rew_-sims_rew_)/expt_rew_)^2 + ((expt_pun_-sims_pun_)/expt_pun_)^2

The Expt values are given in the following table:

|  | HC | PD-ON | PD-OFF |
| --- | --- | --- | --- |
| rew | 70.3568 | 74.0769 | 56.3363 |
| pun | 67.3066 | 58.0706 | 74.4182 |

## Healthy controls (HC):

The first row represents cases 1-3 in which the appropriate parameter (noted in the legend for that data plot) is varied, and the others in set (α_D1_, α_D2_, α_D1D2_) are fixed to 1. The subsequent rows show cases 4 and 5 where α_D1D2_ and α_D2_ are fixed to 1 respectively, and the other two parameters vary across axes. The later rows present the more general case 6 as a function of (α_D2_, α_D1D2_), for a given α_D1._

| 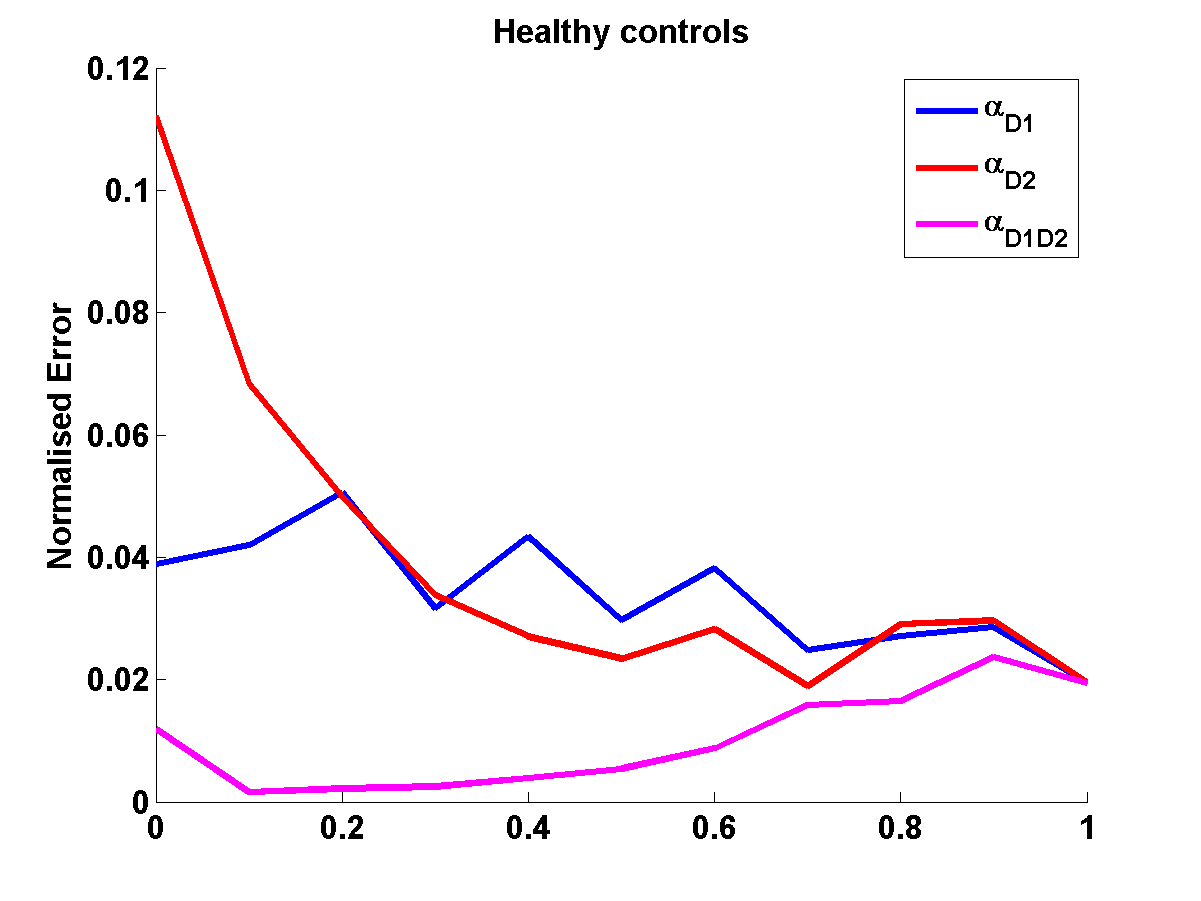  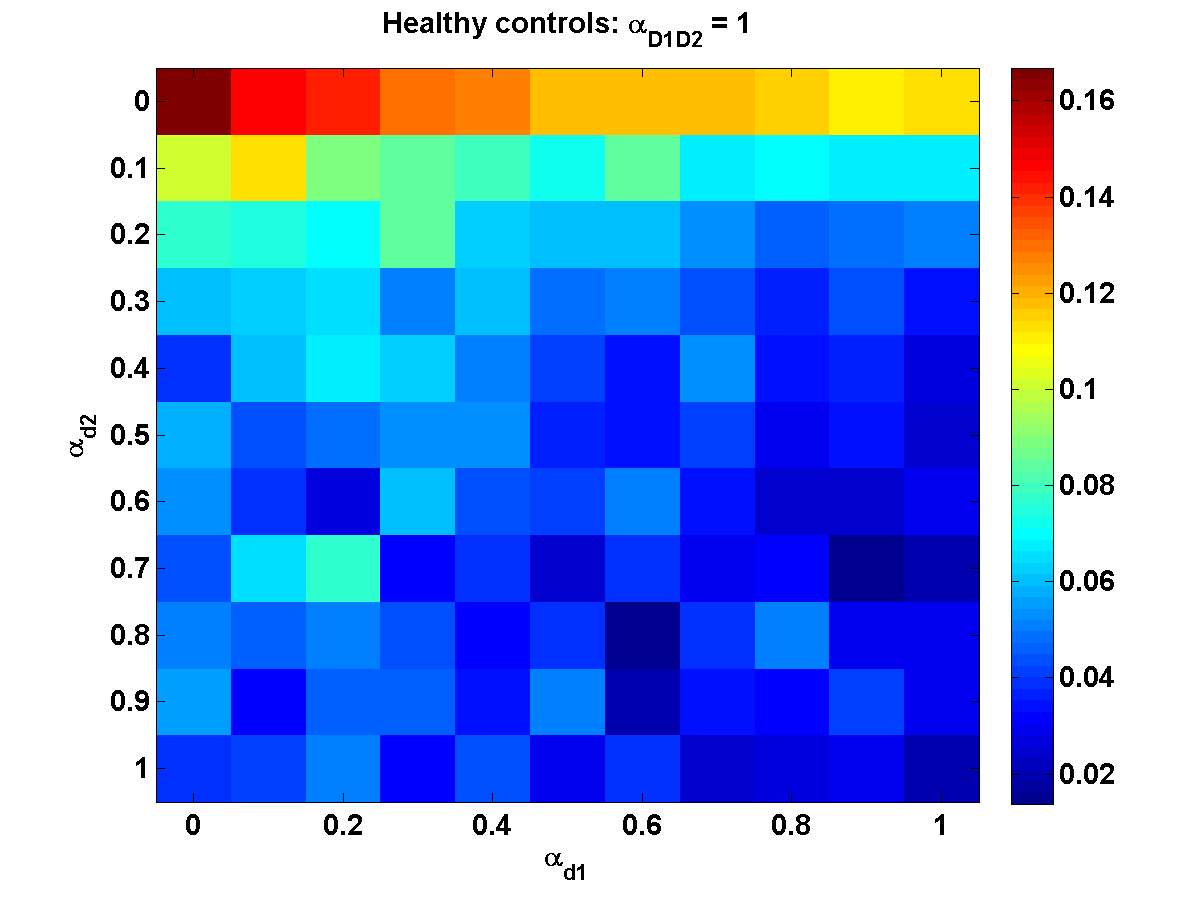  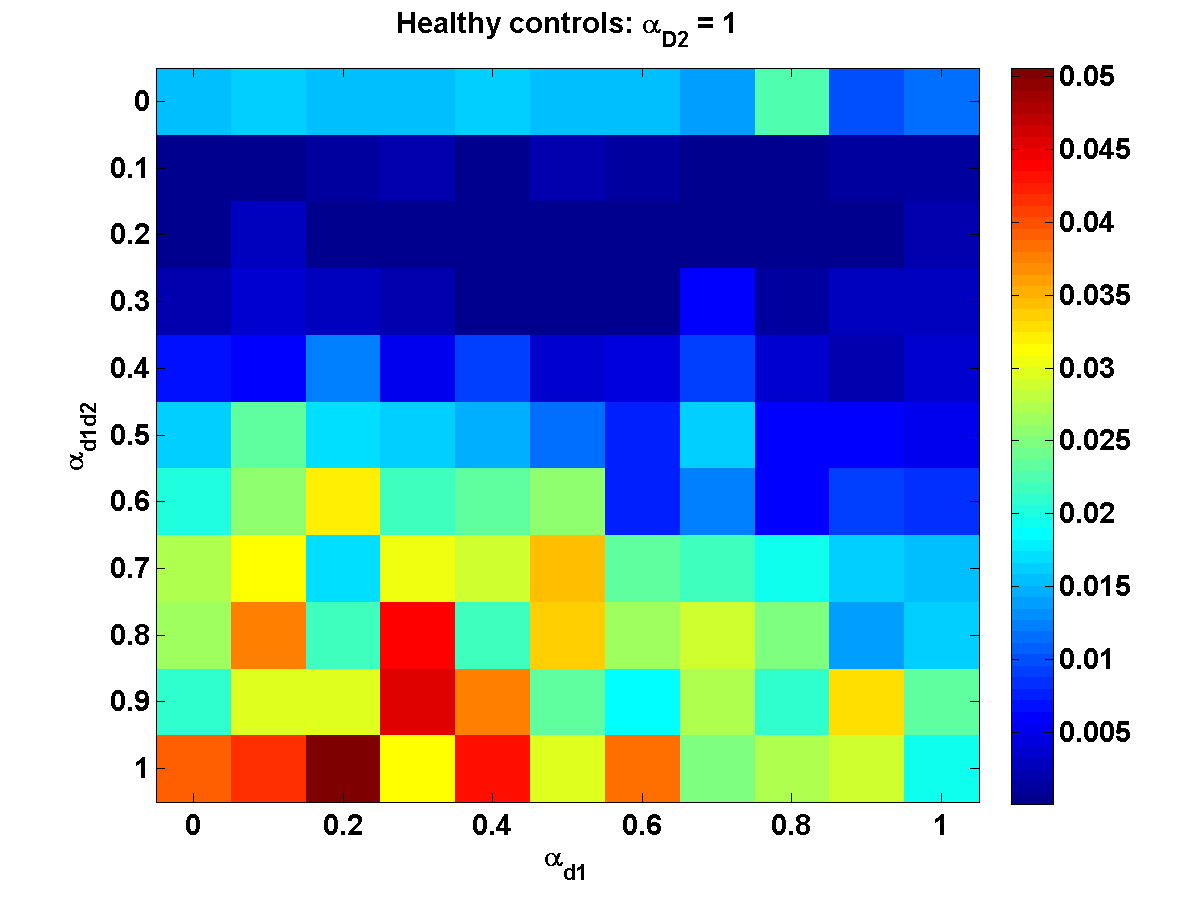  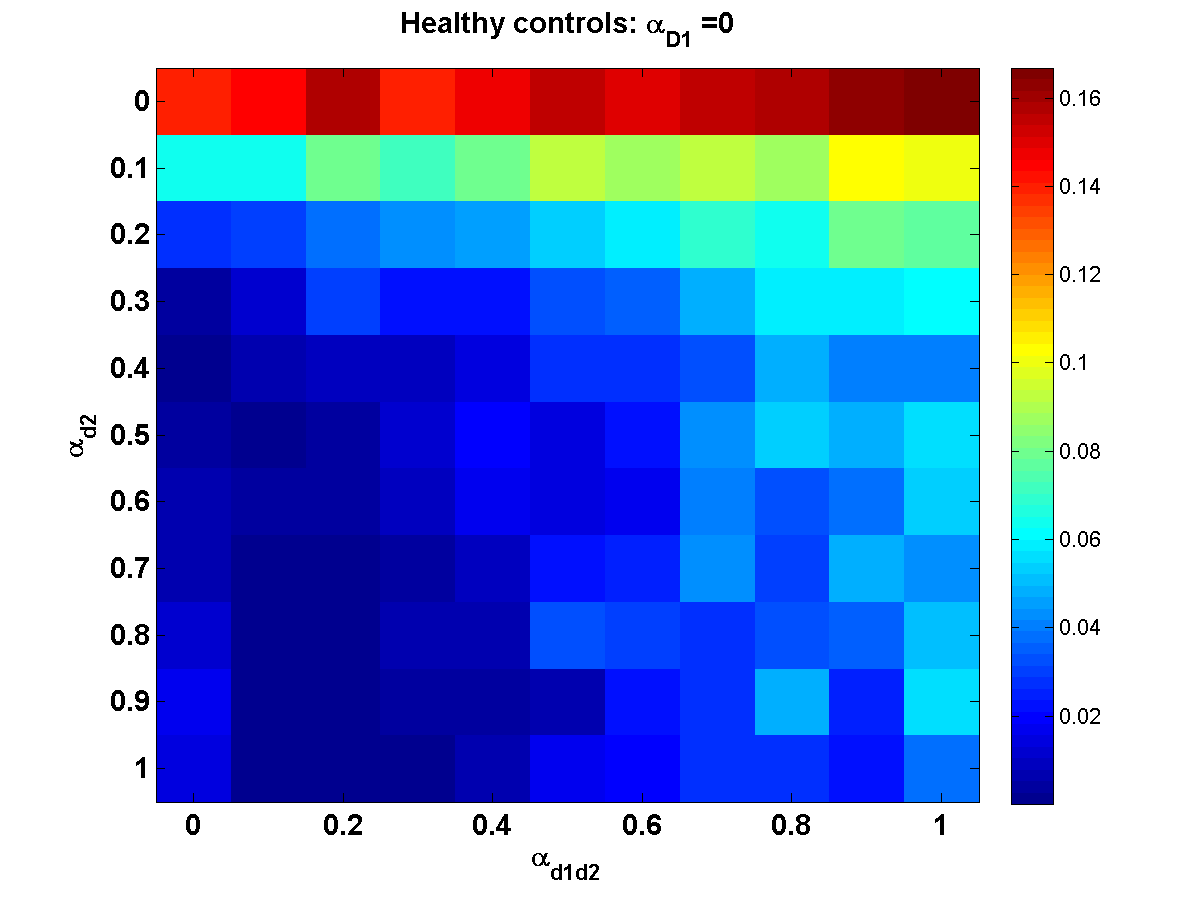  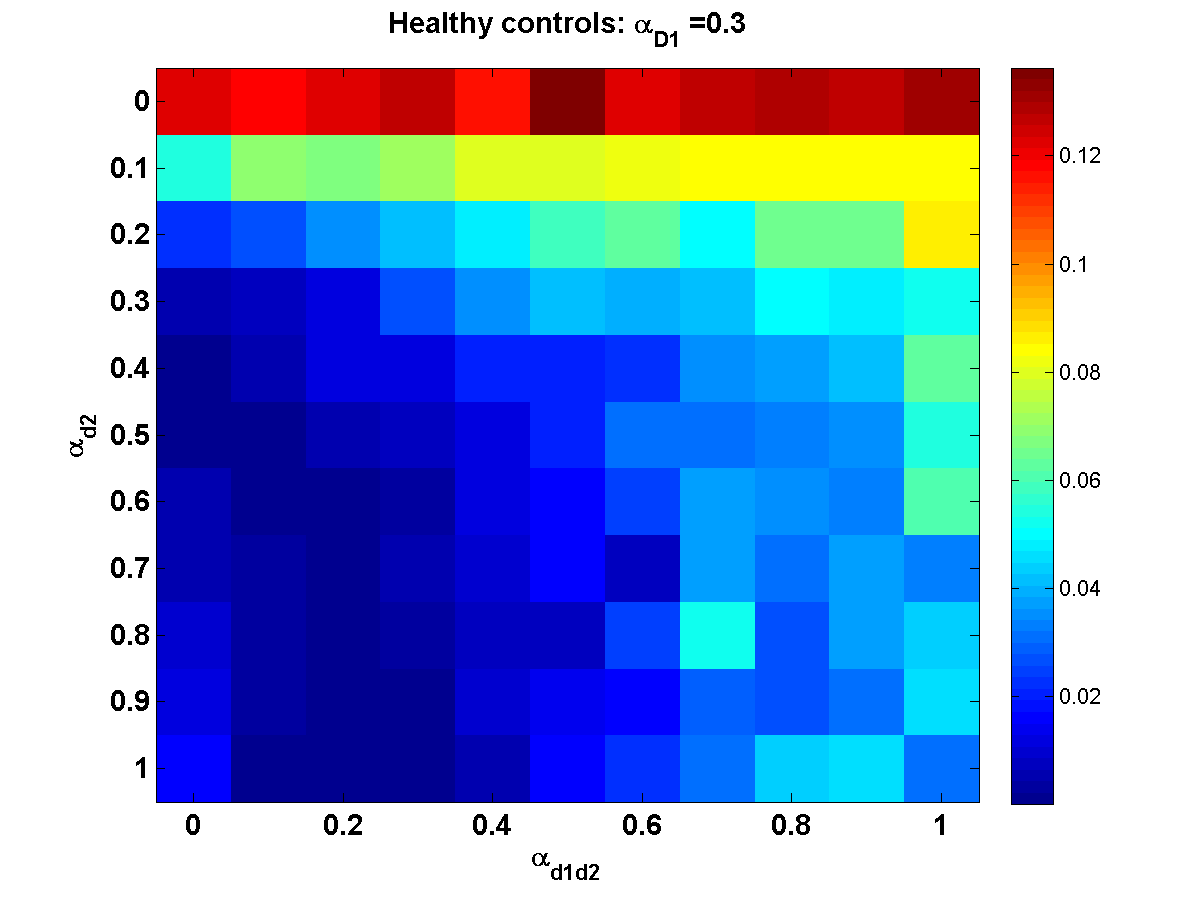  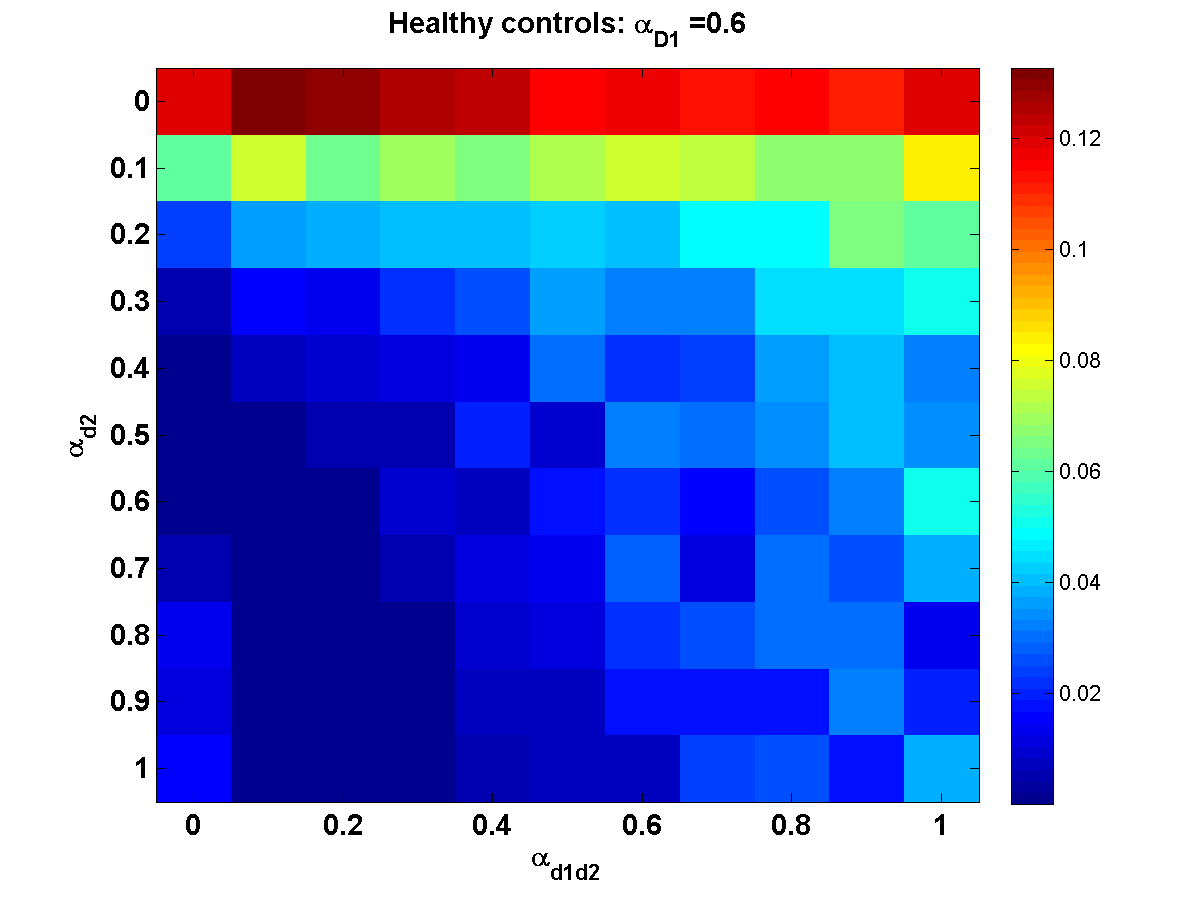  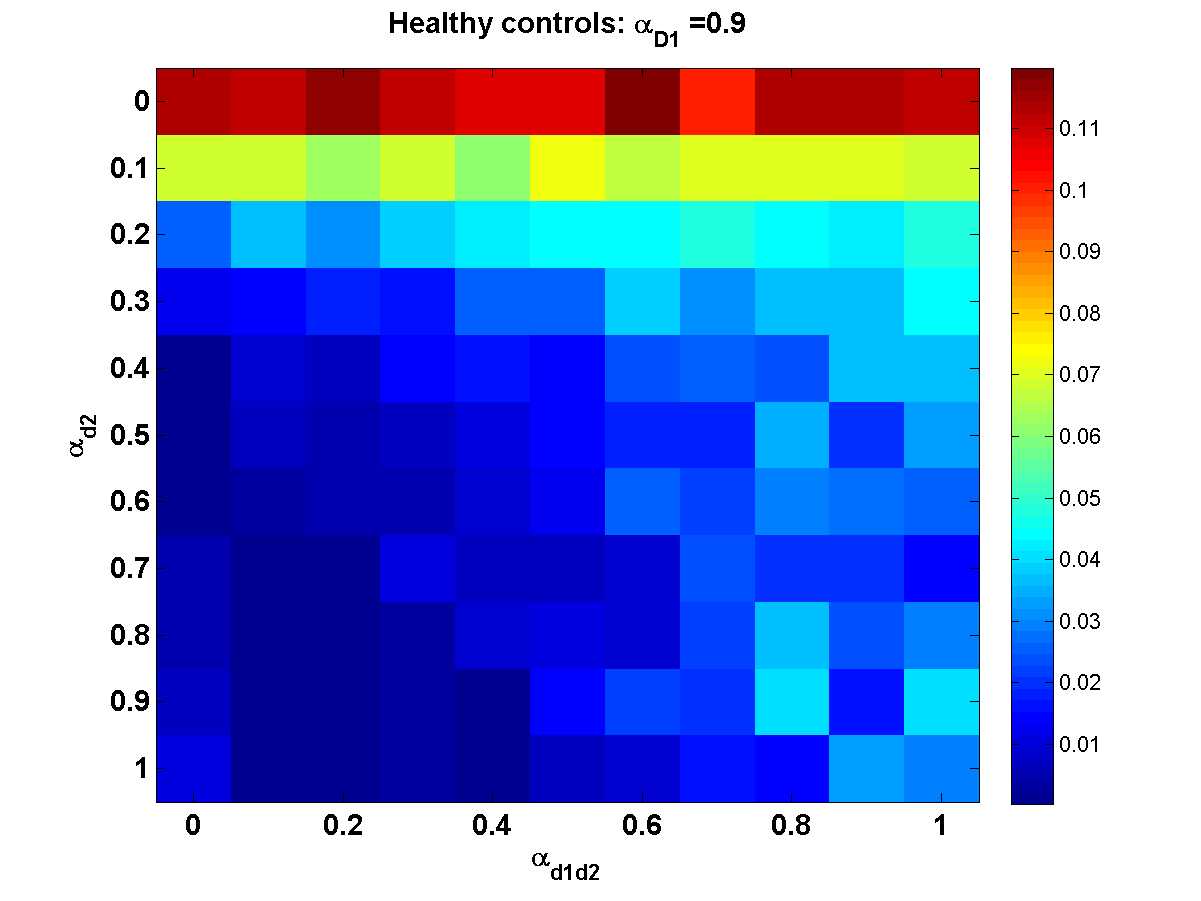 |
| --- |

## PD-ON:

The first row represents cases 1-3 in which the appropriate parameter (noted in the legend for that data plot) is varied, and the others in set (α_D1_, α_D2_, α_D1D2_) are fixed to 1. The subsequent rows show cases 4 and 5 where α_D1D2_ and α_D2_ are fixed to 1 respectively, and the other two parameters vary across axes. The later rows present the more general case 6 as a function of (α_D2_, α_D1D2_), for a given α_D1._

| 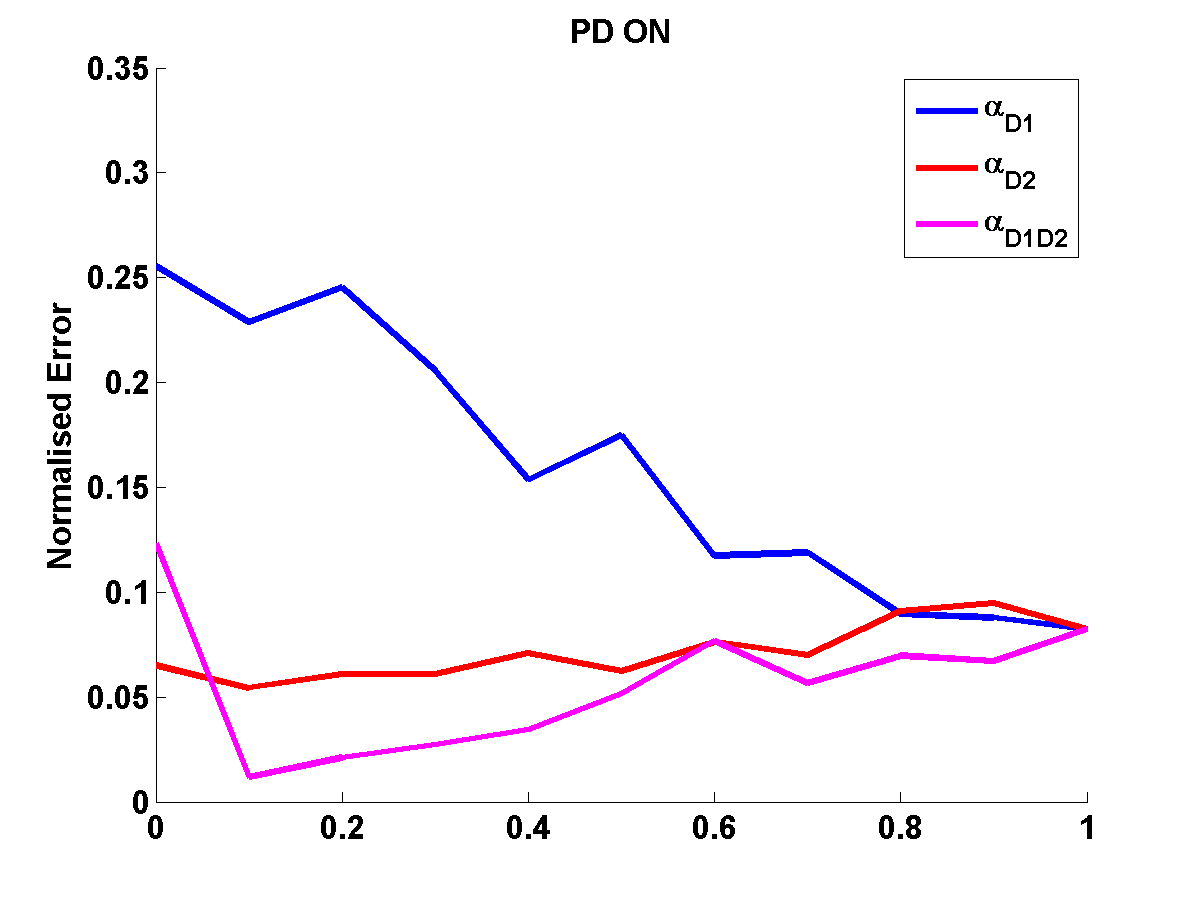  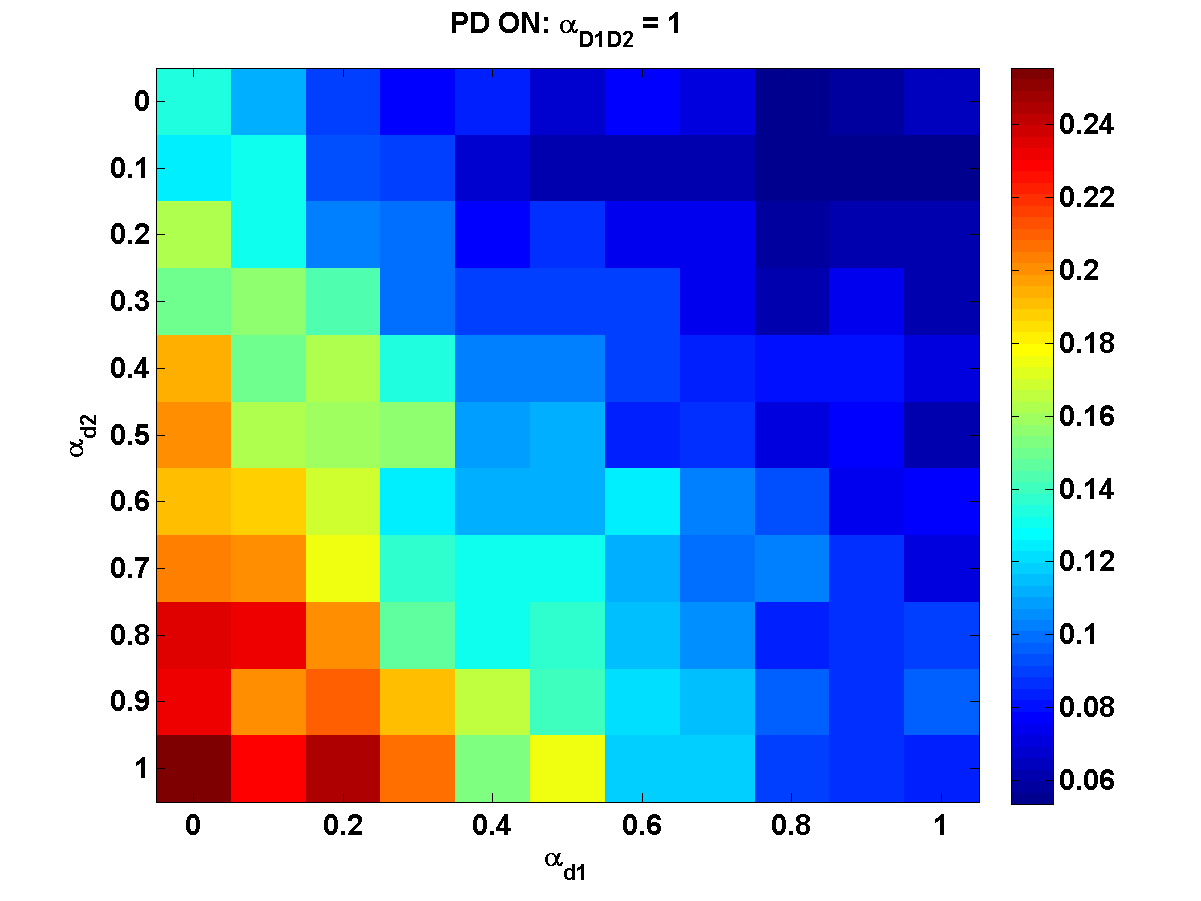  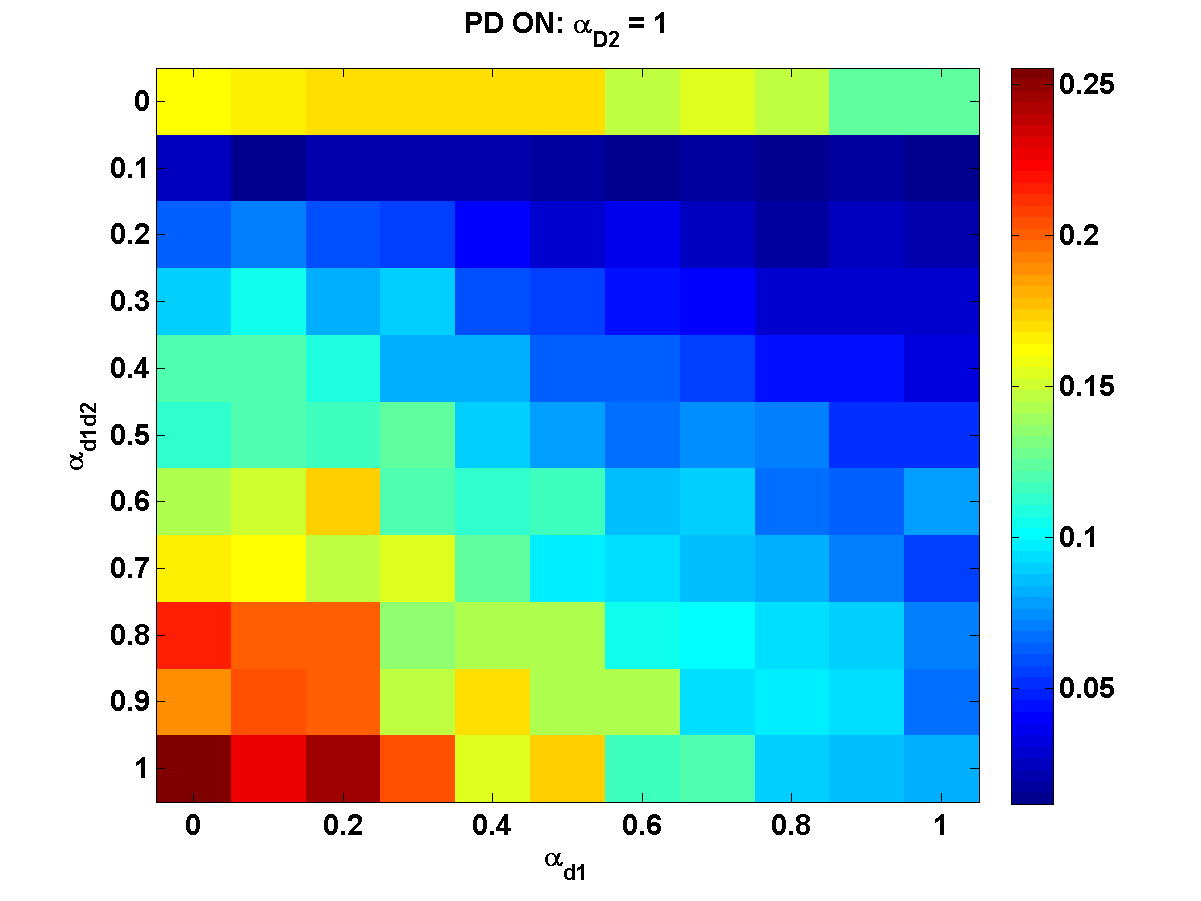  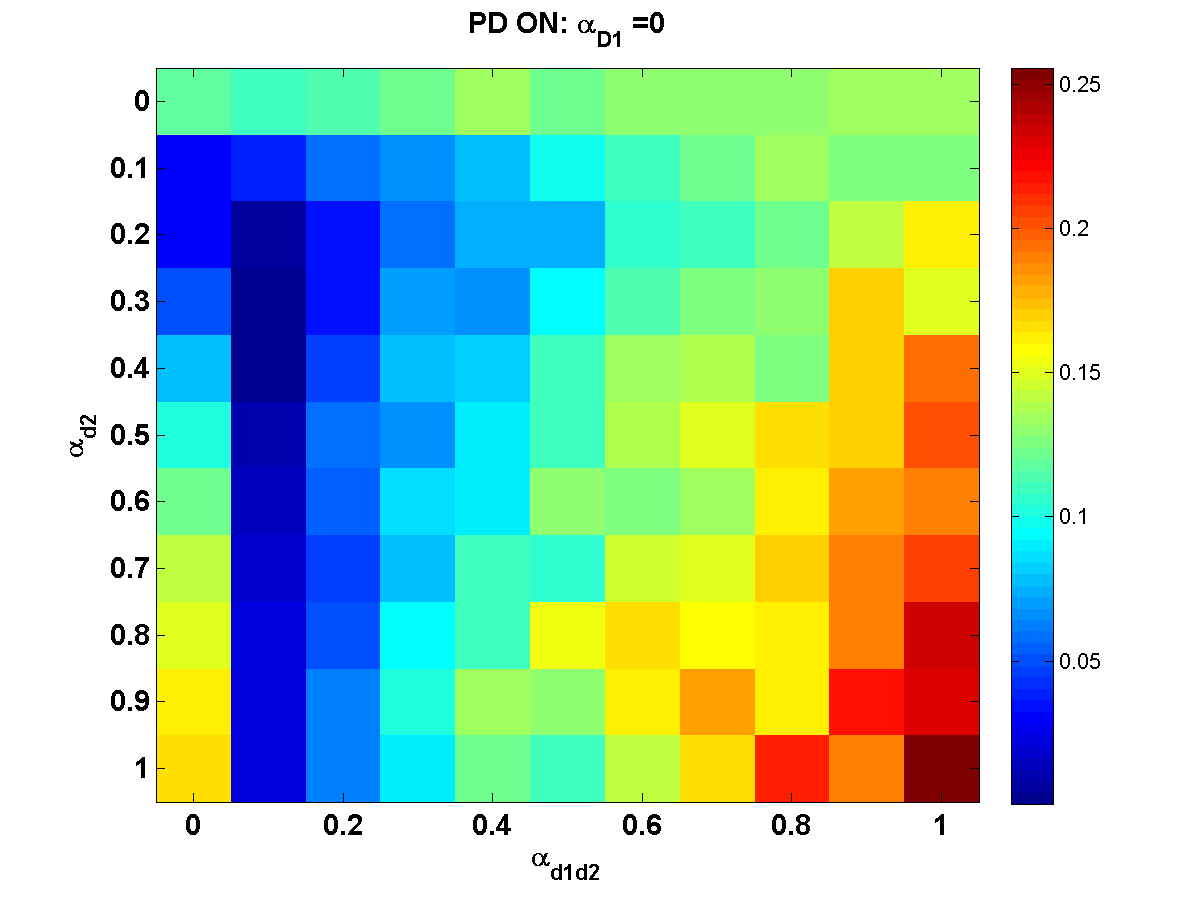  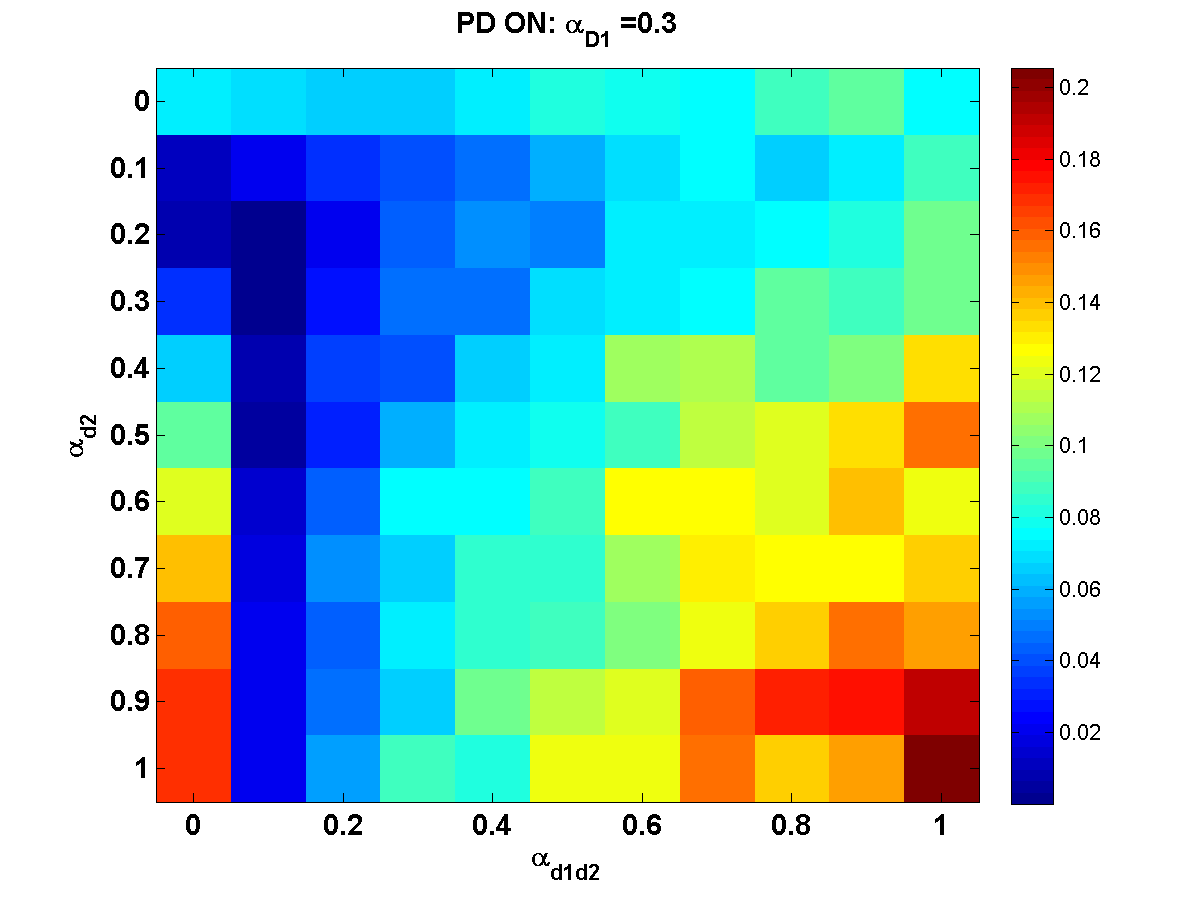  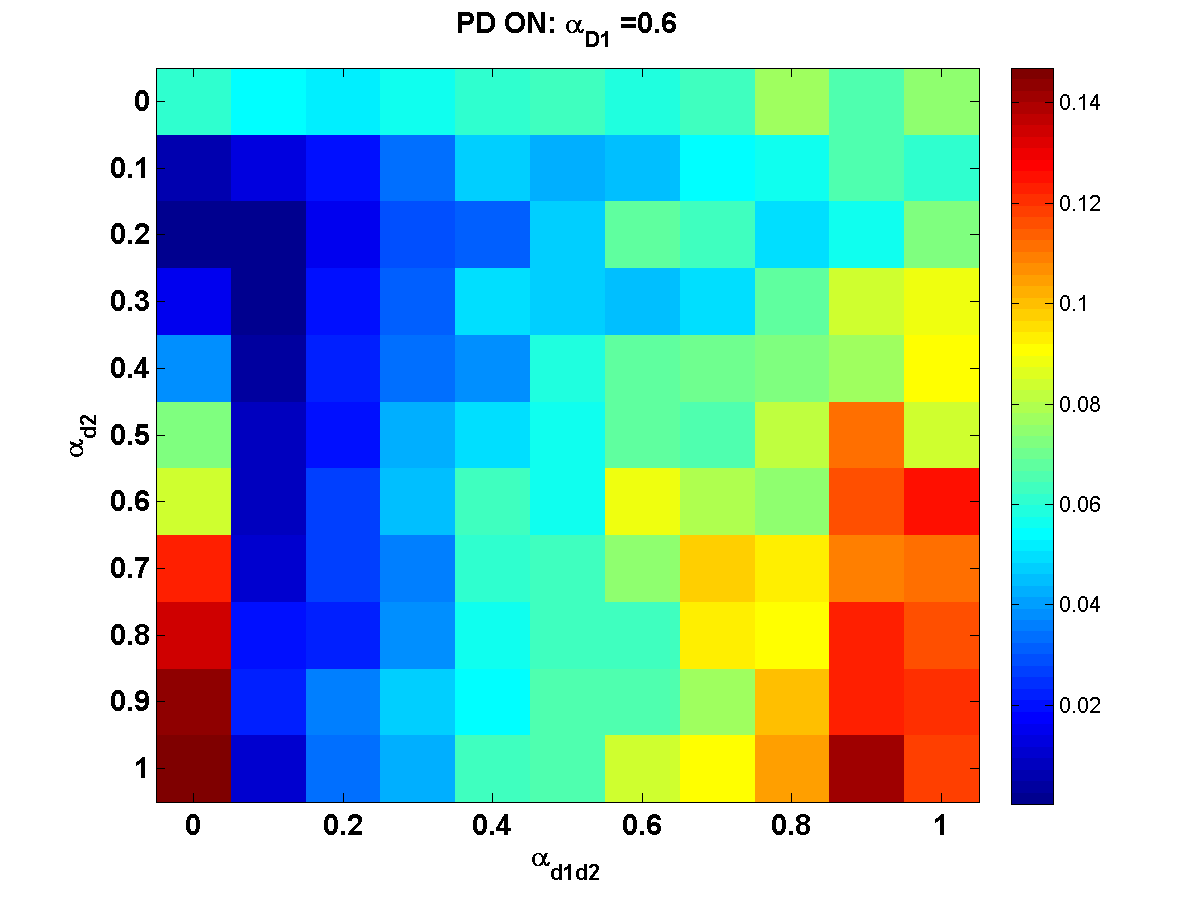  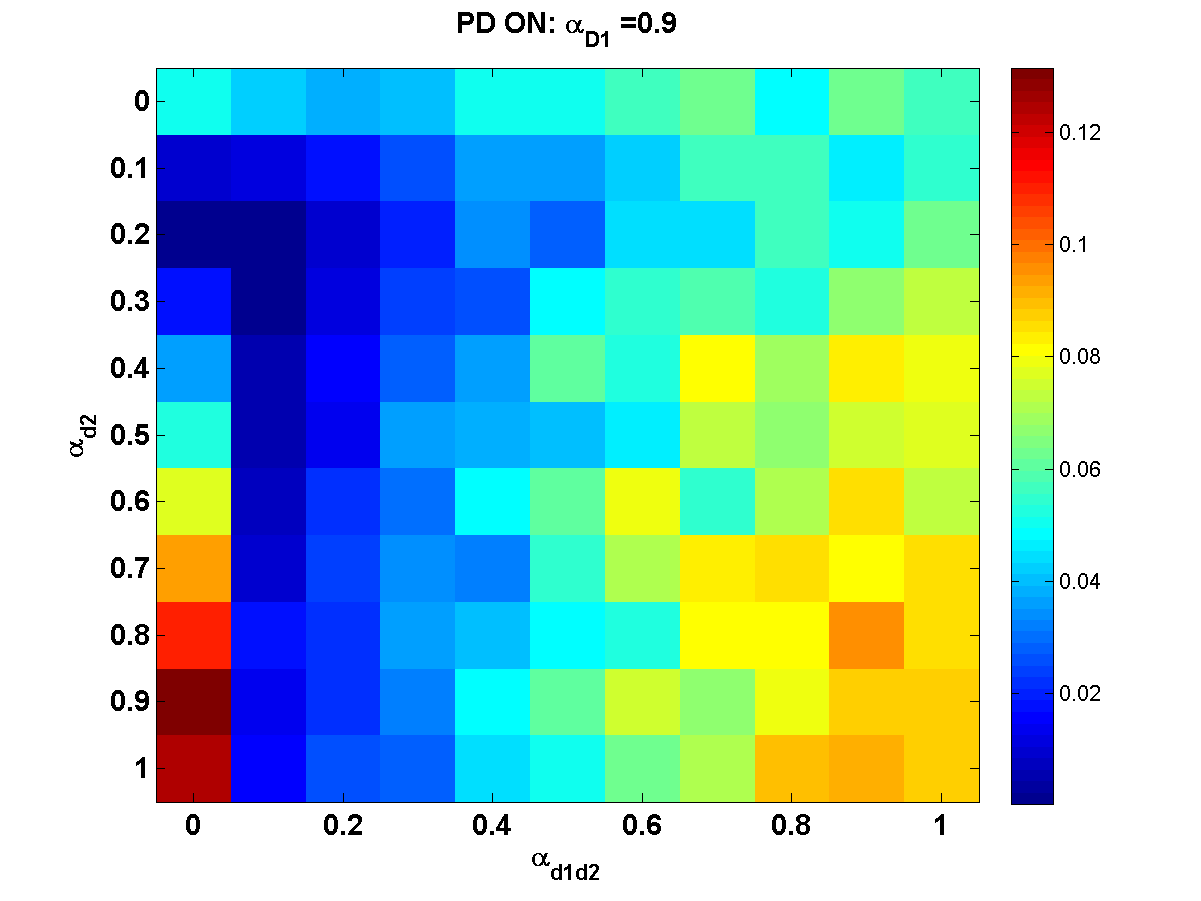 |
| --- |

## PD-OFF:

The first row represents cases 1-3 in which the appropriate parameter (noted in the legend for that data plot) is varied, and the others in set (α_D1_, α_D2_, α_D1D2_) are fixed to 1. The subsequent rows show cases 4 and 5 where α_D1D2_ and α_D2_ are fixed to 1 respectively, and the other two parameters vary across axes. The later rows present the more general case 6 as a function of (α_D2_, α_D1D2_), for a given α_D1._

| 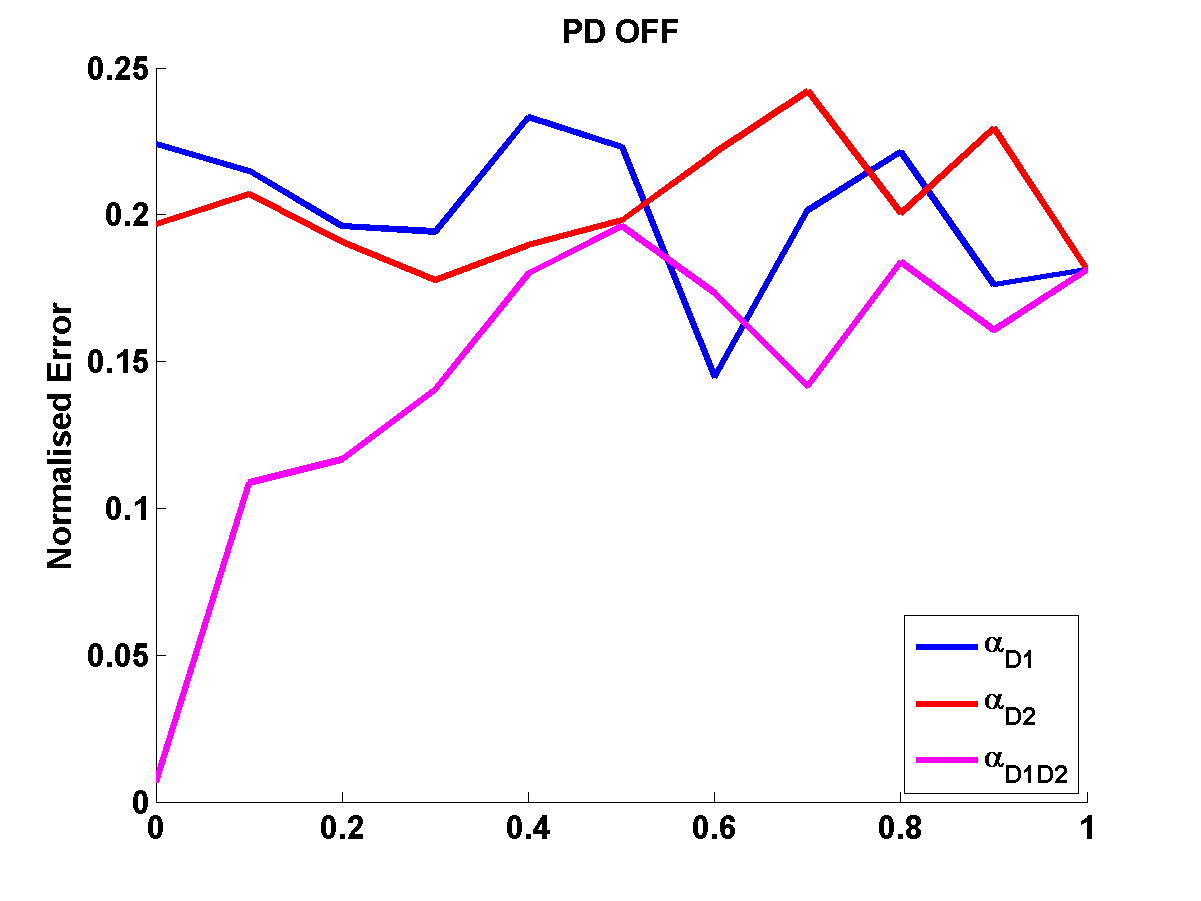  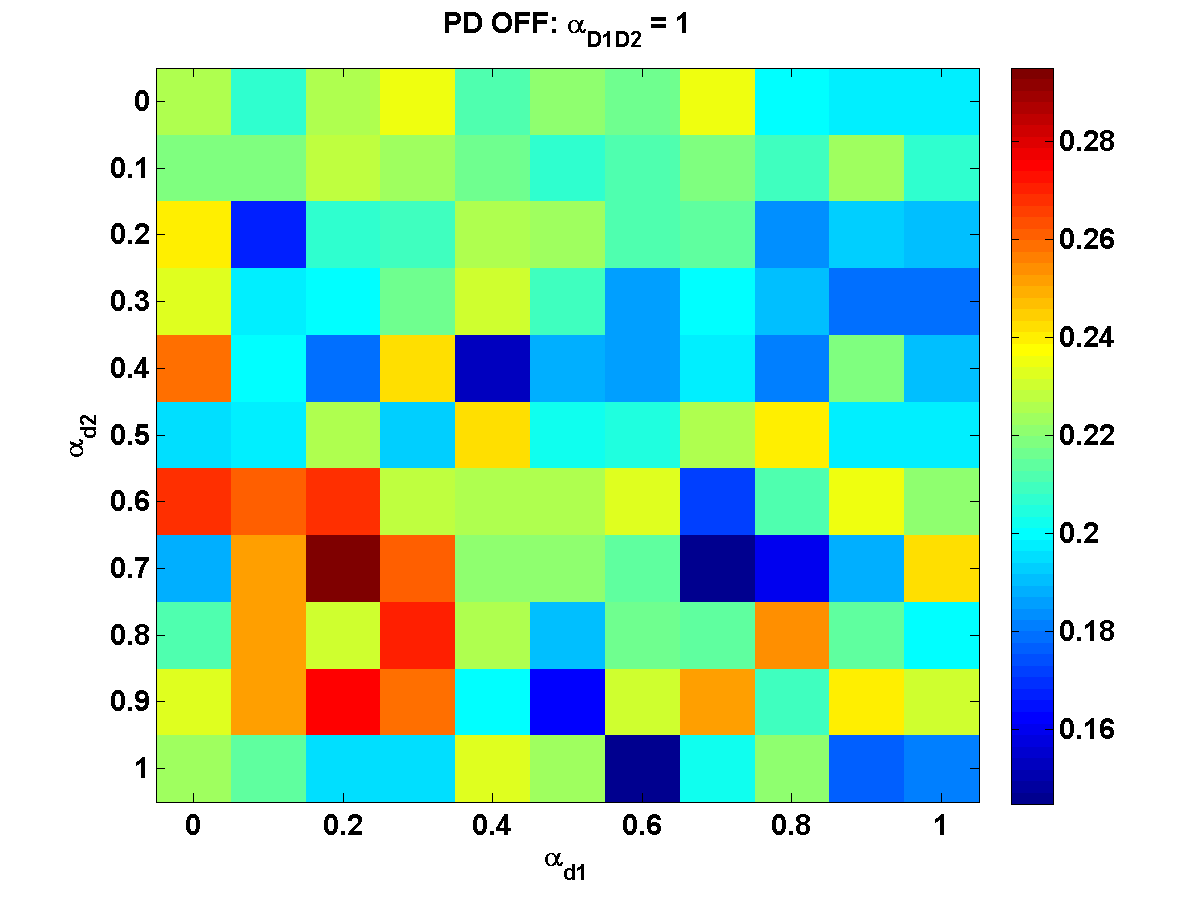  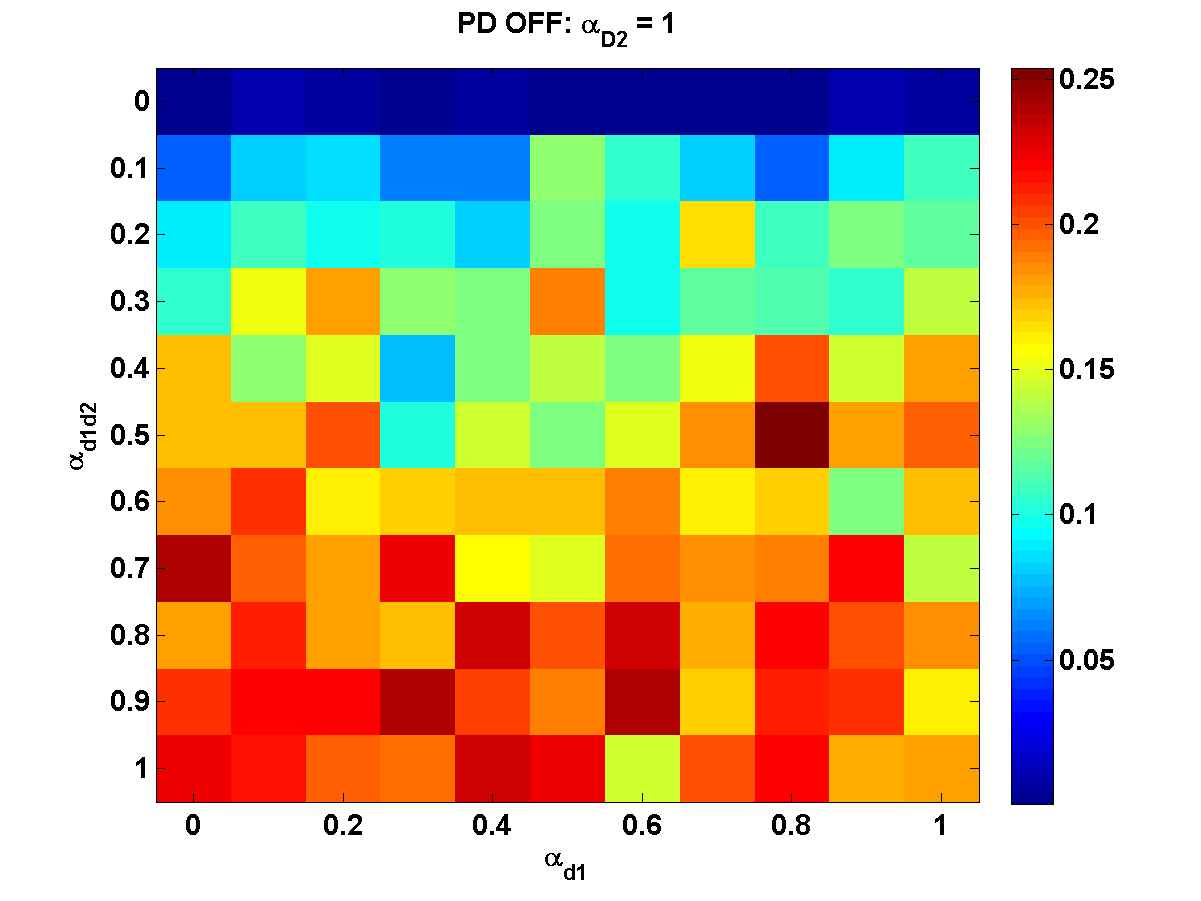  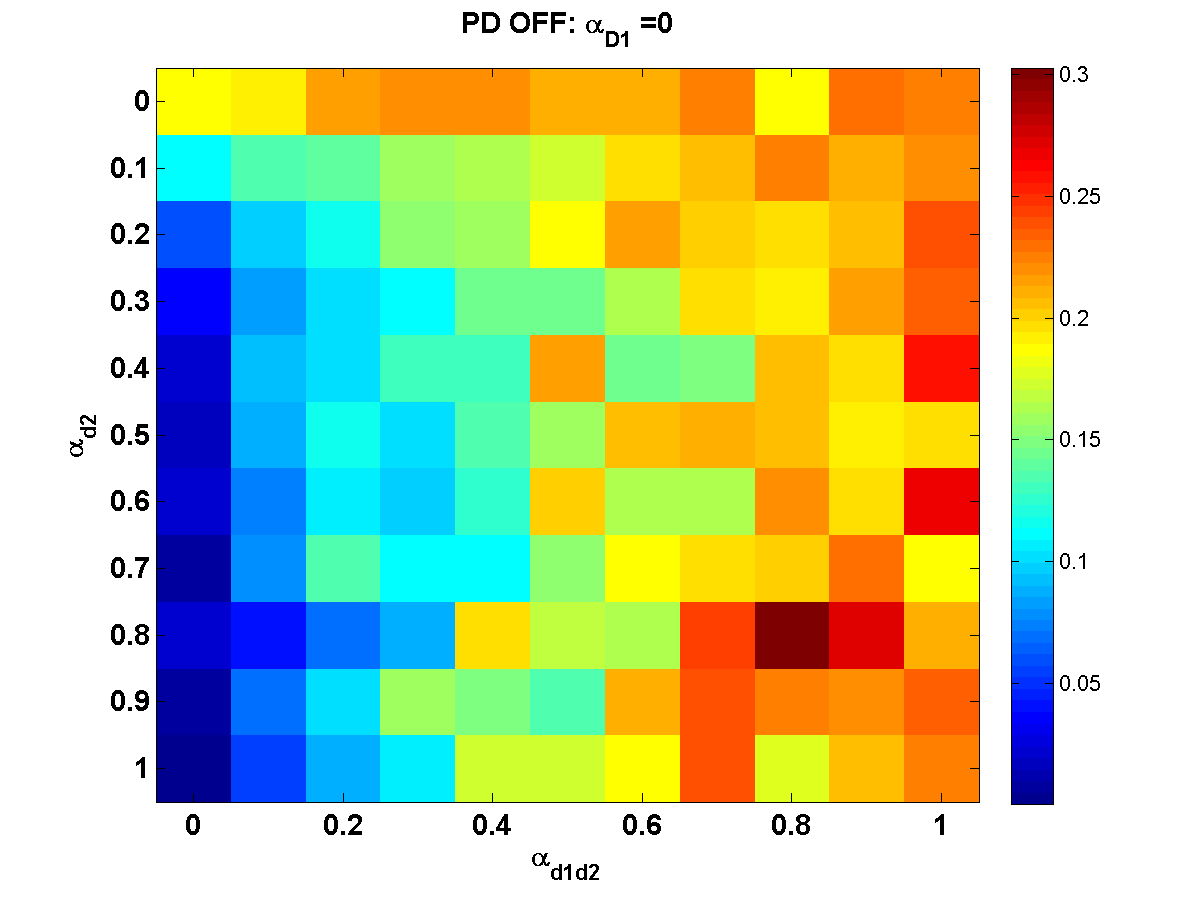  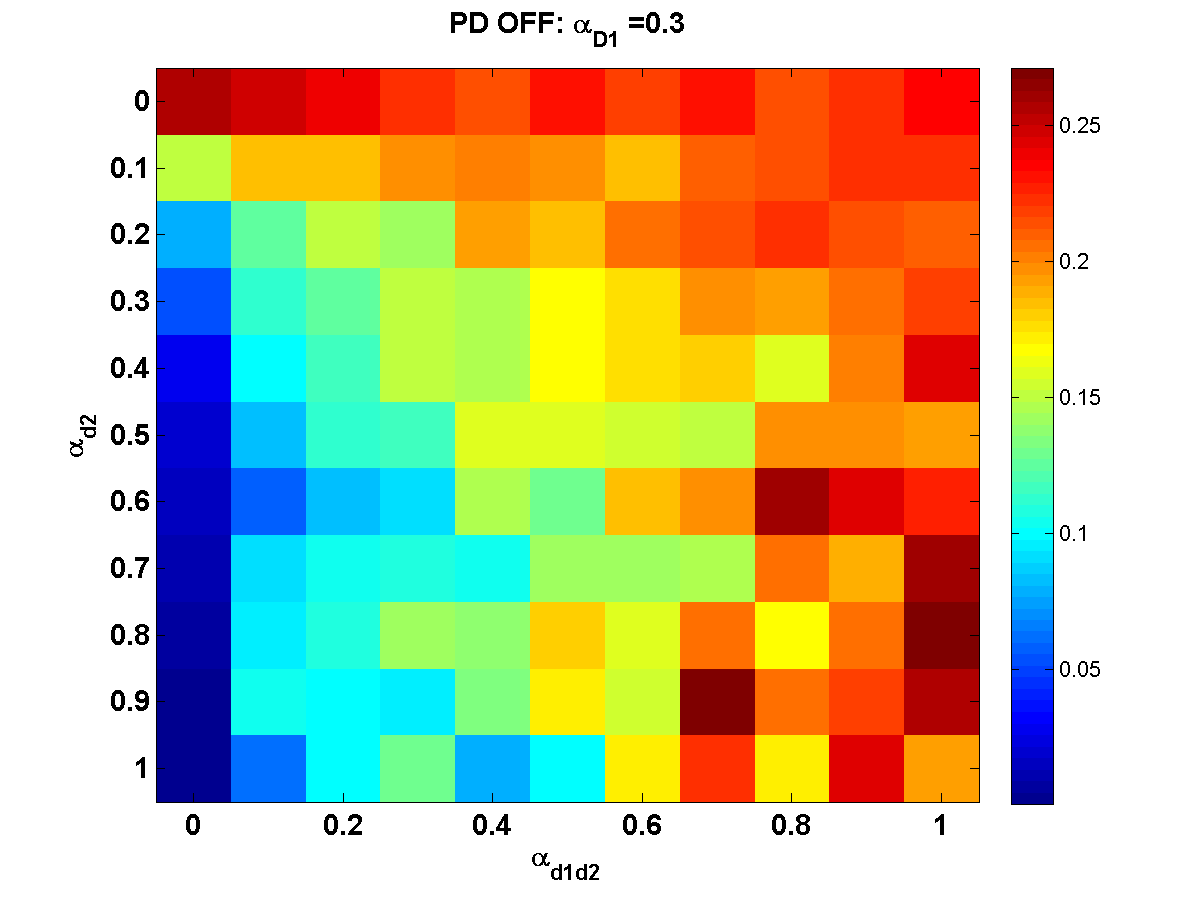  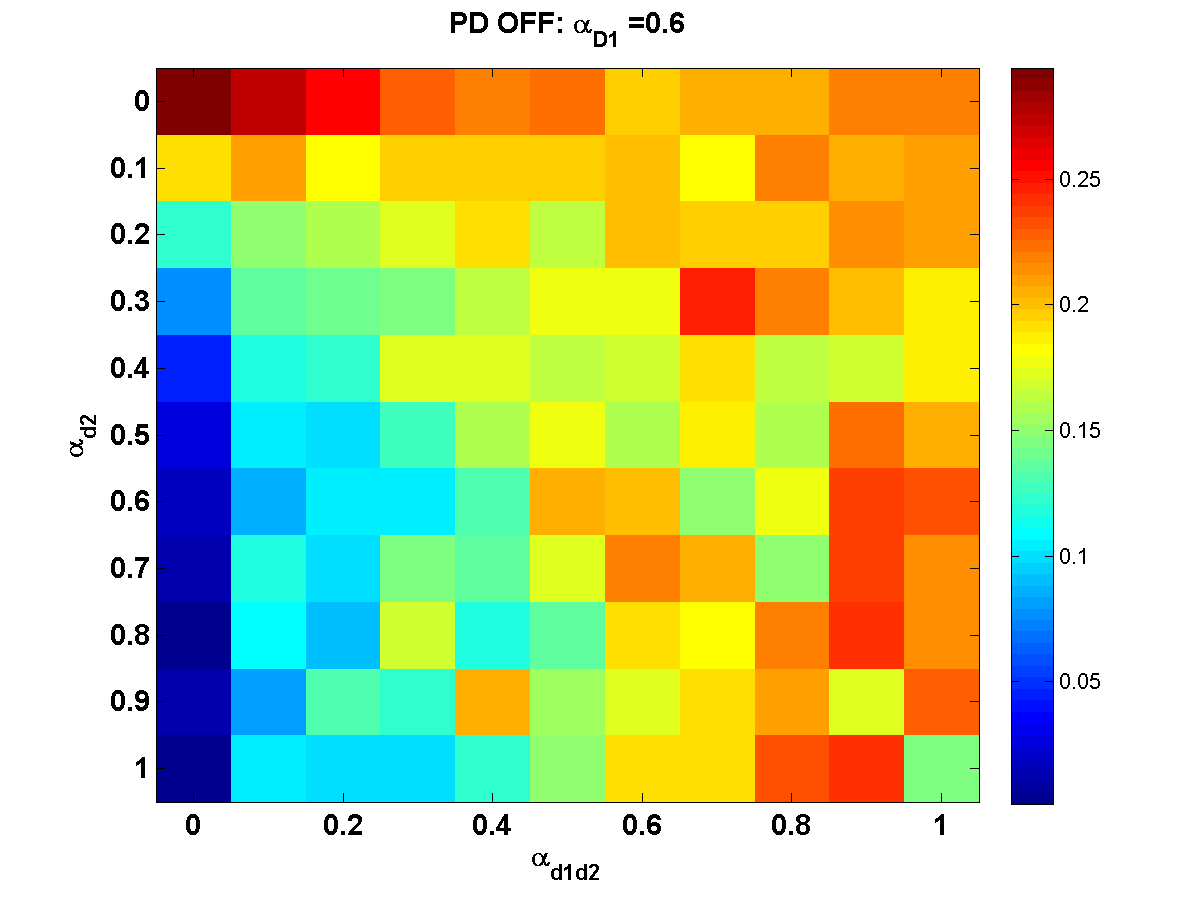  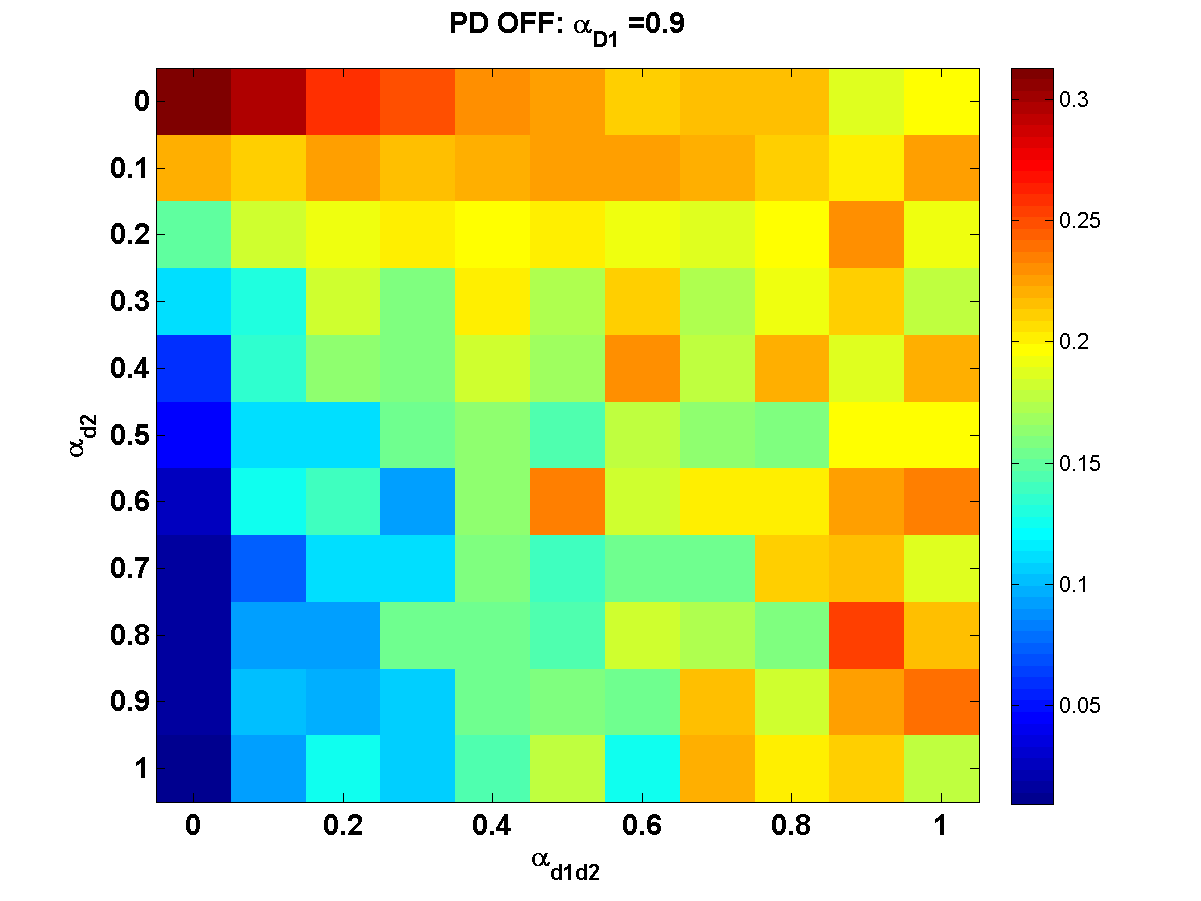 |
| --- |
