# Supplementary material for: A network model of basal ganglia for understanding the roles of dopamine and serotonin in reward-punishment-risk based decision making
Source: Front Comput Neurosci. 2015 Jun 17;9:76. doi: 10.3389/fncom.2015.00076 (PMC4469836; doi:10.3389/fncom.2015.00076)
Supplement: Supplementary file 3 [file DataSheet3.DOCX]

**Supplementary material C:**

(a) Analysis of the effect of 5HT (*α*_D1D2_) on PD patients' sensitivity profile in comparison to that of controls (b) Analysis of the effect of 5HT (*α*_D1D2_) on PD patients' sensitivity profile in comparison to that of controls, with no *sign()* term in the eqn. (2.3.9).

**
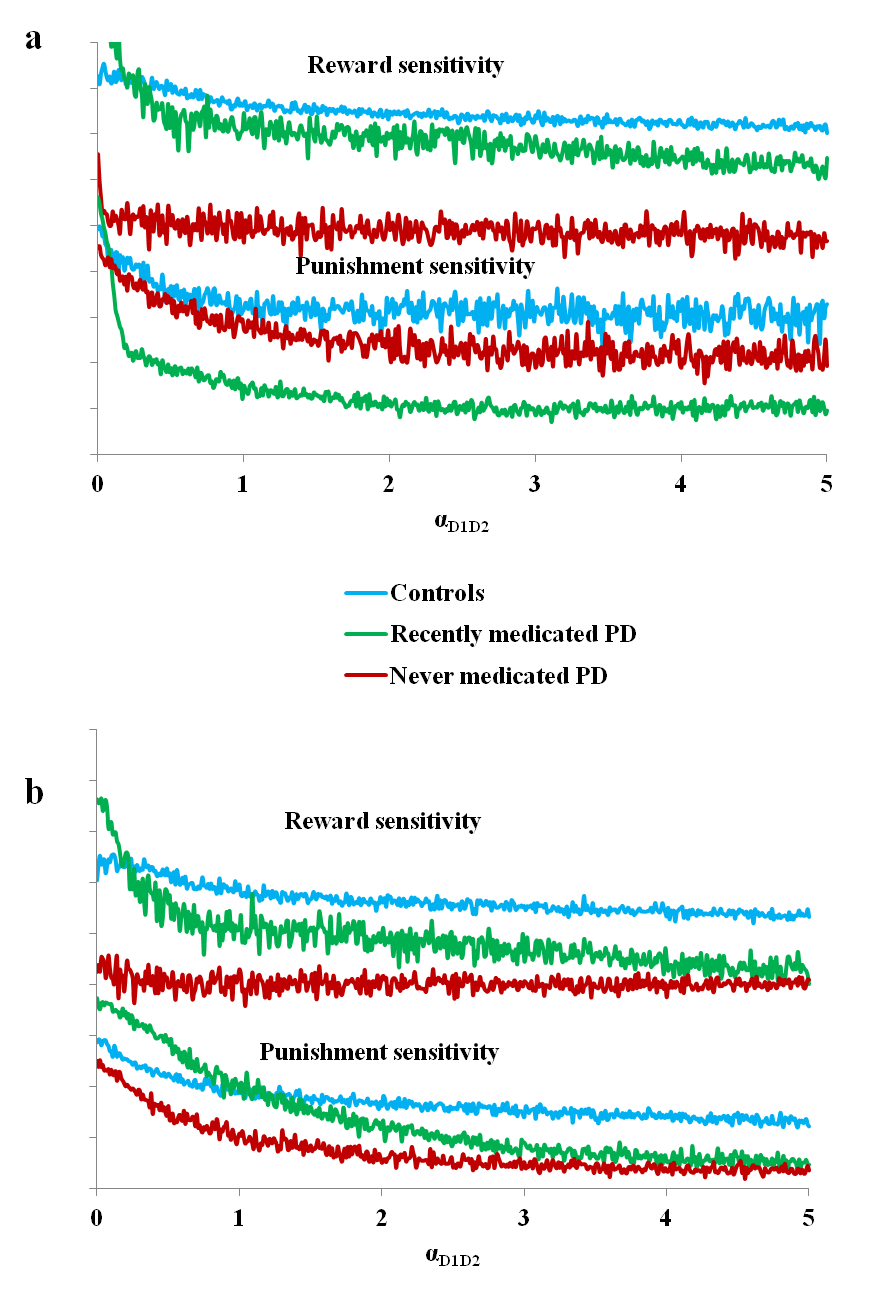
**
